# Environmentally Sensitive Color‐Shifting Fluorophores for Bioimaging

**DOI:** 10.1002/anie.202008357

**Published:** 2020-09-28

**Authors:** Lu Wang, Julien Hiblot, Christoph Popp, Lin Xue, Kai Johnsson

**Affiliations:** ^1^ Department of Chemical Biology Max Planck Institute for Medical Research Jahnstrasse 29 69120 Heidelberg Germany; ^2^ Institute of Chemical Sciences and Engineering École Polytechnique Fédérale de Lausanne (EPFL) 1015 Lausanne Switzerland

**Keywords:** bioimaging, color-shifting, fluorophores, ratiometric probes, sensors

## Abstract

We introduce color‐shifting fluorophores that reversibly switch between a green and red fluorescent form through intramolecular spirocyclization. The equilibrium of the spirocyclization is environmentally sensitive and can be directly measured by determining the ratio of red to green fluorescence, thereby enabling the generation of ratiometric fluorescent probes and biosensors. Specifically, we developed a ratiometric biosensor for imaging calcium ions (Ca^2+^) in living cells, ratiometric probes for different proteins, and a bioassay for the quantification of nicotinamide adenine dinucleotide phosphate.

## Graphical abstract

Environmentally sensitive color‐shifting fluorophores that reversibly switch between a green and red fluorescent form were developed. The equilibrium of the spirocyclization is environmentally sensitive, thereby enabling the generation of ratiometric fluorescent probes and biosensors, including probes for imaging calcium ions (Ca^2+^) in living cells and a bioassay for the quantification of NADPH.

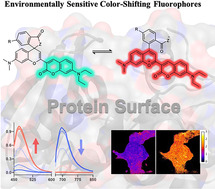

Studying biological processes in living cells through fluorescence imaging depends on the availability of suitable fluorescent probes. Rhodamines are very bright and photostable fluorophores and currently represent the most prominent class of fluorophores for the development of such probes.^[1]^ One of the key properties of rhodamines is that they exist in an equilibrium between a nonfluorescent, spirocyclic form and a fluorescent zwitterionic form (Figure 1 a).^[2]^ The non‐charged spirocyclic form of rhodamines is responsible for the relatively good cell permeability of rhodamine‐based probes.[^1b^, ^3^] Furthermore, the equilibrium between the spirocyclic and the zwitterionic form of rhodamines is environmentally sensitive and for numerous rhodamine‐based probes binding to their biological target results in a shift of the equilibrium from the non‐fluorescent to the fluorescent form.^[1b]^ The resulting fluorogenicity of such probes increases the signal‐to‐noise ratio in imaging experiments. An understanding of how the binding of various rhodamine‐based probes to proteins shifts their equilibrium between the spirocyclic form and the zwitterionic form towards the zwitterion is hampered by the lack of structural information in most cases.^[1b]^ However, a recently published crystal structure of tetramethylrhodamine bound to the self‐labeling protein HaloTag7 reveals specific interactions between the zwitterionic fluorophore and the protein surface.^[4]^ Furthermore, the equilibrium between the spirocyclic and the zwitterionic form of protein‐bound rhodamines was recently shown to be dependent on the conformation of the protein itself, a feature that can be exploited to generate new classes of protein biosensors.^[4]^

**Figure 1:**
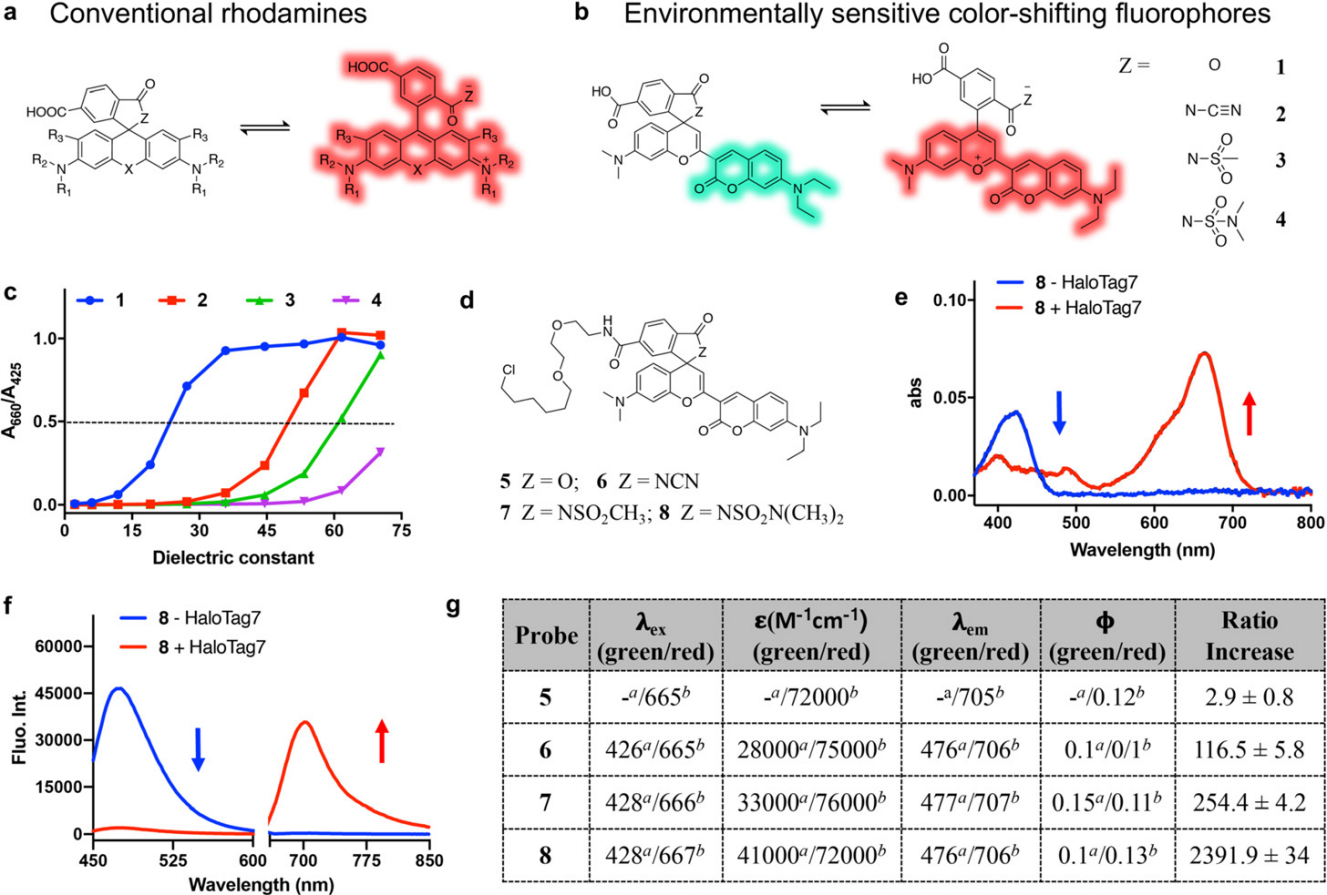
Environmentally sensitive color‐shifting fluorophores (CSFs). a) Structure of conventional rhodamines in equilibrium between the non‐fluorescent spirocyclic and fluorescent zwitterionic forms. b) Structure of the color‐shifting fluorophores in equilibrium between green fluorescent spirocyclic and red fluorescent zwitterionic forms. c) Normalized ratio of absorbance in red/green channels (A_650_/A_420_) of **1**–**4** (10 μM) as a function of dielectric constant (water‐dioxane mixture). The ratios of **1**–**4** were normalized to the maximum ratio of **1**. d) Chemical structures of **5**–**8** for HaloTag7 labeling. e, f) Absorption (e) and fluorescence emission (f) spectra of **8** (1 μM) measured in the absence (blue line) and presence of HaloTag7 (5 μM, red line). g) Photophysical properties of **5**–**8** and fluorescence ratio (F_707_/F_475_) change induced upon HaloTag7 labeling. Data were collected in absence [a] and presence [b] of HaloTag7.

While the absence of fluorescence of the spirocyclic form of rhodamines has been exploited for the creation of numerous (fluorogenic) probes,[^3^, ^4^] observation of both the spirocyclic and zwitterionic form would enable a direct readout of the equilibrium of spirocyclization and therefore would open up new ways of creating ratiometric fluorescent probes. Here we introduce **c**olor‐**s**hifting **f**luorophores (CSFs) based on spirocyclization, which exist in a dynamic and environmentally sensitive equilibrium between a green and a red fluorescent form. By coupling these CSFs to ligands or proteins, they enable the design of a new type of ratiometric fluorescent probes for biosensing with large dynamic ranges. Such ratiometric sensors have the advantage that their readout is independent of (fluctuations in) sensor concentration.^[5]^

Our attempts to create CFSs was inspired by previous work on hybrid fluorophores (**1**) consisting of a coumarin and a benzopyrylium moiety (Figure 1 b, S1).^[6]^ The reversible switch between spirocyclic and zwitterionic forms also switches the optical signal between the green color of the coumarin moiety ($\lambda_{abs}^{max}=$ 425 nm, $\lambda_{em}^{max}=$ 475 nm) and the red color of the larger π‐conjugated structure ($\lambda_{abs}^{max}=$ 660 nm, $\lambda_{em}^{max}=$ 710 nm), respectively. However, fluorophore **1** mainly exists in its red zwitterionic form in water (Figure S2), and thus would not allow the generation of CSFs. We therefore attempted to push the equilibrium towards the green spirocyclic state by replacing the carboxyl group of **1** by electron‐deficient amides such as acyl cyanamide (**2**), acyl sulfonamide (**3**) and acyl sulfamide (**4**) (Figure 1 b, S1, S2 and Scheme S1).^[7]^ These amides have been shown to promote reversible spirocyclization in various rhodamines.^[7]^ To evaluate the equilibrium between the zwitterionic and spirocyclic forms of compounds **1**–**4**, we measured their D_50_ value, which is defined as the dielectric constant at which absorbance of the fluorescent zwitterion is decreased by half compared to the highest recorded absorbance value measured in dioxane‐water titrations.^[8]^ It has been shown for rhodamines that compounds with D_50_ values around 50 are candidates for the generation of fluorogenic probes.[^8^, ^9^] The introduction of acyl cyanamide (**2**), acyl sulfonamide (**3**) and acyl sulfamide (**4**) allowed to stepwise increase the D_50_ value (Figure 1 c), pushing the equilibrium of spirocyclization of CSFs towards the green spirocyclic form (Table S1). The environmental sensitivity of the equilibrium of spirocyclization of CSFs was further examined by addition of the surfactant sodium dodecyl sulfate (SDS), which has been shown to shift the equilibrium from the spirocyclic to the zwitterionic form.^[8]^ Incubation of **2**–**4** with SDS caused a decrease of the fluorescence signal in the green channel while increasing that in the red channel, indicating that SDS indeed promotes the opening of the spirocyclic form (Figure S2).

To probe the environmental sensitivity of CSFs towards proteins, we employed HaloTag7, a widely used self‐labeling protein tag,^[10]^ as a model system. Probe **5**–**8** were synthesized by conjugating the chloroalkane HaloTag7 substrate [2‐(2‐((6‐chlorohexyl)oxy)ethoxy)ethan‐1‐amine] with **1**–**4** (Figure 1 d and Scheme S1). Probe **5** served as a reference compound while probes **6**–**8** were expected to be CSF probes. Prior to coupling to HaloTag7, probes **6**–**8** exist predominantly as spirolactams and in the green channel possess similar extinction coefficients and quantum yields as coumarin fluorophores (Figure 1 g, S3 and Table S2).^[11]^ Upon coupling to HaloTag7, the absorbance and fluorescence signal of **6**–**8** largely shift from green to red, indicating a transition from the closed to the open form. In contrast, the spectral properties of reference probe **5** remained invariable as it already existed in the zwitterionic form prior to coupling to HaloTag7 (Figure S3). Notably, probe **8** carrying a N,N‐dimethylsulfonamide moiety showed the largest shift in color upon HaloTag7 labeling (Figure 1 e,f) with 648‐fold and 2392‐fold ratiometric signal changes in absorbance and fluorescence, respectively (Figure 1 g). In vitro kinetic studies of **5**–**8** for HaloTag7 labeling show that the CSF probes react with second order rate constants of 10^3^–10^4^ M^−1^ s^−1^, which is in line with the labeling rates of HaloTag7 substrates with similar structures (Figure S4).^[12]^

The remarkable ability of HaloTag7 to influence the spirocyclization of rhodamines can be exploited to develop biosensors in which an analyte affects the conformation of HaloTag7 and thereby also the equilibrium of spirocyclization. This has recently been demonstrated with the development of a HaloTag7‐based calcium (Ca^2+^) indicator.^[4]^ Here, insertion of a Ca^2+^‐binding domain into the structure of HaloTag7 resulted in chimera in which calcium‐binding affected the equilibrium of spirocyclization of a HaloTag‐bound rhodamine and thereby its fluorescence intensity. We hypothesized that replacement of single‐channel fluorophores by CFSs could offer a ratiometric Ca^2+^ sensor (Figure 2 a). To identify protein chimeras that maximally affect the equilibrium of spirocyclization of CSFs, we generated 37 different sensor designs based on HaloTag7 (Table S3). Of these, 26 chimeras were generated by insertion of Ca^2+^ sensing domains at different locations in the vicinity of the HaloTag7 fluorophore binding site, expecting the conformational change upon Ca^2+^ binding to affect the equilibrium between the zwitterionic and spirocyclic forms. These Ca^2+^ sensing domains consisted either of a fusion between a calmodulin domain and its cognate M13 peptide (i.e. CaM‐M13) or a troponin domain, both known to undergo large conformational change upon Ca^2+^ binding.^[13]^ Additional 11 chimeras were generated by circular permutation of HaloTag7, which resulted in new N and C termini close to the fluorophore binding site. To these termini were then fused M13 and CaM, respectively. Screening these 37 engineered HaloTag7 variants for calcium sensitivity indicated that the insertion of the CaM/M13 domain between residues Gln150 and Ala151 of HaloTag7 led to the highest fluorescence ratio change upon calcium binding (Figure 2 b and Table S3). This construct, named rHCaMP, displayed, after labeling with CSF **8**, Ca^2+^ responsive green ($\lambda_{abs}^{max}=$ 428 nm, $\lambda_{em}^{max}=$ 472 nm) and red fluorescence ($\lambda_{abs}^{max}=$ 667 nm, $\lambda_{em}^{max}=$ 692 nm) (Figure S5, S6). Sensor titration with Ca^2+^ showed a 14.1‐fold ratiometric fluorescence signal change with a c_50_ value of 363±14 nM for Ca^2+^ (Figure 2 c and Table S2), which represents the Ca^2+^ concentration at which the sensor displays half of its maximum ratio change. To test the potential of the approach for live‐cell imaging, rHCaMP was stably expressed in a U2OS cell line. Cells were incubated with CSF **8** and subsequently treated with ionomycin to increase the intracellular calcium concentration.^[14]^ Live‐cell microscopy showed a slight fluorescence increase in the green channel and a large decrease in the red channel over time, with 3.9‐fold fluorescence ratio change (Figure 2 d,e, S7). These proof‐of‐principle experiments highlight the potential of CSF to create novel ratiometric biosensors.

**Figure 2:**
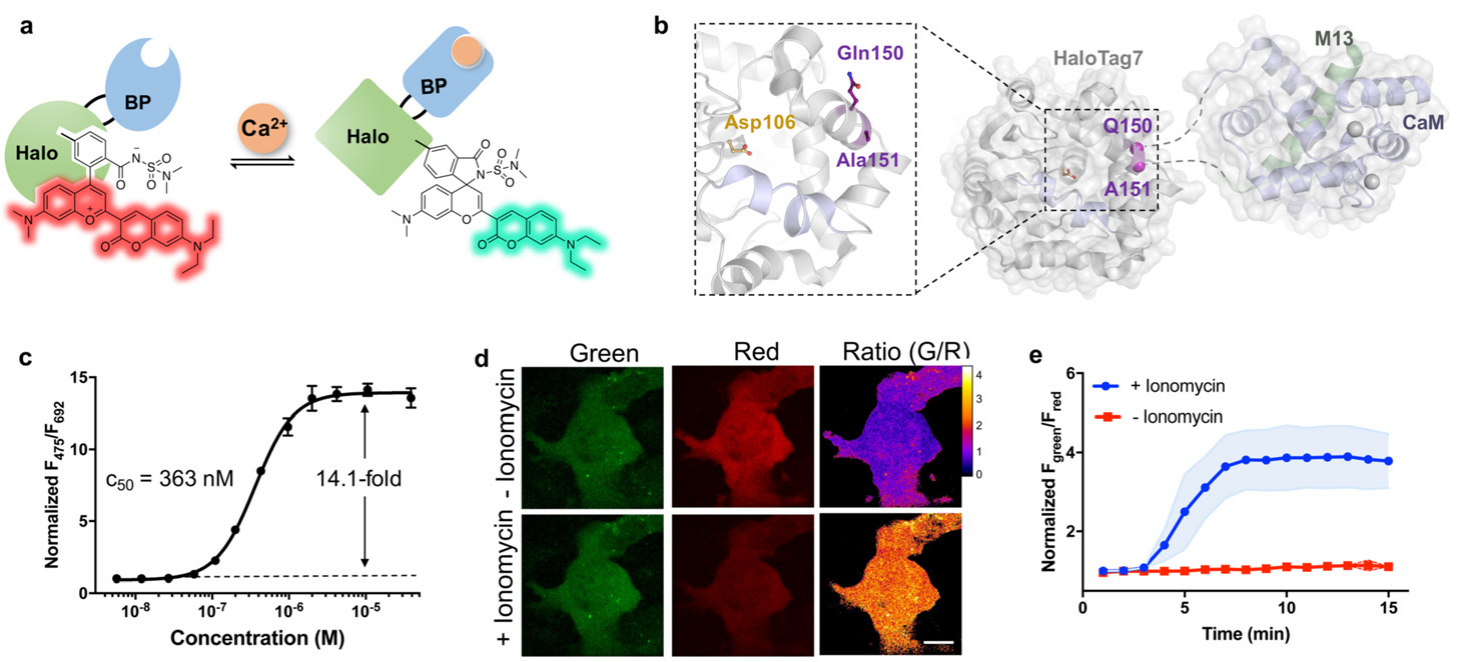
CSF‐based Ca^2+^ biosensor. a) Cartoon of CSF‐based semisynthetic Ca^2+^ biosensor. Ca^2+^ binding induces a conformational change, thereby shifting the equilibrium between red and green fluorescent forms. b) Schematic representation of rHCaMP. The HaloTag7 crystal structure (4kaj) is represented as grey cartoon. The CaM‐M13 domains were extracted from the GCaMP2 crystal structure (3evr) and are represented as blue (CaM) and green (M13) cartoons. Both HaloTag7 and CaM/M13 were positioned to fuse of CaM/M13 to rHCaMP via Q150 and A151 of HaloTag7 (α‐carbons represented as purple spheres). c) rHCaMP‐**8** sensor titration with Ca^2+^. d) Live‐cell Ca^2+^ imaging with rHCaMP‐**8**. U2OS cells expressing rHCAMP **8** and treated with ionomycin (1 μM) prior to imaging. Scale bar: 20 μm. e) Time course analysis of fluorescence ratio F_green_/F_red_ upon ionomycin treatment. The ratio was normalized to the value at 0 min. s.d. is represented as shade area. N=20 cells from 5 independent experiments.

Considering the large number of existing fluorogenic probes,[^1b^, ^15^] we hypothesized that the binding of CSF‐based probes to other proteins could also switch the equilibrium of spirocyclization. We therefore coupled CSF **2**–**4** to ligands of the following protein targets (Figure 3 a, S8–S11): (1) SNAP‐tag^[16]^ and bacterial dihydrofolate reductase (eDHFR),^[17]^ two proteins used as tags in protein labeling; (2) human carbonic anhydrase II (HCAII), which plays a key role in maintaining acid‐base balance in biological systems;^[18]^ and (3) human sepiapterin reductase (hSPR), which participates in the biosynthesis of the cofactor tetrahydrobiopterin and is an off‐target of sulfa drugs.^[19]^ For CSFs **9**–**12**, different electron‐deficient amides were empirically selected, reflecting the different abilities of the corresponding proteins for shifting the equilibrium towards the zwitterionic form. For all proteins, binding of CSFs **9**–**12** shifted the equilibrium from the green towards the reds form (Figure 3 b–f and S8–S11). The most pronounced change was seen for the probe **9** for eDHFR, which showed a 2340‐fold fluorescence intensity ratio change upon eDHFR binding (Figure 3 b,g). These ratiometric probes might be used in microscopy or as specific probes to quantify the presence of their protein target in complex biological samples. Motivated by the large dynamic range of probe **9** upon binding to eDHFR, we created a bioassay for the reduced form of the cofactor nicotinamide adenine dinucleotide phosphate (NADPH) (Figure 3 h). For this, we took advantage of a previously described mutant of eDHFR (Figure S8),^[20]^ in which drug binding is strictly dependent on the presence of the NADPH (Figure 3 h). Titration of the eDHFR mutant with NADPH in the presence of probe **9** resulted in a maximum fluorescence ratio change of 486‐fold with a c_50_ for NADPH of 140±21 nM (Figure 3 i,j, S8). This simple ratiometric and highly sensitive assay should find applications in the quantification of NADPH in biological samples as well as in bioassays.

**Figure 3:**
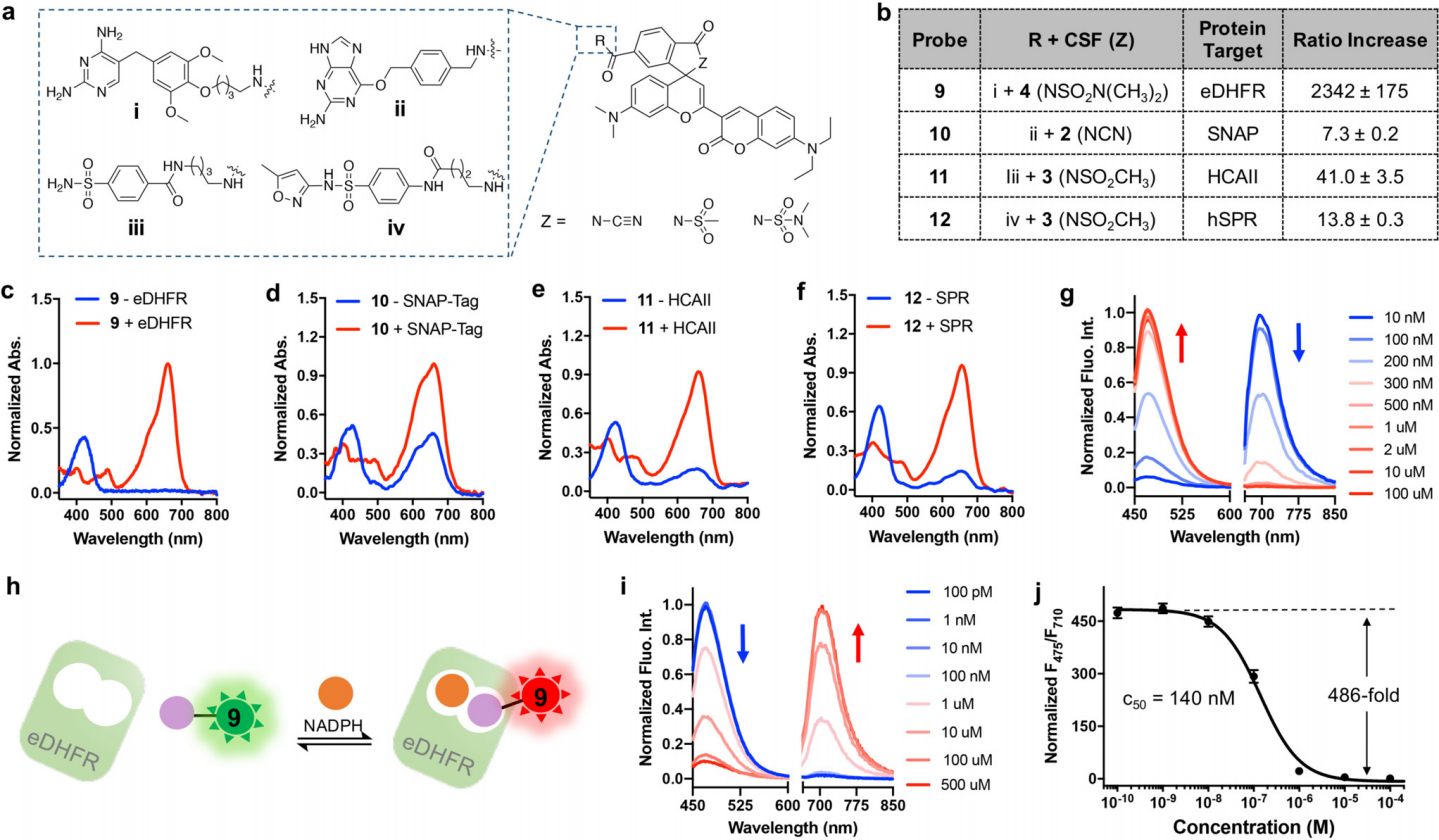
Probes for proteins and metabolites based on CSFs. a) General structure of CSF‐based probes for eDHFR (i; based on trimethoprim); SNAP‐tag (ii; based on benzylguanine); HCAII (iii; based on benzenesulfonamide); hSPR (iv; based on sulfamethoxazole). b) CSF‐based probes **9**–**12** and fluorescence ratio change (F_707_/F_475_) induced upon binding to target proteins. c–f) Normalized absorbance of CSF **9** (c), **10** (d), **11** (e), **12** (f) in absence and presence of protein target. g) Normalized fluorescence emission spectra of **9** in the presence of eDHFR and various concentrations of the competitor methotrexate (MTX). h) A CSF‐based ratiometric sensor for NADPH. Binding of **9** to the eDHFR mutant is strictly dependent on NADPH, offering a ratiometric fluorescent readout. i) Normalized emission spectra of **9** in presence of eDHFR mutant and varying concentrations of NADPH. j) Normalized fluorescence ratio of **9** in presence of eDHFR mutant and varying concentrations of NADPH. Error bars show±s.d. from triplicate experiments.

In summary, we report novel environmentally sensitive fluorophores that reversibly switch between a green fluorescent spirocyclic form and red fluorescent zwitterionic form. Coupled to an engineered fusion of HaloTag7 with a Ca^2+^ sensing domain (rHCaMP), these CSF allowed for fluorescence ratiometric measurement of Ca^2+^ in vitro and in cellulo. CSFs coupled to different protein ligands resulted in probes with up to 2400‐fold fluorescence ratio changes upon target binding. CSFs thus represent a new platform for the generation of ratiometric fluorescent probes, biosensors and bioassays.

## Conflict of interest

K.J. and L.W. are inventors on a patent filed by the Max Planck Society.

## Supporting information

As a service to our authors and readers, this journal provides supporting information supplied by the authors. Such materials are peer reviewed and may be re‐organized for online delivery, but are not copy‐edited or typeset. Technical support issues arising from supporting information (other than missing files) should be addressed to the authors.

Supplementary
