# Supplementary material for: Environmentally Sensitive Color‐Shifting Fluorophores for Bioimaging
Source: Angew Chem Int Ed Engl. 2020 Sep 28;59(49):21880–4. doi: 10.1002/anie.202008357 (PMC7756609; doi:10.1002/anie.202008357)
Supplement: Supplementary file 1 — Supplementary [file ANIE-59-21880-s001.pdf]

Angewandte Chemie
Eine Zeitschrift der Gesellschaft Deutscher Chemiker

Supporting Information

Environmentally Sensitive Color-Shifting Fluorophores for Bioimaging

Lu Wang[+], Julien Hiblot[+], Christoph Popp, Lin Xue, and Kai Johnsson**

anie_202008357_sm_miscellaneous_information.pdf

Table of content

1. Supplemental Figures and Tables 3-11
2. Protein Sequence 12-14
3. Chemical synthesis and characterization 14-23
4. Methods for *in vitro* tests 23–27
5. Methods for cell experiments 27-28
6. methods for data analysis 29
7. References 29
8. Supplementary NMR spectra 30
9. Supplementary HRMS spectra 41

Supplemental Figures and Tables

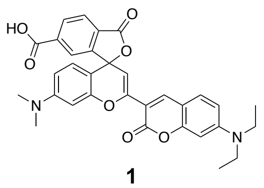

1

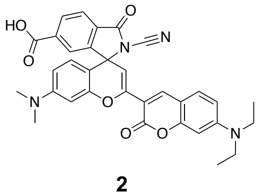

2

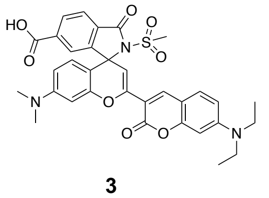

3

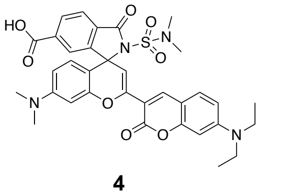

4

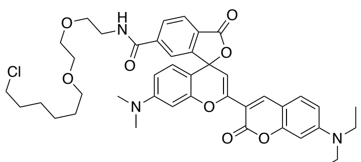

5

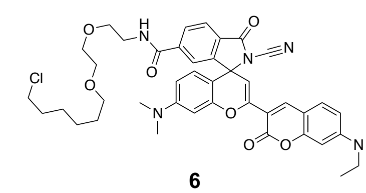

6

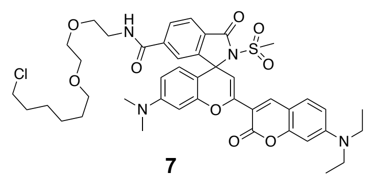

7

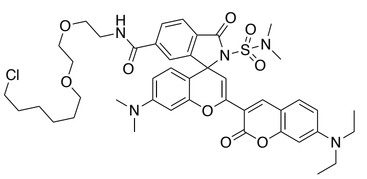

8

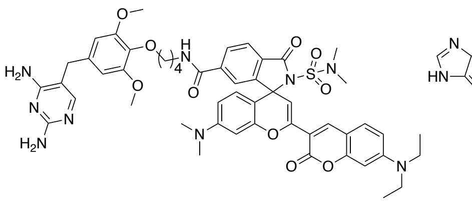

9

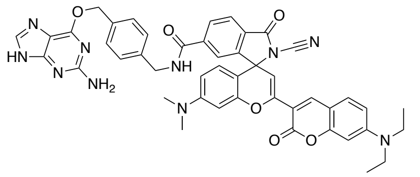

10

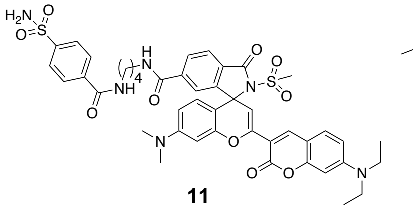

11

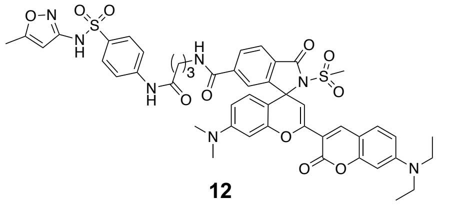

12

Figure S1. Structures of color-shifting fluorophores and probes described in this work.

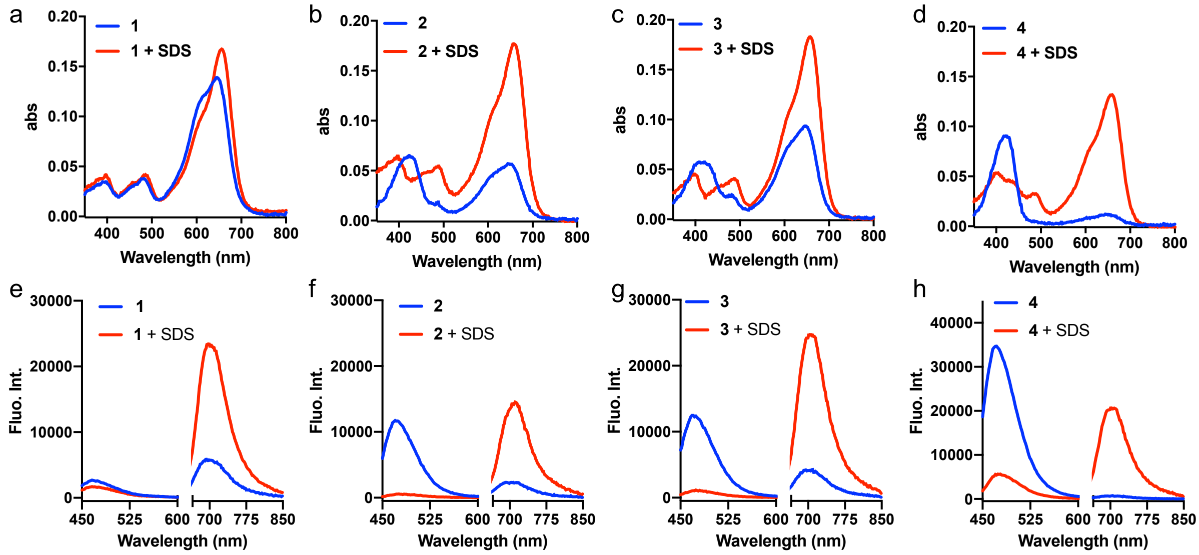

Figure S2. Spectroscopic properties of the color-shifting fluorophores (CSFs) **1-4**. Absorption (**a-d**) and fluorescence emission (**e-h**) spectra of **1** (**a**, **e**), **2** (**b**, **f**), **3** (**c**, **g**) and **4** (**d**, **h**) measured in presence (red line) and absence (blue line) of sodium dodecyl sulfate (SDS, 0.1% w/v) after 30 min incubation. Probe **1-4**: 2 μM.

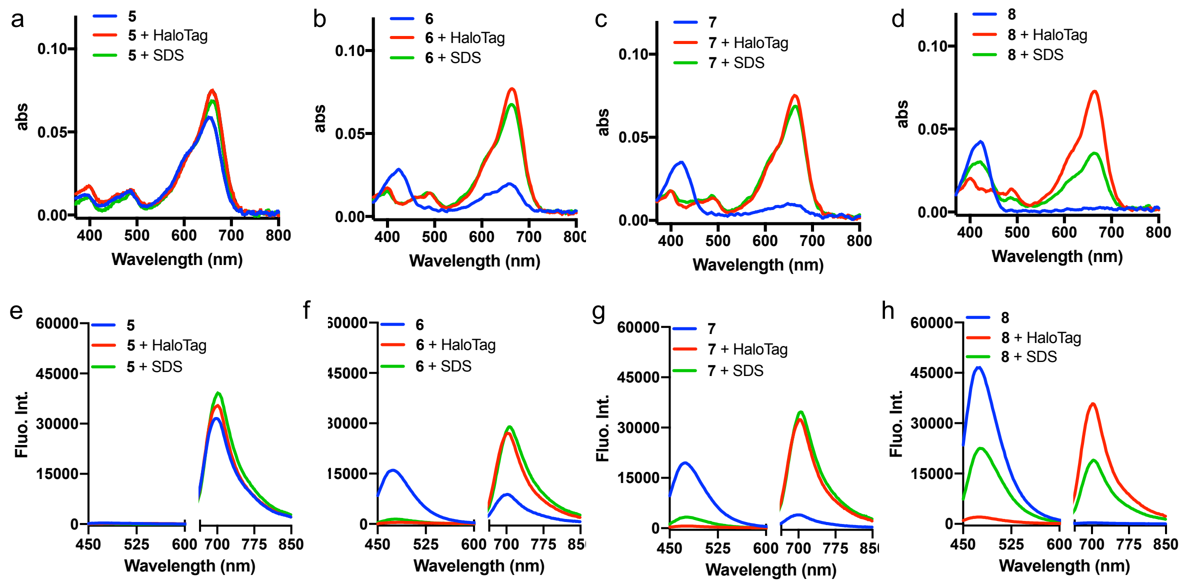

Figure S3. Spectroscopic properties of the HaloTag7 CSFs substrates. Absorption (**a-d**) and fluorescence emission (**e-h**) spectra of **5** (**a**, **e**), **6** (**b**, **f**), **7** (**c**, **g**), **8** (**d**, **h**) measured in the absence (blue line) and presence of HaloTag7 (red line) or in presence of SDS (0.1% w/v, green line). Probe **5-8**: 1 μM, HaloTag7: 5 μM. 1 h incubation.

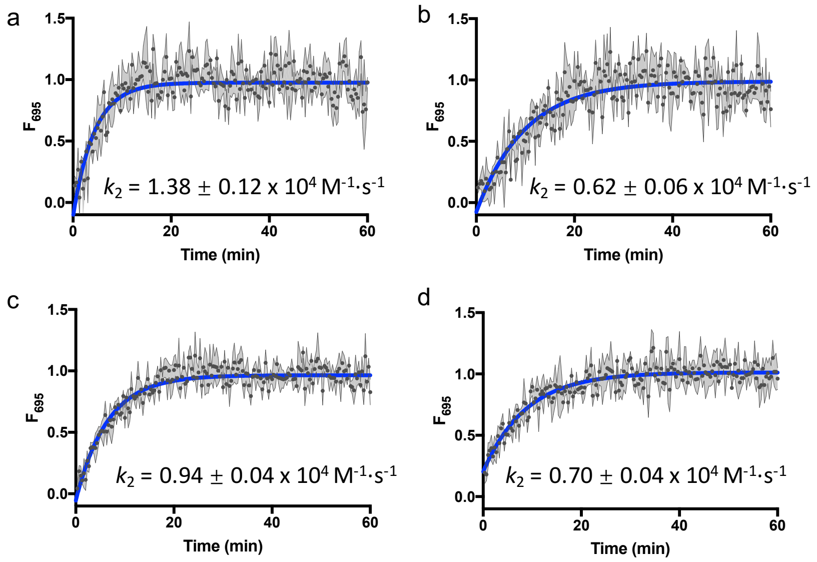

Figure S4. Labeling Kinetics of HaloTag7 with probe **5** (**a**), **6** (**b**), **7** (**c**) and **8** (**d**). Normalized fluorescence intensity at 695 nm in 60 minutes. The corresponding second order rate constants k_2 are annotated in each graph. Probe **5-8**: 50 nM, HaloTag7: 250 nM. Error bars show ± s.d. from triplicate experiment.

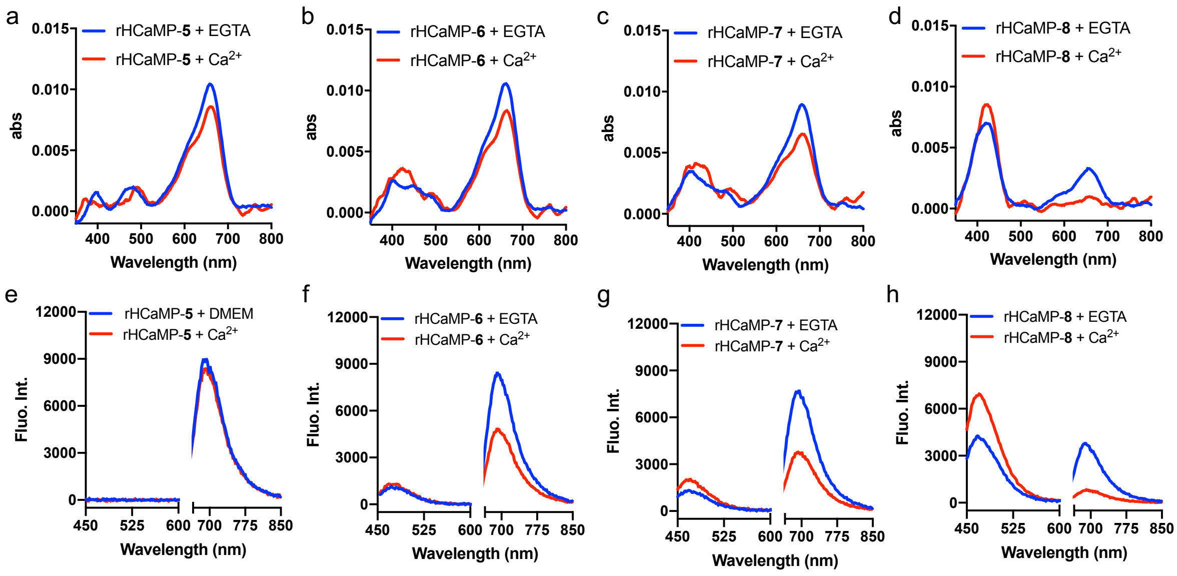

Figure S5. rHCaMP-**8** Ca^{2+} sensor *in vitro* characterization. Absorption (**a-d**) and fluorescence emission (**e-h**) spectra of rHCaMP labeled with **5** (**a**, **e**), **6** (**b**, **f**), **7** (**c**, **g**), **8** (**d**, **h**) measured in the presence of EGTA (1mM, blue line) or Ca^{2+} (1 mM, red line) after 30 min incubation. rHCaMP: 2.5 μM, Probe **8**: 250 nM.

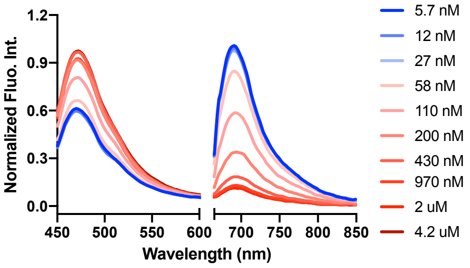

Figure S6. Fluorescence emission spectra corresponding to the titration of rHCaMP-**8** CSFs-based biosensor in presence of various Ca^{2+} concentration. rHCaMP: 1.25 µM, Probe **8**: 250 nM.

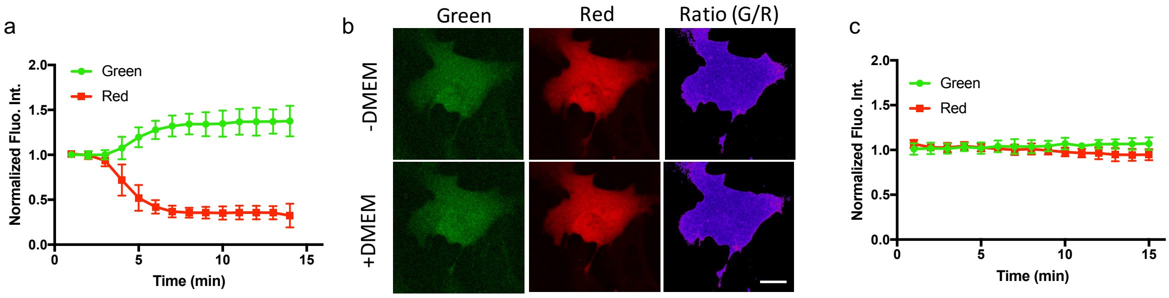

Figure S7. rHCaMP-**8** Ca^{2+} sensor characterization *in cellulo*. (**a**) Time course of the fluorescence signal in both green and red channels of rHCaMP-**8** in U2OS upon addition of ionomycin (1 µM). The fluorescence intensities were normalized to the values at time 0. (**b**) Live rHCaMP-**8** expressing U2OS cells imaged by fluorescence microscopy. Control experiment upon DMEM addition instead of ionomycin. (**c**) Corresponding time course of the fluorescence signal in both green and red channels of rHCaMP-**8** in U2OS upon addition of control DMEM. 20 cells were analyzed from five independent experiments. Scale bar: 20 µm.

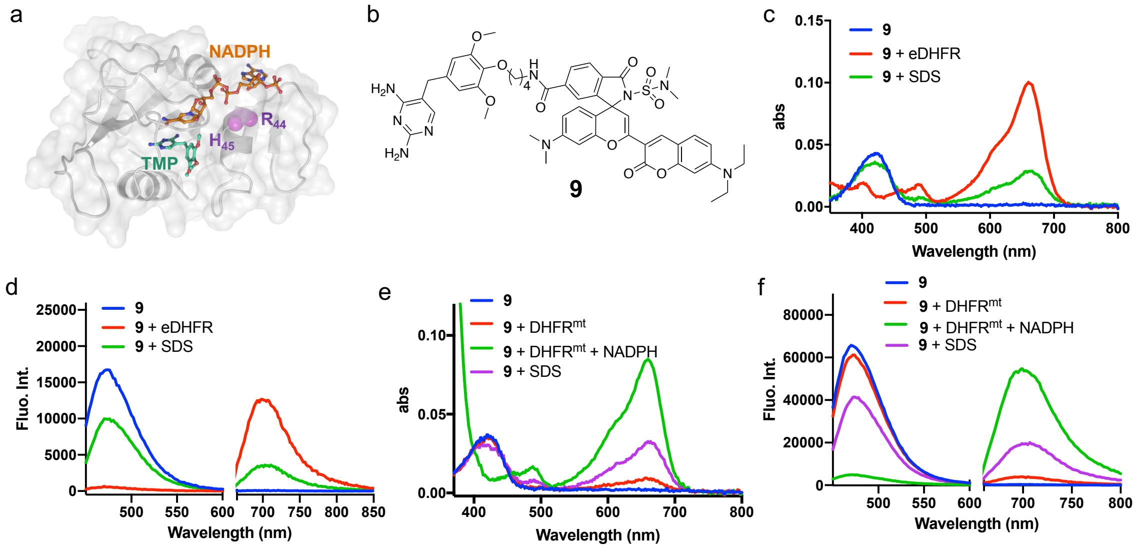

Figure S8. Spectroscopic properties of probe **9** upon binding to eDHFR and eDHFR mutant. (**a**) Crystal structure of eDHFR bound to NADPH and TMP. The structure was extracted from GFP-LAMA-F98 (6rul) and is represented as grey cartoon while NADPH and TMP are represented as orange and green sticks, respectively. (**b**) Chemical structure of the CSF **9**. (**c, d**) Absorption (**c**) and fluorescence emission (**d**) spectra of **9** (1 µM) measured free in buffer (blue line), in presence of eDHFR (2 µM) and NADPH (10 µM) (red line) or in presence of SDS (0.1% w/v, green line) after 30 min. (**e**, **f**) Absorption (**e**) and fluorescence emission (**f**) spectra of probe **9** (1 µM) measured free in buffer (blue line), in presence of eDHFR mutant (R44L and H45Q, 2 µM, red line), of eDHFR mutant (2 µM) and NADPH (10 µM) (green line), or of SDS (0.1% w/v, purple line) after 30 min incubation.

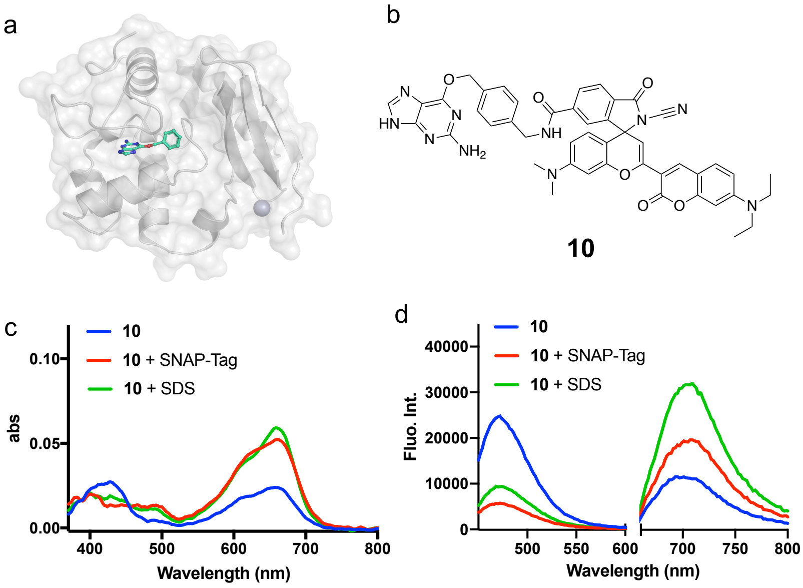

Figure S9. Spectroscopic properties of probe **10** upon binding to SNAP-tag. (**a**) Crystal structure of SNAP-tag (3kzz) represented as grey cartoon bound to O6-benzylguanine represented as green sticks. (**b**) Chemical structure of **10.** (**c**, **d**) Absorption (**c**) and fluorescence emission (**d**) spectra of **10** measured in the absence (blue line) and presence of SNAP-tag (red line) or in presence of SDS (0.1% w/v, green line). Probe **10**: 1 μM, SNAP-tag: 5 μM. 1 h incubation.

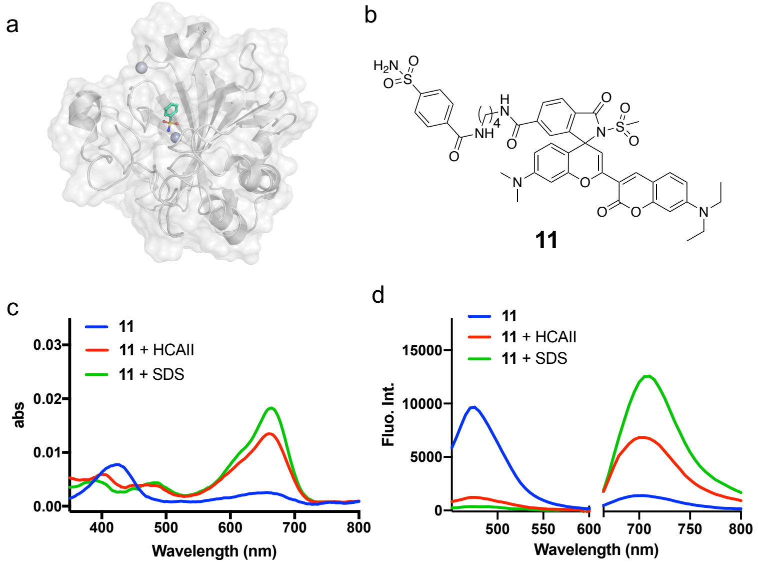

Figure S10. Spectroscopic properties of CSF **11** upon binding to HCAII. (**a**) Crystal structure of HCAII (6gdc) represented as grey cartoons bound its benzenesulfonamide (BSA) inhibitor represented as green sticks. The structural cations atoms are represented as spheres. (**b**) Chemical structure of **11**. (**c**, **d**) Absorption (**c**) and fluorescence emission (**d**) spectra of compound **11** (250 nM) measured free in buffer (blue line), in presence of HCAII (10 μM, red line) or of SDS (0.1% w/v, green line) after 30 min incubation.

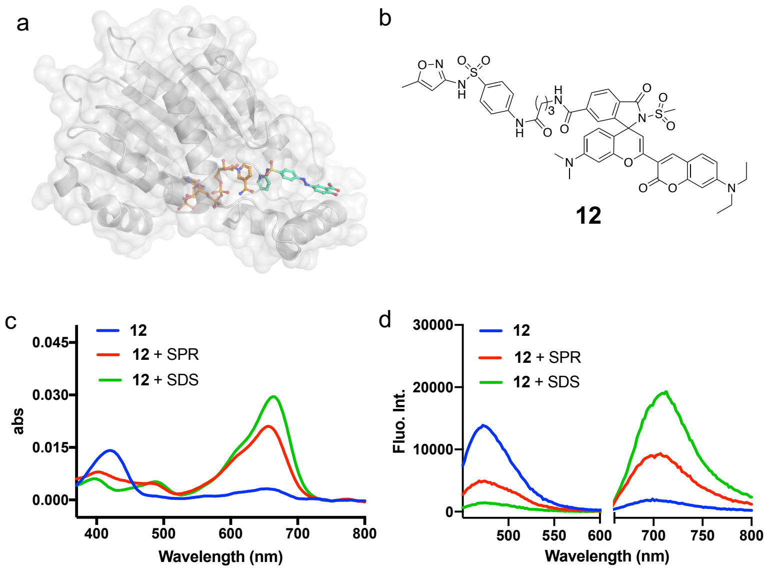

Figure S11. Spectroscopic properties of CSF **12** upon binding to hSPR. (**a**) Crystal structure of hSPR (4j7x) represented as grey cartoons bound $NADP^+$ and sulfasalazine represented as orange and green sticks, respectively. (**b**) Chemical structure of **12**. (**c**, **d**) Absorption (**c**) and fluorescence emission (**d**) spectra of compound **12** (500 nM) measured free in buffer (blue line), in presence of hSPR (2 µM) and $NADP^+$ (100 µM, red line), or of SDS (0.1%, green line) after 30 min incubation.

Supplementary Table 1. Photophysical properties of color-shifting fluorophores **1-4**.

probe	Z	λ_{ex} (green/red)	ε($M^{-1}cm^{-1}$) (green/red)	λ_{em} (green/red)	Φ (green/red)	D_{50}
**1**	OH	-[a]/658[b]	-[a]/83000[b]	468[a]/700[b]	-[a]/0.14[b]	24[c]
**2**	NCN	423[a]/660[b]	32000[a]/88000[b]	471[a]/710[b]	0.09[a]/0.12[b]	51[c]
**3**	NSCH3	425[a]/658[b]	25000[a]/91000[b]	470[a]/706[b]	0.07[a]/0.13[b]	62[c]
**4**	NSNMe2	423[a]/660[b]	45000[a]/66000[b]	471[a]/706[b]	0.09[a]/0.15[b]	>70[c]

[a] absorbance, emission and quantum yield of the spricyclic, green fluorescent CSFs were obtained in HEPES buffer with 0.1% Triton X-100. [b] absorbance, emission and quantum yield of the zwitterionic, red fluorescent CSFs in HEPES buffer with 0.1% SDS. [c] dioxane-H_2O mixture (v/v: 90/10 –10/90). The relatively low quantum yields of **2-4** in their spirocyclized form and of the coumarin derivative **S2** in aqueous solution are probably due to formation of twisted internal charge transfer (TICT) states.[13,14]

Supplementary Table 2. Photophysical properties of CSFs-based probes **5-12**.

probe	target	Z	λ_{ex} (green/red)	ε($M^{-1}cm^{-1}$) (green/red)	λ_{em} (green/red)	Φ (green/red)	Ratio
**5**	Halo	OH	-^{a}/665^{b}	-^{a}/72000^{b}	-^{a}/705^{b}	-^{a}/0.12^{b}	2.9 ± 0.8^{c}
**6**	Halo	NCN	426^{a}/665^{b}	28000^{a}/75000^{b}	476^{a}/706^{b}	0.1^{a}/0/1^{b}	116.5 ± 5.8^{c}
**7**	Halo	NSO_2CH_3	428^{a}/666^{b}	33000^{a}/76000^{b}	477^{a}/707^{b}	0.15^{a}/0.11^{b}	254.4 ± 4.2^{c}
**8**	Halo	$NSO_2N(CH_3)_2$	428^{a}/667^{b}	41000^{a}/72000^{b}	476^{a}/706^{b}	0.1^{a}/0.13^{b}	2391.9 ± 34^{c}
**8**	Halo-CaM	$NSO_2N(CH_3)_2$	428^{d}/667^{e}	40000^{d}/12000^{e}	472^{d}/692^{e}	0.09^{a}/0.2^{e}	14.1 ± 0.4^{f}
**9**	eDHFR	$NSO_2N(CH_3)_2$	426^{a}/660^{g}	42000^{a}/98000^{g}	478^{a}/704^{g}	-	2342 ± 175^{h}
**10**	SNAP	NCN	428^{a}/661^{i}	26000^{a}/53000^{i}	471^{a}/706^{i}	-	7.3 ± 0.2^{j}
**11**	HCAII	NSO_2CH_3	430^{a}/660^{k}	34000^{a}/58000^{k}	476^{a}/706^{k}	-	41.0 ± 3.5^{l}
**9**	eDHFR^{mt}	$NSO_2N(CH_3)_2$	427^{a}/664^{m}	34000^{a}/83000^{m}	478^{a}/703^{m}	-	2321.8 ± 176.1^{n}
**12**	SPR	NSO_2CH_3	430^{a}/662°	28000^{a}/46000°	476^{a}/704°	-	13.8 ± 0.3^{p}

^{a} absorbance, emission and quantum yield in green channel in the absence of respective reporter proteins. ^{b,d,e,g,i,k,m} absorbance, emission and quantum yield in red channel in the presence of HaloTag7 (^{b}), rHCaMP with Ca^{2+} (^{d}), rHCaMP with EGTA (^{e}), eDHFR (^{g}), or SNAP-tag (^{i}), HCAII (^{k}), eDHFR mutant with NADPH (^{m}), or hSPR with $NADP^{+}$ (^{o}). ^{c,f,j,l,n,p} enhancement of fluorescence ratio (intensity at 707 nm/ intensity at 477 nm) after addition of HaloTag7 (^{c}), rHCaMP with Ca^{2+}(^{f}), SNAP (^{j}), HCAII (^{l}), eDHFR mutant with NADPH (^{n}), or hSPR with $NADP^{+}$ (^{p}). ^{h} enhancement of fluorescence ratio (intensity at 707 nm/ intensity at 477 nm) upon addition of methotrexate (MTX). $^{a-e,g-p}$ HEPES buffer (pH 7.3) with 0.1% Triton X-100, ^{f} Calcium Calibration Buffer. Error bars show ± s.d. from triplicate experiments.

Supplementary Table 3. List of HaloTag7-based Ca^{2+} sensor designs.

	Protein name	Last AA	Linker N	Ca sensing domain	Linker C	First AA	R_{EGTA}/R_{Ca2+}
**Native HaloTag7**	Halo[133-CaM/M13-134]	Arg133	None	CaM/M13	None	Pro134	0.49
	Halo[133-CaM/M13-137]	Arg133				Thr137	0.88
	Halo[134-CaM/M13-135]	Pro134				Ile135	0.82
	Halo[134-CaM/M13-136]	Pro134				Pro136	1.1
	Halo[135-CaM/M13-136]	Ile135				Pro136	0.74
	Halo[136-CaM/M13-137]	Pro136				Thr137	0.56
	Halo[137-CaM/M13-138]*	Thr137				Trp138	0.98
	Halo[138-CaM/M13-139]*	Trp138				Asp139	1.1
	Halo[139-CaM/M13-140]*	Asp139				Glu140	1.2
	Halo[142-CaM/M13-143]*	Pro142				Glu143	0.96
	Halo[143-CaM/M13-144]*	Glu143				Phe144	0.97
	Halo[146-CaM/M13-147]*	Arg146				Glu147	1.1
	Halo[146-CaM/M13-148]*	Arg146				Thr148	1.3
	Halo[147-CaM/M13-148]*	Glu147				Thr148	1.9
	**Halo[150-CaM/M13-151]***	Gln150				Ala151	**5.5**
	Halo[153-CaM/M13-154]*	Arg153				Thr154	0.89
	Halo[178-CaM/M13-179]	Val178				Arg179	0.97
	Halo[243-CaM/M13-244]	Pro143				Gly244	0.94
	Halo[133-S_Tw_S-137]	Arg133	VADA	MiniTopoC	PIYP	Thr137	0.56
	Halo[133-Tw-137]	Arg133	None		None	Thr137	3.6
	Halo[134-S_Tw_S-135]	Pro134	VADA		PIYP	Ile135	1.26
	Halo[134-Tw-135]	Pro134	None		None	Ile135	1.24
	Halo[178-S_Tw_S-179]	Val178	VADA		PIYP	Arg179	0.70
	Halo[178-Tw-179]	Val178	None		None	Arg179	0.76
	Halo[243-S_Tw_S-244]	Pro143	VADA		PIYP	Gly244	0.89
	Halo[243-Tw-244]	Pro143	None		None	Gly244	0.94
**Circular permuted HaloTag7#**	cpHalo_133-137_GGS	Thr137	GGT	M13/cpHalo/CaM	GGS	Arg133	1.12
	cpHalo_133-137_GGSx2	Thr137	GGTGGS		GGSGGS	Arg133	0.51
	cpHalo_134	Ile135	None		None	Pro134	0.65
	cpHalo_134_GGS	Ile135	GGT		GGS	Pro134	0.66
	cpHalo_134_GGSx2	Ile135	GGTGGS		GGSGGS	Pro134	0.72
	cpHalo_135-137	Thr137	GGT		GGS	Ile135	0.90
	cpHalo_135-137_GGS	Thr137	GGTGGS		GGSGGS	Ile135	0.42
	cpHalo_136	Thr137	None		None	Pro136	0.70
	cpHalo_136_GGS	Thr137	GGT		GGS	Pro136	0.50
	cpHalo_141-147	Glu147	None		None	Trp141	3.5
	cpHalo_141-147_GGS	Glu147	GGT		GGS	Trp141	1.2

# HaloTag7 circular permutation was performed linking the original N and C-termini by the $(GGS/T)_{x5}$ linker such as: Ile295-GGTGGSGGTGGSGGS-Ile4.

* The CaM/M13 inserted proteins present a $(GGS/T)_{x2}$ linker between CaM and M13 (GGSGGT).

In bold is indicated the design chosen for rHCaMP.

Protein sequences

>SNAP-HaloTag7

MASWSHPQFEKGADDDDKVPHMDKDCEMKRTTLDSPLGKLELSGCEQGLHEIIFL
GKGTSAADAVEVPAPAAVLGGPEPLMQATAWLNAYFHQPEAIEEFPVPALHHPVFQ
QESFTRQVLWKLLKVVKFGEVISYSHLAALAGNPAATAAVKTALSGNPVPILIPCHRV
VQGDLDVGGYEGGLAVKEWLLAHEGHRLGKPGLGEFGGSGGSGGSGGSEIGTGF
PFDPHYVEVLGERMHYVDVGPRDGTPVLFLHGNPTSSYVWRNIIPHVAPTHRCIAP
DLIGMGKSDKPDLGYFFDDHVRFMDAFIEALGLEEVVLVIHDWGSALGFHWAKRNP
ERVKGIAFMEFIRPIPTWDEWPEFARETFQAFRTTDVGRKLIIDQNVFIEGTLPMGVV
RPLTEVEMDHYREPFLNPVDREPLWRFPNELPIAGEPANIVALVEEYMDWLHQSPV
PKLLFWGTPGVLIPPAEAARLAKSLPNCKAVDIGPGLNLLQEDNPDLIGSEIARWLST
LEISGAPGFSSISAHHHHHHHHHH

Strep-tag / enterokinase cleavage site / SNAP-Tag / linker / HaloTag7 / His$_{x10}$tag

>rHCaMP

MASWSHPQFEKGADDDDKVPHGSEIGTGFPFDPHYVEVLGERMHYVDVGPRDGT
PVLFLHGNPTSSYVWRNIIPHVAPTHRCIAPDLIGMGKSDKPDLGYFFDDHVRFMDA
FIEALGLEEVVLVIHDWGSALGFHWAKRNPERVKGIAFMEFIRPIPTWDEWPEFARE
TFQLPDQLTEEQIAEFKEEFSLFDKDGDGTITTKELGTVMRSLGQNPTEAELQDMIN
EVDADGDGTIDFPEFLTMMARKMKYRDTEEEIREAFGVFDKDGNGYISAAELRHVM
TNLGEKLTDEEVDEMIREADIDGDGQVNYEEFVQMMTAKGGTGGSMVDSSRRKW
NKTGHAVRAIGRLSSLEAFRTTDVGRKLIIDQNVFIEGTLPMGVVRPLTEVEMDHYR
EPFLNPVDREPLWRFPNELPIAGEPANIVALVEEYMDWLHQSPVPKLLFWGTPGVLI
PPAEAARLAKSLPNCKAVDIGPGLNLLQEDNPDLIGSEIARWLSTLEISGAPGFSSIS
AHHHHHHHHHH

Strep-tag / enterokinase cleavage site / HaloTag7 / Calmodulin / linker / M13 / His$_{x10}$tag

>eDHFR-SNAP

MASWSHPQFEKGADDDDKVPHMDKDCEMKRTTLDSPLGKLELSGCEQGLHEIIFL
GKGTSAADAVEVPAPAAVLGGPEPLMQATAWLNAYFHQPEAIEEFPVPALHHPVFQ
QESFTRQVLWKLLKVVKFGEVISYSHLAALAGNPAATAAVKTALSGNPVPILIPCHRV
VQGDLDVGGYEGGLAVKEWLLAHEGHRLGKPGLGEFGGSGGSGGSGGSMISLIA
ALAVDRVIGMENAMPWNLPADLAWFKRNTLNKPVIMGRHTWESIGRPLPGRKNIILS

SQPGTDDRVTWVKSVDEAIAACGDVPEIMVIGGGRVYEQFLPKAQKLYLTHIDAEVE
GDTHFPDYEPDDWESVFSEFHDADAQNSHSYCFEILERRGAPGFSSISAHHHHHH
HHHH

Strep-tag / enterokinase cleavage site / SNAP-Tag / linker / DHFR / His$_{x10}$tag

>HCAII

MASWSHPQFEKGADDDDKVPHMDKDCEMKRTTLDSPLGKLELSGCEQGLHEIIFL
GKGTSAADAVEVPAPAAVLGGPEPLMQATAWLNAYFHQPEAIEEFPVPALHHPVFQ
QESFTRQVLWKLLKVVKFGEVISYSHLAALAGNPAATAAVKTALSGNPVPILIPCHRV
VQGDLDVGGYEGGLAVKEWLLAHEGHRLGKPGLGGRLEVLFQGPKAFLEMSHHW
GYGKHNGPEHWHKDFPIAKGERQSPVDIDTHTAKYDPSLKPLSVSYDQATSLRILN
NGHAFNVEFDDSQDKAVLKGGPLDGTYRLIQFHFHWGSLDGQGSEHTVDKKKYAA
ELHLVHWNTKYGDFGKAVQQPDGLAVLGIFLKVGSAKPGLQKVVDVLDSIKTKGKS
ADFTNFDPRGLLPESLDYWTYPGSLTTPPLLECVTWIVLKEPISVSSEQVLKFRKLNF
NGEGEPEELMVDNWRPAQPLKNRQIKASFKGAPGFSSISAHHHHHHHHHH

Strep-tag / enterokinase cleavage site / SNAP-Tag / linker / HCAII / His$_{x10}$tag

>eDHFR^{mt}-SNAP

MASWSHPQFEKGADDDDKVPHMDKDCEMKRTTLDSPLGKLELSGCEQGLHEIIFL
GKGTSAADAVEVPAPAAVLGGPEPLMQATAWLNAYFHQPEAIEEFPVPALHHPVFQ
QESFTRQVLWKLLKVVKFGEVISYSHLAALAGNPAATAAVKTALSGNPVPILIPCHRV
VQGDLDVGGYEGGLAVKEWLLAHEGHRLGKPGLGEFGGSGGSGGSGGSMISLIA
ALAVDRVIGMENAMPWNLPADLAWFKRNTLNKPVIMGLQTWESIGRPLPGRKNIILS
SQPGTDDRVTWVKSVDEAIAACGDVPEIMVIGGGRVYEQFLPKAQKLYLTHIDAEVE
GDTHFPDYEPDDWESVFSEFHDADAQNSHSYCFEILERRGAPGFSSISAHHHHHH
HHHH

Strep-tag / enterokinase cleavage site / SNAP-Tag / linker / DHFR / His$_{x10}$tag

>hSPR

MHHHHHHHHHHENLYFQ|GMEGGLGRAVCLLTGASRGFGRTLAPLLASLLSPGSVL
VLSARNDEALRQLEAELGAERSGLRVVRVPADLGAEAGLQQLLGALRELPRPKGLQ
RLLLINNAGSLGDVSKGFVDLSDSTQVNNYWALNLTSMLCLTSSVLKAFPDSPGLN
RTVVNISSLCALQPFKGWALYCAGKAARDMLFQVLALEEPNVRVLNYAPGPLDTDM

QQLARETSVDPDMRKGLQELKAKGKLVDCKVSAQKLLSLLEKDEFKSGAHVDFYD
K

His_{x10}tag – TEV cleavage site – hSPR

General Experimental Information for Synthesis

General information:

All chemical reagents and dry solvents for synthesis were purchased from commercial suppliers (Sigma-Aldrich, TCI, Alfa Aesar) and were used without further purification or distillation. The composition of mixed solvents is given by the volume ratio (v/v). Reactions were monitored by thin layer chromatography (TLC) performed on TLC-aluminum sheets (Silica gel 60 F_{254}, Merck) or liquid chromatograph-mass spectrometry (LCMS-2020, Shimadzu). Analytical reversed phase LC-MS was equipped with an SPD-20AV UV-VIS photodiode array detector for product visualization on a C18 1.9 µm, 2.1 x 50 mm column (Supelco). Solvent A: 0.1% v/v HCOOH in H_2O, Solvent B: acetonitrile. Typical gradient was from 10% to 90% B within 6 or 10 min with 1 ml/min flow. Preparative RP-HPLC was performed on an UltiMate 3000 system (Thermo Fisher Scientific) on a C18 5 µm, 10 x 250 mm column (Supelco, flow rate 4 mL/min) or a C18 5 µm, 21.2 x 250 mm column (Supelco, flow rate 8 mL/min), solvent A: 0.1% v/v TFA in H2O, solvent B: acetonitrile. Typical gradient was from 10% to 100% B within 60 min. The compounds purified by Prep-HPLC were lyophilized on a lyophilizer (Christ) equipped with a vacuum pump (Vacuubrand). Flash column chromatography was performed with silica gel (230-400 mesh, Silicycle) on an automated purification system (Biotage Isolera One). ^{1}H and ^{13}C nuclear magnetic resonance (NMR) spectra were recorded on a Bruker DPX 400. Data for ^{1}H NMR spectra are reported as follows: chemical shift (δ ppm), multiplicity (s = singlet, d = doublet, t = triplet, q = quartet, dd = doublet of doublets, m = multiplet), coupling constant (Hz), integration. Data for ^{13}C NMR spectra are reported by chemical shift (δ ppm). High resolution mass spectra (HRMS) were measured on a maXis II™ ETD with electron spray ionization (ESI) (Bruker).

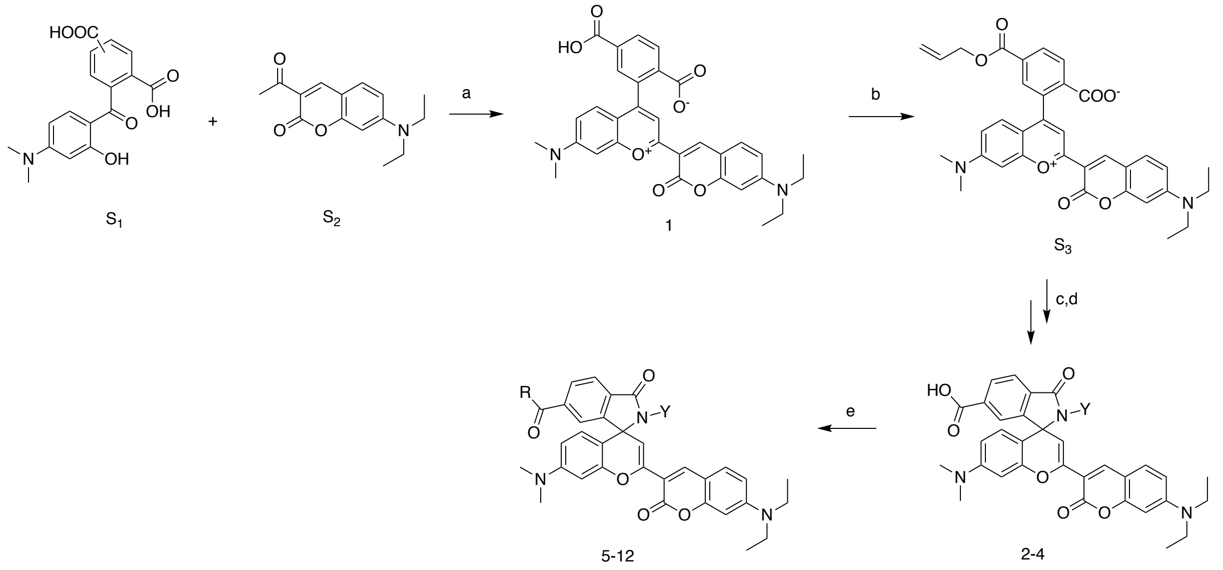

Scheme S1. Synthetic route of color-shifting derivatives: (a) CONC. H_2SO_4, 90 ºC, 3 d; (b) allyl bromide, K_2CO_3, DMF, r.t. 2 h; (c) $POCl_3$, DCM, reflux, 3 h; amines, ACN, DIPEA, 70 ºC, 2 h; (d) 1,3-Dimethylbarbituric acid/$Pd(PPh_3)_4$, MeOH/DCM, r.t. 1 h; (e) PyBOP, DIPEA, DMF, r.t.

Synthesis of fluorophore 1

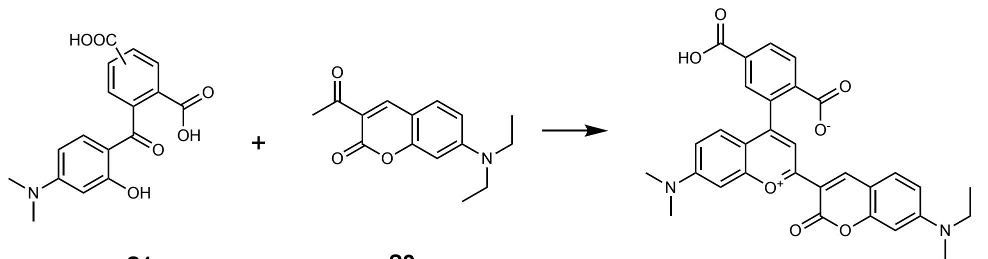

S1 **S2** **1**

Compounds **S1** (isomeric mixture of 4-(4-(dimethylamino)-2-hydroxybenzoyl) isophthalic acid and 2-(4-(dimethylamino)-2-hydroxybenzoyl)terephthalic acid)[1] and **S2**[2] were synthesized according to literature procedures. S1 (0.50 g, 1.52 mmol, 1 equiv.,) and S2 (0.50 g, 1.97 mmol, 1.3 equiv.) were dissolved in sulfuric acid (10 ml) and stirred at 90°C for 3 d (monitored by LC-MS).[3] After cooling to room temperature, the solution was added to ice-cold water and perchloric acid was added (1 ml). The precipitate was filtered and washed with water. Purification by HPLC yielded **1** as a green solid (114 mg, 206 µmol, 14%). ^1H NMR (400 MHz, Methanol-d_4) δ = 9.07 (s, 1H), 8.36 (d, J=1.1, 2H), 8.32 (s, 1H), 8.07 (t, J=1.1, 1H), 7.71 (d, J=9.2, 1H), 7.34 (d, J=9.3, 1H), 7.31 – 7.24 (m, 2H), 6.97 (dd, J=9.2, 2.4, 1H), 6.67 (d, J=2.4, 1H), 3.64 (q, J=7.1, 4H), 3.36 (s, 6H), 1.29 (t, J=7.1, 6H). ^{13}C NMR (101 MHz, Methanol-d_4) δ =

167.9, 167.7, 164.1, 163.5, 160.2, 160.1, 160.0, 158.8, 156.7, 147.4, 138.0, 135.9, 135.4, 134.3, 132.8, 132.5, 131.7, 130.4, 118.4, 117.6, 113.5, 113.1, 111.4, 107.1, 97.8, 97.5, 46.6, 41.0, 12.8. HRMS (ESI$^+$) m/z 553.1969 calcd $[C_{32}H_{29}N_2O_7]^+$ (M+H$^+$), 553.1963 found.

Allyl ester protection of 1

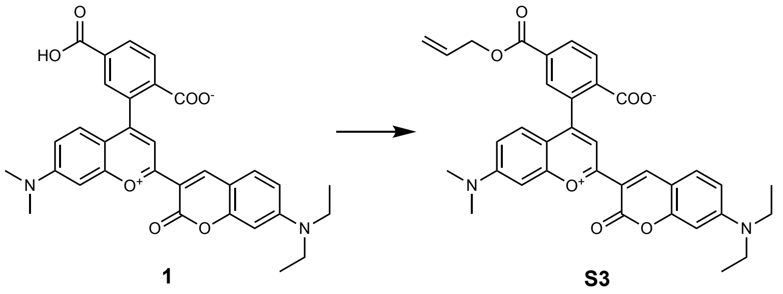

1 (93.0 mg, 168 µmol, 1 equiv.) was dissolved in DMF (2 ml) and caesium carbonate (110 mg, 337 µmol, 2 equiv.) was added. The reaction was cooled down in an ice bath and allyl bromide (17.6 µl, 202 µmol, 1.2 equiv.) was slowly added. Then the mixture was allowed to warm up to r.t. and stirred for 2 h. The reaction was monitored by LC-MS. When starting material was still visible, more allyl bromide was added and the reaction was stirred for another hour. Then the reaction was diluted with DCM (200 ml) and extracted with water (2x 100 ml). The organic phase was washed with brine (1x 20 ml), dried over $MgSO_4$, filtered, and concentrated *in vacuo*. The mixture was purified by flash chromatography on silica gel (0-20% MeOH in DCM) yielding in **S3** as a green solid (62 mg, 105 µmol, 62%). Starting material can be recovered by addition of perchloric acid to the aqueous phase until pH < 1 and filtration of the green precipitate. ^{1}H NMR (400 MHz, DMSO-d_6) δ = 8.65 (s, 1H), 8.23 (dd, *J*=8.1, 1.4, 1H), 8.08 (d, *J*=8.0, 1H), 7.76 (d, *J*=1.3, 1H), 7.67 (d, *J*=9.0, 1H), 6.82 (dd, *J*=9.0, 2.4, 1H), 6.70 (d, *J*=2.3, 1H), 6.63 – 6.49 (m, 3H), 6.40 (s, 1H), 6.01 (ddt, *J*=16.3, 10.8, 5.7, 1H), 5.36 (dd, *J*=17.2, 1.7, 1H), 5.24 (dd, *J*=10.4, 1.5, 1H), 4.79 (dt, *J*=5.6, 1.4, 2H), 3.48 (q, *J*=7.0, 4H), 2.97 (s, 6H), 1.15 (t, *J*=7.0, 6H). ^{13}C NMR (101 MHz, DMSO-d_6) δ = 167.8, 164.3, 158.5, 156.1, 153.4, 151.9, 151.7, 147.3, 141.5, 135.5, 132.2, 130.8, 130.7, 129.6, 128.0, 125.5, 124.2, 118.8, 110.0, 109.9, 109.3, 107.6, 104.6, 98.8, 98.2, 95.9, 84.1, 66.0, 44.2, 12.4. HRMS (ESI$^+$) m/z calcd 593.2282 $[C_{35}H_{33}N_2O_7]^+$ (M+H$^+$), 593.2282 found.

Amide derivatization and allyl deprotection

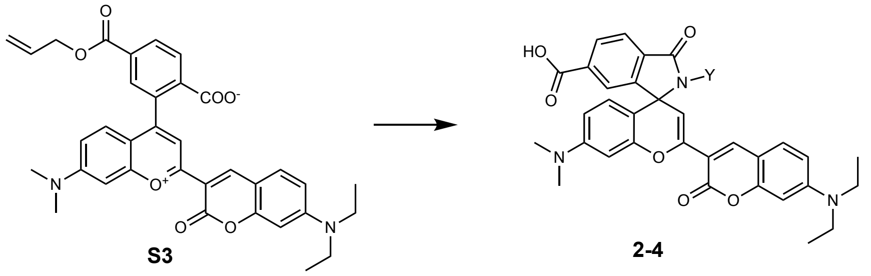

S3 2-4

S3 (1 equiv.) was dissolved in dry DCM (0.5 ml/10 µmol). Phosphorus oxychloride (25 equiv.) was added and the reaction was refluxed for 3 h. DCM and phosphorus oxychloride were removed *in vacuo* and the crude acyl chloride residue was used directly in the next step. Amine (10 equiv.) and DIPEA (15 equiv.) were dissolved in dry acetonitrile (1 ml/10 µmol), transferred into the crude acyl chloride residue while stirring. The reaction was stirred at reflux for 2 h. After removing the acetonitrile the products were mixed with 1,3-dimethyl-1,3-diazinane-2,4,6-trione (5 equiv.) and tetrakis(triphenylphosphine)palladium(0) (0.5 equiv.) in MeOH/DCM (5/1, 2 ml/10 µmol) and stirred at r.t. for 1 h. The reaction was monitored by LC-MS and the product was purified by HPLC.

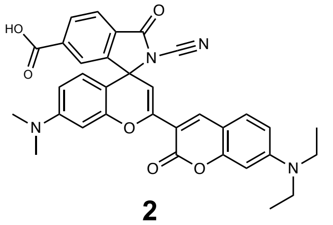

2

20 mg of **S3** coupled with cyanamide yielded in **2** (8.1 mg, 14 µmol 42%) as a dark green solid. ^{1}H NMR (400 MHz, DMSO-d_6) δ = 8.67 (s, 1H), 8.19 (dd, *J*=8.0, 1.4, 1H), 8.08 (d, *J*=8.0, 1H), 7.72 (d, *J*=1.2, 1H), 7.67 (d, *J*=9.0, 1H), 6.83 (dd, *J*=9.0, 2.4, 1H), 6.73 – 6.69 (m, 2H), 6.60 (d, *J*=2.4, 1H), 6.56 (dd, *J*=9.0, 2.6, 1H), 6.42 (s, 1H), 3.48 (q, *J*=7.0, 4H), 2.99 (s, 6H), 1.15 (t, *J*=7.0, 6H). ^{13}C NMR (101 MHz, DMSO-d_6) δ = 166.4, 165.5, 159.0, 156.6, 152.5, 152.3, 152.2, 152.0, 148.4, 142.1, 137.9, 131.2, 131.2, 129.6, 128.3, 125.6, 125.6, 110.9, 110.4, 109.6, 108.0, 107.6, 102.8, 98.9, 97.6, 96.4, 44.7, 12.8. HRMS (ESI$^+$) m/z calcd 577.2082 $[C_{33}H_{29}N_4O_6]^+$ (M+H$^+$), 577.2082 found.

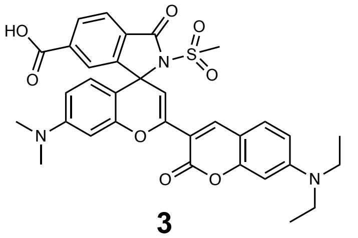

3

20 mg of **S3** coupled with methanesulfonamide yielded in **3** (19 mg, 30 µmol 89%) as a dark green solid. ^{1}H NMR (400 MHz, DMSO-d_6) δ = 13.57 (s, 1H), 8.59 (s, 1H), 8.15 (dd, *J*=8.0, 1.4, 1H), 8.04 (d, *J*=8.0, 1H), 7.68 (d, *J*=9.0, 1H), 7.64 (s, 1H), 6.82 (dd, *J*=9.0, 2.4, 1H), 6.64 – 6.58 (m, 3H), 6.47 (dd, *J*=8.8, 2.5, 1H), 6.32 (s, 1H), 3.48 (q, *J*=7.1, 4H), 3.21 (s, 3H), 2.97 (s, 6H), 1.15 (t, *J*=7.0, 6H). ^{13}C NMR (101 MHz, DMSO-d_6) δ = 166.5, 165.8, 159.1, 156.4, 153.0, 152.4, 152.0, 151.7, 146.8, 141.3, 137.3, 131.0, 130.8, 130.7, 127.9, 125.5, 125.0, 110.4, 110.3, 109.8, 108.1, 106.0, 99.6, 99.0, 96.3, 67.9, 44.7, 42.8, 40.9, 12.8. HRMS (ESI$^+$) m/z calcd 630.1905 $[C_{33}H_{32}N_3O_8S]^+$ (M+H$^+$), 630.1903 found.

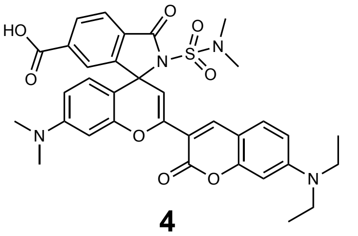

4

20 mg of **S3** coupled with N,N-dimethylsulfamide yielded in **4** (15.6 mg, 24 µmol 70%) as a dark green solid. ^{1}H NMR (400 MHz, DMSO-d_6) δ = 8.60 (s, 1H), 8.11 (dd, *J*=8.0, 1.4, 1H), 7.93 (d, *J*=7.9, 1H), 7.67 (d, *J*=9.0, 1H), 7.60 (s, 1H), 6.81 (dd, *J*=9.0, 2.4, 1H), 6.61 (d, *J*=2.5, 1H), 6.57 (d, *J*=2.4, 1H), 6.55 (d, *J*=8.9, 1H), 6.45 (dd, *J*=8.9, 2.6, 1H), 6.31 (s, 1H), 3.47 (q, *J*=7.1, 4H), 2.95 (s, 6H), 2.81 (s, 6H), 1.14 (t, *J*=7.0, 6H). ^{13}C NMR (101 MHz, DMSO-d_6) δ = 166.3, 165.4, 158.6, 155.9, 152.8, 152.1, 151.4, 151.1, 146.2, 140.5, 130.5, 130.1, 127.6, 125.0, 123.8, 118.9, 115.9, 109.8, 109.8, 109.1, 107.6, 106.0, 99.9, 98.5, 95.8, 67.1, 44.2, 39.9, 37.7, 12.4. HRMS (ESI$^+$) m/z calcd 659.2170 $[C_{34}H_{35}N_4O_8S]^+$ (M+H$^+$), 659.2170 found.

Amide coupling of 6'-carboxylic acid

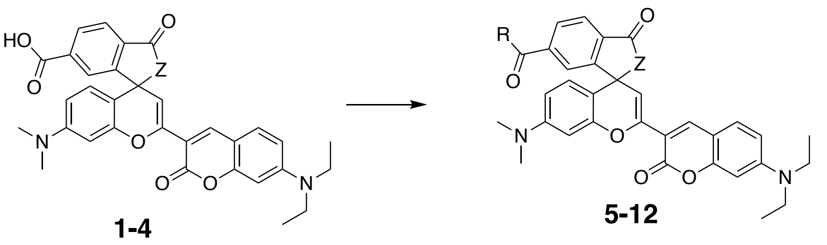

Fluorophore **1-4** (1 equiv.), DIPEA (2 equiv.) and amine (1.5 equiv.) were mixed in DMSO. A solution of PyBOP in DMSO (0.1 mg/µl, 1.1 equiv.) was added, the mixture was stirred for 10 min at r.t. and purified by HPLC.

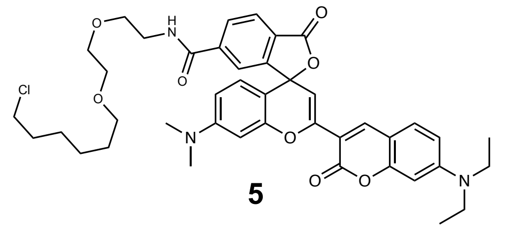

5

Tert-butyl (2-(2-((6-chlorohexyl)oxy)ethoxy)ethyl)carbamate (3.0 mg, 9.3 µmol, 1.5 equiv.) was deprotected with TFA/DCM (1:1). Acid and solvent were removed and the resulting amine was coupled with **1** (3.4 mg, 6.1 µmol, 1 equiv.) resulting in **5** (3.4 mg, 4.5 µmol, 73%) as a green solid. ^{1}H NMR (400 MHz, Methanol-d_4) δ = 8.99 (d, *J*=2.6, 1H), 8.80 (t, *J*=5.4, 1H), 8.36 (d, *J*=8.2, 1H), 8.31 (d, *J*=0.8, 1H), 8.17 (dd, *J*=8.2, 1.8, 1H), 7.90 (d, *J*=1.7, 1H), 7.65 (dd, *J*=9.2, 1.7, 1H), 7.32 – 7.25 (m, 1H), 7.25 (t, *J*=2.1, 1H), 7.23 – 7.15 (m, 1H), 6.95 (dd, *J*=9.1, 2.6, 1H), 6.66 (d, *J*=2.4, 1H), 3.71 – 3.55 (m, 12H), 3.50 (t, *J*=6.6, 2H), 3.44 (t, *J*=6.5, 2H), 3.32 (s, 6H), 1.75 – 1.63 (m, 2H), 1.51 (p, *J*=6.8, 2H), 1.43 – 1.29 (m, 4H), 1.29 (t, *J*=7.0, 6H). ^{13}C NMR (101 MHz, Methanol-d_4) δ = 168.1, 168.1, 164.0, 163.6, 160.0, 160.0, 159.8, 158.7, 156.7, 147.3, 139.5, 138.1, 134.3, 134.3, 132.7, 130.5, 130.2, 129.7, 118.2, 117.5, 113.5, 113.1, 111.5, 106.9, 97.8, 97.5, 72.2, 71.3, 71.2, 70.4, 46.7, 45.7, 41.2, 41.0, 33.7, 30.5, 27.7, 26.4, 12.8. HRMS (ESI$^+$) m/z calcd 758.3203 $[C_{42}H_{49}ClN_3O_8]^+$ (M+H$^+$), 758.3199 found.

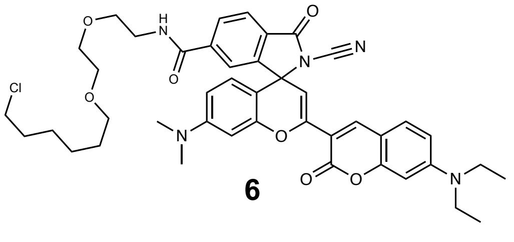

6

Tert-butyl (2-(2-((6-chlorohexyl)oxy)ethoxy)ethyl)carbamate was deprotected with TFA/DCM (1:1). Acid and solvent were removed and the resulting amine was coupled

with **2** (2.0 mg, 3.5 mg, 1 equiv.) resulting in **6** (1.1 mg, 1.4 μmol, 41%) as a green solid. ^{1}H NMR (400 MHz, DMSO-d_6) δ = 8.82 (t, *J*=5.6, 1H), 8.69 (s, 1H), 8.14 (dd, *J*=8.1, 1.4, 1H), 8.06 (d, *J*=8.1, 1H), 7.77 (s, 1H), 7.68 (d, *J*=9.0, 1H), 6.84 (dd, *J*=9.0, 2.5, 1H), 6.72 (d, *J*=2.6, 1H), 6.68 (d, *J*=8.8, 1H), 6.61 (d, *J*=2.4, 1H), 6.56 (dd, *J*=9.0, 2.6, 1H), 6.42 (s, 1H), 3.57 (t, *J*=6.6, 2H), 3.53 – 3.45 (m, 8H), 3.44 – 3.33 (m, 4H), 3.29 (t, *J*=6.5, 2H), 2.99 (s, 6H), 1.64 (p, *J*=6.7, 2H), 1.39 (p, *J*=6.7, 2H), 1.35 – 1.26 (m, 2H), 1.26 – 1.18 (m, 2H), 1.16 (t, *J*=7.0, 6H). ^{13}C NMR (101 MHz, DMSO-d_6) δ = 165.2, 164.5, 158.5, 156.2, 152.0, 151.8, 151.8, 151.6, 147.8, 141.5, 140.9, 130.8, 129.1, 127.9, 127.7, 124.7, 123.2, 110.4, 109.9, 109.1, 107.5, 107.1, 102.6, 98.4, 97.3, 95.9, 70.1, 69.5, 69.3, 68.6, 45.3, 44.2, 32.0, 29.0, 26.1, 24.9, 12.4. HRMS (ESI$^+$) m/z calcd 782.3315 $[C_{43}H_{49}ClN_5O_7]^+$ (M+H$^+$), 782.3309 found.

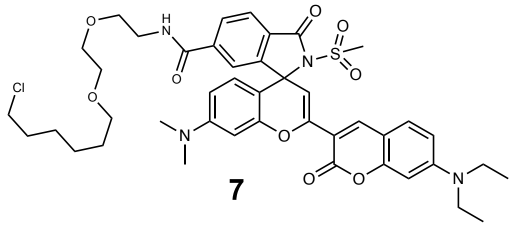

7

Tert-butyl (2-(2-((6-chlorohexyl)oxy)ethoxy)ethyl)carbamate (3.8 mg, 12 μmol, 1.5 equiv.) was deprotected with TFA/DCM (1:1). Acid and solvent were removed and the resulting amine was coupled with **3** (5.0 mg, 7.9 μmol, 1 equiv.) resulting in **7** (3.5 mg, 4.2 μmol, 53%). ^{1}H NMR (400 MHz, DMSO-d_6) δ = 8.78 (t, *J*=5.6, 1H), 8.60 (s, 1H), 8.09 (dd, *J*=8.1, 1.4, 1H), 8.00 (d, *J*=8.0, 1H), 7.67 (d, *J*=8.9, 1H), 7.67 (s, 1H), 6.82 (dd, *J*=9.0, 2.4, 1H), 6.64 – 6.56 (m, 3H), 6.46 (dd, *J*=8.9, 2.6, 1H), 6.31 (s, 1H), 3.57 (t, *J*=6.6, 2H), 3.40 (dd, *J*=5.8, 3.4, 2H), 3.35 (q, *J*=5.7, 2H), 3.28 (t, *J*=6.5, 2H), 3.19 (s, 3H), 2.96 (s, 6H), 1.63 (p, *J*=6.8, 2H), 1.43 – 1.18 (m, 6H), 1.15 (t, *J*=7.0, 6H). ^{13}C NMR (101 MHz, DMSO-d_6) δ = 165.5, 164.7, 162.6, 158.6, 155.9, 152.5, 152.0, 151.5, 151.2, 146.2, 140.6, 140.3, 130.5, 128.9, 128.6, 127.5, 124.1, 123.1, 109.9, 109.8, 109.3, 107.6, 105.7, 99.3, 98.6, 95.9, 70.1, 69.5, 69.3, 68.6, 67.5, 65.2, 45.3, 44.2, 42.3, 32.0, 29.0, 26.1, 24.9, 12.4. HRMS (ESI$^+$) m/z calcd 835.3138 $[C_{43}H_{52}ClN_4O_9S]^+$ (M+H$^+$), 835.3140 found.

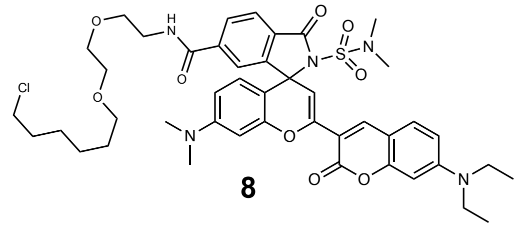

8

Tert-butyl (2-(2-((6-chlorohexyl)oxy)ethoxy)ethyl)carbamate (1.5 mg, 4.6 µmol, 1.5 equiv.) was deprotected with TFA/DCM (1:1). Acid and solvent were removed and the resulting amine was coupled with **4** (2.0 mg, 3.0 µmol, 1 equiv.) resulting in **8** (1.4 mg, 1.6 µmol, 53%) as a gren solid. ^{1}H NMR (400 MHz, DMSO-d_6) δ = 8.76 (t, *J*=5.6, 1H), 8.61 (s, 1H), 8.08 (dd, *J*=8.1, 1.5, 1H), 7.95 (d, *J*=8.0, 1H), 7.68 (d, *J*=9.1, 1H), 7.66 (d, *J*=1.5, 1H), 6.82 (dd, *J*=9.0, 2.4, 1H), 6.62 (d, *J*=2.5, 1H), 6.57 (d, *J*=2.4, 1H), 6.53 (d, *J*=8.8, 1H), 6.45 (dd, *J*=8.9, 2.5, 1H), 6.32 (s, 1H), 3.57 (t, *J*=6.6, 2H), 3.52 – 3.42 (m, 8H), 3.44 – 3.36 (m, 2H), 3.35 (q, *J*=5.6, 2H), 3.28 (t, *J*=6.5, 2H), 2.95 (s, 6H), 2.81 (s, 6H), 1.63 (d, *J*=6.7, 2H), 1.38 (p, *J*=6.7, 2H), 1.36 – 1.16 (m, 4H), 1.15 (t, *J*=7.0, 6H). ^{13}C NMR (101 MHz, DMSO-d_6) δ = 165.3, 164.7, 158.6, 155.9, 152.8, 152.2, 151.4, 151.1, 146.2, 140.5, 140.0, 130.5, 128.9, 128.5, 127.7, 123.8, 123.2, 109.8, 109.1, 107.6, 106.0, 99.9, 98.6, 95.8, 70.1, 69.5, 69.3, 68.6, 67.3, 45.3, 44.2, 32.0, 29.0, 26.1, 24.8, 12.4. HRMS (ESI$^+$) m/z calcd 864.3404 $[C_{44}H_{55}ClN_5O_9S]^+$ (M+H$^+$), 864.3409 found.

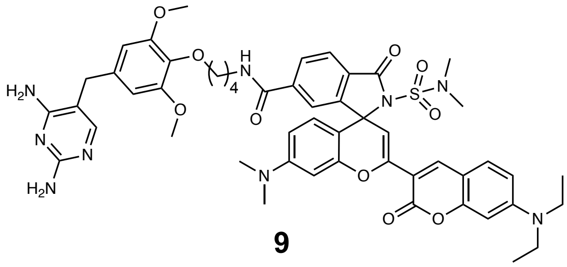

9

5-(4-(4-aminobutoxy)-3,5-dimethoxybenzyl)pyrimidine-2,4-diamine[4] was coupled with **4** (1.0 mg, 1.8 µmol) yielding in **9** (1.1 mg, 1.1 µmol, 62%) as a green solid ^{1}H NMR (400 MHz, DMSO-d_6) δ = 8.71 (t, *J*=5.7, 1H), 8.61 (s, 1H), 8.28 (s, 1H), 8.08 (dd, *J*=8.1, 1.5, 1H), 7.96 (d, *J*=8.0, 1H), 7.75 (s, 1H), 7.68 (d, *J*=8.9, 1H), 7.64 (s, 1H), 7.52 (s, 2H), 7.38 (d, *J*=4.1, 1H), 6.82 (dd, *J*=9.0, 2.4, 1H), 6.61 (d, *J*=2.5, 1H), 6.59 – 6.51 (m, 4H), 6.45 (dd, *J*=8.9, 2.5, 1H), 6.32 (s, 1H), 3.77 (t, *J*=5.6, 2H), 3.68 (s, 6H), 3.56 (s, 2H), 3.47 (q, *J*=7.0, 4H), 3.25 (q, *J*=6.3, 2H), 2.95 (s, 6H), 2.80 (s, 6H), 1.66 – 1.54 (m, 4H), 1.14 (t, *J*=7.0, 6H). HRMS (ESI$^+$) m/z calcd 494.7047 $[C_{51}H_{59}N_9O_{10}S]^{2+}$ (M+2H$^+$), 494.7044 found.

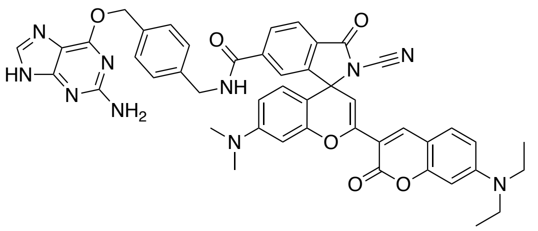

10

Compound **2** (5.0 mg, 8.7 µmol, 1eq.) was coupled with 6-((4-(aminomethyl)benzyl)oxy)-7H-purin-2-amine according to general procedure **II** yielding as **10** (1.4 mg, 1.7 µmo, 19%) as a blue solid.

^{1}H NMR (400 MHz, DMSO-d_6) δ = 9.31 (t, *J*=6.0, 1H), 8.68 (s, 1H), 8.19 (dd, *J*=8.0, 1.4, 1H), 8.07 (d, *J*=8.0, 1H), 7.85 – 7.78 (m, 2H), 7.68 (d, *J*=9.1, 1H), 7.43 (d, *J*=7.9, 2H), 7.29 (d, *J*=7.9, 2H), 6.83 (dd, *J*=9.1, 2.4, 1H), 6.72 – 6.66 (m, 2H), 6.61 (d, *J*=2.3, 1H), 6.56 (dd, *J*=9.0, 2.6, 1H), 6.41 (s, 1H), 6.28 (s, 2H), 5.43 (s, 2H), 4.44 (d, *J*=5.9, 2H), 3.49 (q, *J*=7.2, 4H), 2.99 (s, 6H), 1.16 (t, *J*=7.0, 6H).

HRMS (ESI$^+$) m/z calcd 415.1639 $[C_{46}H_{42}N_{10}O_6]^{2+}$ (M+2H$^+$), 415.1638 found.

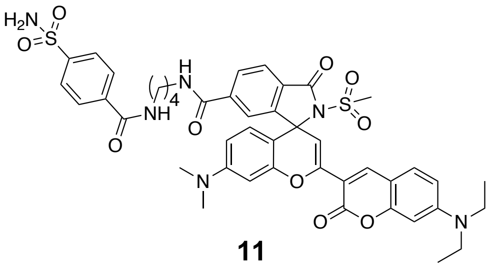

11

Tert-butyl (4-(4-sulfamoylbenzamido)butyl)carbamate[5] (1.2 mg, 3.2 µmol, 2 equiv.) was deprotected with TFA/DCM (1:1). Acid and solvent were removed and the resulting amine was coupled with **3** to produce **11**. Yield: 7.4 mg, 8,3 µmol, 53%. ^{1}H NMR (400 MHz, DMSO-d_6) δ = 8.74 (t, *J*=5.7, 1H), 8.64 – 8.57 (m, 2H), 8.10 (dd, *J*=8.1, 1.5, 1H), 8.01 (d, *J*=8.0, 1H), 7.95 (d, *J*=8.5, 2H), 7.87 (d, *J*=8.4, 2H), 7.72 – 7.64 (m, 2H), 7.46 (s, 2H), 6.83 (dd, *J*=9.0, 2.5, 1H), 6.65 – 6.57 (m, 3H), 6.47 (dd, *J*=8.9, 2.6, 1H), 6.32 (s, 1H), 3.48 (q, *J*=7.1, 4H), 3.28 – 3.22 (m, 4H), 3.19 (s, 3H), 2.96 (s, 6H), 1.53 (p, *J*=3.3, 4H), 1.15 (t, *J*=7.0, 6H). HRMS (ESI$^+$) m/z calcd 883.2790 $[C_{44}H_{47}N_6O_{10}S_2]^+$ (M+H$^+$), 883.2790 found.

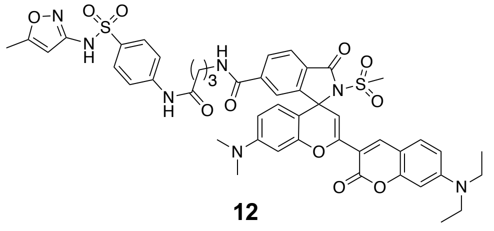

12

(9H-fluoren-9-yl)methyl (4-(4-(N-(5-methylisoxazol-3-yl)sulfamoyl)benzamido)butyl) carbamate[6] (4.45 mg, 7.94 µmol, 5 equiv.) was deprotected in DMF with 2% DBU and used as amine yielding in **12** (0.44 mg, 4.6 µmol, 29%) as green solid. ^{1}H NMR (400 MHz, DMSO-d_6) δ = 11.28 (s, 1H), 10.27 (s, 1H), 8.75 (t, *J*=5.7, 1H), 8.60 (s, 1H), 8.09 (dd, *J*=8.1, 1.5, 1H), 8.00 (d, *J*=8.0, 1H), 7.81 – 7.59 (m, 6H), 6.82 (dd, *J*=9.0, 2.4, 1H), 6.64 – 6.56 (m, 3H), 6.46 (dd, *J*=8.9, 2.6, 1H), 6.31 (s, 1H), 6.10 (d, *J*=1.1, 1H), 3.47 (q, *J*=7.1, 4H), 3.19 (s, 3H), 2.96 (s, 6H), 2.27 (s, 3H), 1.84 – 1.72 (m, 2H), 1.15 (t, *J*=7.0, 6H). HRMS (ESI$^+$) m/z calcd 950.2848 $[C_{47}H_{48}N_7O_{11}S_2]^+$ (M+H$^+$), 950.2847 found.

Spectroscopic characterization of free color-shifting fluorophores. Fluorescent molecules were prepared as stock solutions in DMSO and diluted such that the DMSO concentration did not exceed 1% (v/v). Absorption and fluorescence emission spectra were measured at room temperature (RT, 25 °C) in flat transparent bottom 384-well plates (Thermo Fisher) on Spark20® microplate reader (Tecan) in buffer HEPES 50 mM, pH 7.3, NaCl 50 mM and 0.1% (w/v) Triton X-100 (Sigma-Aldrich) unless otherwise noted (dubbed as HEPES buffer in the following). Measurements were performed in presence of 0.1% (w/v) SDS (Carl Roth) to support measurements of the red fluorescent, zwitterionic form of the CSFs. Maximum absorption wavelengths (λ_{abs}^{max}) and maximum fluorescence emission wavelengths (λ_{emi}^{max}) were determined from the spectra profiles.

The absorption measurements were performed with concentration ranging from 0.25 µM to 1 µM. Molar extinction coefficients were calculated from the absorbance values according to equation 1. Reported values for molar extinction coefficient were averaged (n = 3). Normalized spectra to the absorbance at λ_{abs}^{max} are shown for clarity.

Equation 1: $Abs = \varepsilon \cdot \mathrm{l} \cdot \mathrm{c}$

Where ε is the molar extinction coefficient (M^{-1}.cm^{-1}), l is the path length of measurement (cm) and c the concentration (M). In the current set up, a path length of 1 cm was employed for a final volume of 103 µL in 384 well plate.

The green fluorescence was measured by exciting the fluorophores at 425 nm (slit width = 10 nm) and measuring their fluorescence emission profile from 450 nm to 610 nm (slit width = 10 nm). The red fluorescence was measured by exciting the fluorophores at 640 nm (slit width = 10 nm) and measuring their fluorescence emission profile from 665 nm to 850 nm (slit width = 10 nm). Among triplicate spectra, one representative is depicted in figures.

Absolute fluorescence quantum yields were recorded on a Quantaurus QY C11347 spectrometer (Hamamatsu photonics, Shizuoka) at RT by exciting fluorophores at 410 nm and 640 nm for green and red fluorescence, respectively. Measurements were carried out using samples concentration leading to an absorbance < 0.1 and self-absorption corrections were performed using the instrument software.[7] Reported values are averages of triplicate experiments.

Titration of color-shifting fluorophores in water-dioxane mixtures. The absorbance spectra of compounds **1-4** (10 µM) were recorded at RT in water-dioxane mixtures (10%, 20%, 30%, 40%, 50%, 60%, 70%, 80% and 90% (v/v)) using 96-well plates (Greiner bio-one) on a Spark20® microplate reader (Tecan). The absorbance ratio in the red/green channels (*i.e.* A_{665}/A_{425}) were employed to determine the equilibrium spirocyclic/zwitterionic ratio. The absorbance was normalized to the maximum ratio of compound **1**. The normalized absorbance ratio was plotted against dielectric constant deduced from the water-dioxane mixture.[8] From the spectra D_{50} values were extracted. The D_{50} values represent the dielectric constant values at which the equilibrium between the spirocyclic and the zwitterionic form of the different CSFs is 1.

Molecular cloning. All plasmids were cloned and amplified using *Escherichia coli* stains *E. cloni* 10G (Lucigen) or XL1-blue (Agilent). Cloning were performed using the Gibson assembly method[9] in pET51b(+) plasmid (Novagen) for protein expression in *E. coli* and in pCDNA5/FRT (ThermoFisher) for protein expression in mammalian cells. Cloning strategies were designed using the Geneious software, primers were obtained from Eurofins and Microsynth. DNA sequences were verified by Sanger sequencing (Eurofins). Purified DNA was conserved at -20 °C and quantified using a NanoDrop 2000 (ThermoFisher).

Protein expression and purification. The proteins were expressed in the *E. coli* strains BL21(DE3) or Rosetta-gami(DE3). Bacterial cultures were performed in LB medium at 37 °C to an $OD_{600\ nm}$ of 0.6 - 0.8, at which point the temperature was lowered to 16 °C, and 0.5 mM isopropyl β-D-thiogalactopyranoside (IPTG) was added. After 16 h, the cells were harvested by centrifugation (15 min, 4 ºC, 4500 g) and lysed by sonication (SONOPULS HD 2070, Bandelin, 70% power, cycle 50, 7 min on wet ice). The cell extracts were cleared by centrifugation (30 min, 4 ºC, 75 000 g) and purified by two sequential affinity purification on Ni-NTA column (Qiagen) and Strep-Tactin column (IBA) according to the suppliers' instructions. Protein's purity was verified by SDS-PAGE and coomassie blue coloration. Proteins were concentrated using concentration units (Amicon Ultra-0.5 mL, Merck) and stored in 45% (v/v) glycerol at - 20 ºC in 75 mM NaCl, 50 mM TRIS·HCl, pH 7.4.

Spectroscopic characterization of HaloTag7-based CSF probes. SNAP-HaloTag7 (5 µM) was labelled with CSF probes **5-8** (1 µM) in HEPES buffer supplemented with 0.1% Triton X-100 for 1 h at RT. Absorbance and fluorescence emission spectra were recorded in black, transparent and flat bottom 96-well plates (Thermo Fisher Scientific) as previously explained. Control experiments in presence of SDS (0.1%, w/v) were performed to promote zwitterionic form of the CSFs. Reported values are averages of triplicate experiments. Reported spectra correspond to a chosen representative among triplicate measurements.

rHCaMP screening and characterization. The potential HaloTag7-based chemogenetic Ca^{2+} biosensors were designed based on structural analysis of the HaloTag7 crystal structure 4kaj using PyMol.[10] The different variants (Table S3) were cloned, produced and purified as previously explained. Proteins (2.5 µM) were labeled with CSF **7** (0.5 µM) in HEPES buffer supplemented with Bovine Serum Albumin (0.5 mg/mL) overnight at RT. In a black flat bottom 384-well plate, the proteins (1.25 µM) were incubated in presence of either EGTA (1 mM) or $CaCl_2$ (1 mM) for 30 min prior to fluorescence emission spectra recording as previously explained. The ratios of fluorescence intensities at ${}^{green}\lambda_{em}^{max}/{}^{red}\lambda_{em}^{max}$ (as described in Table S2) were compared in presence and absence of Ca^{2+} from which the Ratio Change (RC) of the sensor candidates were calculated according to equation 2.

$$\text{Equation 2: } \mathrm{RC} = \frac{(\mathrm{F475/F692})^{[\mathrm{CaCl2}]}}{(\mathrm{F475/F692})^{[\mathrm{EGTA}]}}$$

Where F_{475} and F_{692} represent fluorescence intensities in the green (475 nm) and red (692 nm) channels. The ratio of these two fluorescence intensities are compared in presence and absence (*i.e.* EGTA) of Ca^{2+}.

An additional batch of the best candidate, rHCaMP, was produced and double purified as previously explained. Its correct mass was confirmed by LC-ESI-MS analysis. rHCaMP (5 µM) was labeled with probe **8** (1 µM) at RT for overnight in HEPES buffer. The excess of dye was removed using an illustra MicroSpin G-50 column desalting device (GE-Healthcare). The labeled protein (0.25 µM) was incubated 30 min at RT in HEPES buffer supplemented with EGTA (1 mM) or $CaCl_2$ (1 mM). The fluorescence emission profiles were recorded as previously explained.

Finally, rHCaMP-**8** (0.25 µM) was titrated in presence of different free Ca^{2+} concentration [5.7 nM-39 µM] using a Calcium Calibration Buffer Kit (Life Technology) as recommended by the manufacturer. After incubation for 30 min at 37 ºC, the fluorescence emission spectra were recorded as previously described. The data were plotted to the equation 2 allowing to extract the c_{50} value.

$$\text{Equation 3: } R = R_{min} + \frac{R_{max} - R_{min}}{1 + \left(\frac{c_{50}}{[analyte]}\right)^n}$$

Where R is a ratio measured at a given concentration of analyte ([analyte]), R_{min} and R_{max} are the two extreme ratio values obtained, n is Hill coefficient describing the cooperative character of the response and c_{50} is the analyte concentration at which the R value is half R_{max} - R_{min}. The sensor RC can be calculated from titration curves by calculation R_{max}/R_{min}.

Labeling kinetics measurement

The fluorescence intensity change (λ_{ex}: 640 nm, λ_{em}: 695 nm) over time of the probes **5-8** (50 nM) was followed in presence of HaloTag7 (250 nM) in HEPES buffer without triton at 37 ºC for 60 min (200 µL) using a microplate reader (Spark®, Tecan). Experiments were performed in triplicate and background measurements were carried out in absence of HaloTag7 protein. After background subtraction, the data were normalized to maximum intensities and plotted to a one-phase association equation (4) using the GraphPad Prism software to obtain the pseudo-first order kinetic constants *k* from which the second order kinetic rate constants k_2 were calculated from the equation (5). Value are reported as mean ± s.d. from triplicate experiments.

$$\text{Equation 4: } FI = FI_0 + (FI_{max} - FI_0) * (1 - exp(-k * t))$$

With FI_0 = the initial fluorescence intensity (FI) value, FI_{max} = the maximum FI value reached, t = the time in seconds and *k* = the first order kinetic constant of reaction.

Equation 5: $k_2 = k / [\text{Protein}]$

With [Protein] corresponds to the initial protein concentration and k_2 = the second order kinetic rate constant.

Characterization of CSF-based probes for related binding proteins.

Color-shifting fluorescent probes were mixed with target proteins (eDHFR-SNAP-**9**, Halo-SNAP-**10**, HCAII-**11**, $eDHFR^{mt}$ (R44L and H45Q)-SNAP-**9** or hSPR-**12**) in HEPES buffer with the following concentrations:

- eDHFR-SNAP (2 µM) with **9** (1 µM),
- Halo-SNAP (5 µM) with **10** (1 µM)
- HCAII (10 µM) with **11** (2.5 µM),
- eDHFR mutant-SNAP (2 µM) with **9** (1 µM) ± NADPH (10 µM) and
- hSPR (2 µM) with **12** (0.5 µM) + NADP+ (100 µM).

Absorbance and fluorescence spectra were recorded in black, transparent and flat bottom 96-well plates (Thermo Fisher Scientific) after 60 minutes incubation at RT as previously explained. Control experiments in presence of SDS (0.1 %, w/v) were performed to promote zwitterionic form of the CSFs. Reported values are averages of triplicate experiments. Reported spectra are chosen representatives among triplicate measurements.

The different sensors were titrated in HEPES buffer at RT and as following:

- eDHFR-SNAP-**9** with methotrexate (MTX, Sigma-Aldrich, 10 nM – 100 µM) and
- $eDHFR^{mt}$-SNAP-**9** with reduced nicotinamide adenine dinucleotide phosphate (NADPH, PanReac AppliChem, 100 pM – 500 µM)

Probes **9** was used at 125 nM while proteins were employed at 250 nM. After 30 min incubation at RT, the fluorescence emission spectra were recorded as previously described. The data were plotted to the equation 2 allowing to extract the c_{50} value and the maximum Ratio Change (RC) of the sensors as described above.

General cell culture and fluorescence microscopy

U2OS cells were cultured in Dulbecco’s modified Eagle medium (DMEM, 4.5 g/L Glucose) supplemented with 10% (v/v) FBS (fetal bovine serum), 1 mM GlutaMAX, and 1 mM Sodium pyruvate (Thermo Fisher Scientific). The cells were maintained at

37 °C in a humidified 5% (v/v) CO_2 environment. Cell lines were split every 3-4 days or at confluence and underwent regular mycoplasma testing. Cells were imaged on a Leica TCS SP8 confocal microscope. The cells were incubated for 1-2 days before imaging. Imaging medium contains phenol red free Dulbecco's Modified Eagle Medium (DMEM) media with 10% FBS, 1 mM pyruvate, and 2 mM L-Glutamine (Thermo Fisher Scientific).

Generation of FlpIn rHCaMP-expressing U2OS cell line and live-cell microscopy. The Flp-In™ System (ThermoFisher Scientific) was used to generate U2OS FlpIn rHCaMP-expressing cells as recommended by the manufacturer. Briefly, pcDNA5/FRT-rHCaMP and pOG44 were co-transfected into the host cell line U2OS FLpIn.[11] The homologous recombination between the FRT sites on pcDNA5-FRT-rHCaMP and on the host cell chromosome, promoted by pOG44, allowed to generate the U2OS FlpIn rHCaMP cell line.

U2OS FlpIn rHCaMP cells were labelled with probe **8** (500 nM) overnight at 37 °C in a 5% CO_2 atmosphere in imaging medium containing phenol red free DMEM media that contained 10% FBS, 1 mM pyruvate, and 2 mM L-Glutamine (Thermo Fisher Scientific). Using the same medium, cells were washed 3 times and incubated for another 6 hours. Live cell fluorescence imaging was performed on a Leica SP8 confocal scanning microscope equipped with a white light laser, HC PL APO CS2 40.0x water objective lens and spectral HyD detector. The green fluorescence was recorded by exciting the probe at λ_{ex} = 405 nm and with a detection range of 460 – 550 nm. The green fluorescence was recorded by exciting the probe at λ_{ex} = 660 nm and with a detection range of 670 – 750 nm. The images were collected at 10 different z-focal plan over 20 µm. Cells were treated with ionomycin (Sigma-Aldrich, 1 µM) or with a corresponding DMEM concentration of 50% (v/v). Time course experiments were followed by taking pictures every 1 minute over 15 minutes total.

Structural analysis and modeling. Structural analysis and representation were performed using the Pymol software[10] and crystal structures available on the pdb. The HaloTag7 crystal structure (4kaj), CaM/M13 from the GCaMP2 crystal structure (3evr) and the minimal Troponin domain NMR structure (2m97) were used for the design and representation of rHCaMP. The insertion sites for the calcium sensitive domains were chosen by structural analysis of the HaloTag7 protein structure (4kaj) based on two

main criteria: (i) vicinity to the fluorophore binding site and (ii) foreseen minimal disruption of the protein scaffold upon insertion. The eDHFR crystal structure bound the NAPDH and TMP was extracted from the GFP enhancer nanobody fusion of LAMA-GFP-F98 (6rul) for analysis and representation purposes. For accuracy, the structure was compared with the MTX bound eDHFR (1rg7) and the NADPH/TMP bound *staphylococcus aureus* DHFR (3fre) crystal structures. Analysis and representations of SNAP-tag HCAII and hSPR were performed using benzyl-guanine (4kzz), benzenesulfonamide (6gdc) and $NADP^+$/sulfasalazine (4j7x) bound crystal structures, respectively.

Data analysis. The displayed absorption spectra in Figure S10 and S11 was smoothed in Prism 7 using 10 neighbours to average and 4th order of smoothing polynomial. Fluorescence images were analysed in ImageJ Fiji (http://fiji.sc/wiki/index.php/Fiji)[12]. Images were presented as maximum intensity projections (z-stack, 10 slices, 20 µm) with background correction in each channel. Different region of interests (ROIs) corresponding to cell bodies were manually defined for quantitative data analysis. Ratiometric projections were generated for illustrative purposes.

Reference

1. Kvach, M. V.; Stepanova, I. A.; Prokhorenko, I. A.; Stupak, A. P.; Bolibrukh, D. A.; Korshun, V. A.; Shmanai, V. V., Practical synthesis of isomerically pure 5- and 6-carboxytetramethylrhodamines, useful dyes for DNA probes. *Bioconjug Chem* **2009,** *20* (8), 1673-82.
2. Wu, J. S.; Sheng, R. L.; Liu, W. M.; Wang, P. F.; Zhang, H. Y.; Ma, J. J., Fluorescent sensors based on controllable conformational change for discrimination of Zn2+ over Cd2+. *Tetrahedron* **2012,** *68* (27-28), 5458-5463.
3. Czerney, P.; Graness, G.; Birckner, E.; Vollmer, F.; Rettig, W., Molecular Engineering of Cyanine-Type Fluorescent and Laser-Dyes. *J Photoch Photobio A* **1995,** *89* (1), 31-36.
4. Xue, L.; Prifti, E.; Johnsson, K., A General Strategy for the Semisynthesis of Ratiometric Fluorescent Sensor Proteins with Increased Dynamic Range. *Journal of the American Chemical Society* **2016,** *138* (16), 5258-61.
5. Brun, M. A.; Griss, R.; Reymond, L.; Tan, K. T.; Piguet, J.; Peters, R. J.; Vogel, H.; Johnsson, K., Semisynthesis of fluorescent metabolite sensors on cell surfaces. *J Am Chem Soc* **2011,** *133* (40), 16235-42.
6. Sallin, O.; Reymond, L.; Gondrand, C.; Raith, F.; Koch, B.; Johnsson, K., Semisynthetic biosensors for mapping cellular concentrations of nicotinamide adenine dinucleotides. *Elife* **2018,** *7.*
7. Suzuki, K.; Kobayashi, A.; Kaneko, S.; Takehira, K.; Yoshihara, T.; Ishida, H.; Shiina, Y.; Oishic, S.; Tobita, S., Reevaluation of absolute luminescence quantum yields of standard

solutions using a spectrometer with an integrating sphere and a back-thinned CCD detector. *Phys Chem Chem Phys* **2009,** *11* (42), 9850-9860.
8. Critchfield, F. E.; Gibson, J. A.; Hall, J. L., Dielectric Constant for the Dioxane Water System from 20 to 35-Degrees. *J Am Chem Soc* **1953,** *75* (8), 1991-1992.
9. Gibson, D. G.; Young, L.; Chuang, R. Y.; Venter, J. C.; Hutchison, C. A.; Smith, H. O., Enzymatic assembly of DNA molecules up to several hundred kilobases. *Nat Methods* **2009,** *6* (5), 343-U41.
10. DeLano, W. L., Pymol: An open-source molecular graphics tool. CCP4 Newsletter On Protein. *Crystallography* **2002,** *40*.
11. Malecki, M. J.; Sanchez-Irizarry, C.; Mitchell, J. L.; Histen, G.; Xu, M. L.; Aster, J. C.; Blacklow, S. C., Leukemia-associated mutations within the NOTCH1 heterodimerization domain fall into at least two distinct mechanistic classes. *Molecular and Cellular Biology* **2006,** *26* (12), 4642-4651.
12. Schindelin, J.; Arganda-Carreras, I.; Frise, E.; Kaynig, V.; Longair, M.; Pietzsch, T.; Preibisch, S.; Rueden, C.; Saalfeld, S.; Schmid, B.; Tinevez, J. Y.; White, D. J.; Hartenstein, V.; Eliceiri, K.; Tomancak, P.; Cardona, A., Fiji: an open-source platform for biological-image analysis. *Nat Methods* **2012,** *9* (7), 676-82.
13. J. B. Grimm, B. P. English, J. Chen, J. P. Slaughter, Z. Zhang, A. Revyakin, R. Patel, J. J. Macklin, D. Normanno, R. H. Singer, T. Lionnet, L. D. Lavis, Nat. Methods 2015, 12, 244-250.
14. D. Cao, Z. Liu, P. Verwilst, S. Koo, P. Jangjili, J. S. Kim, W. Lin, Chem. Rev. 2019, 119, 10403-10519

Supplementary NMR spectra

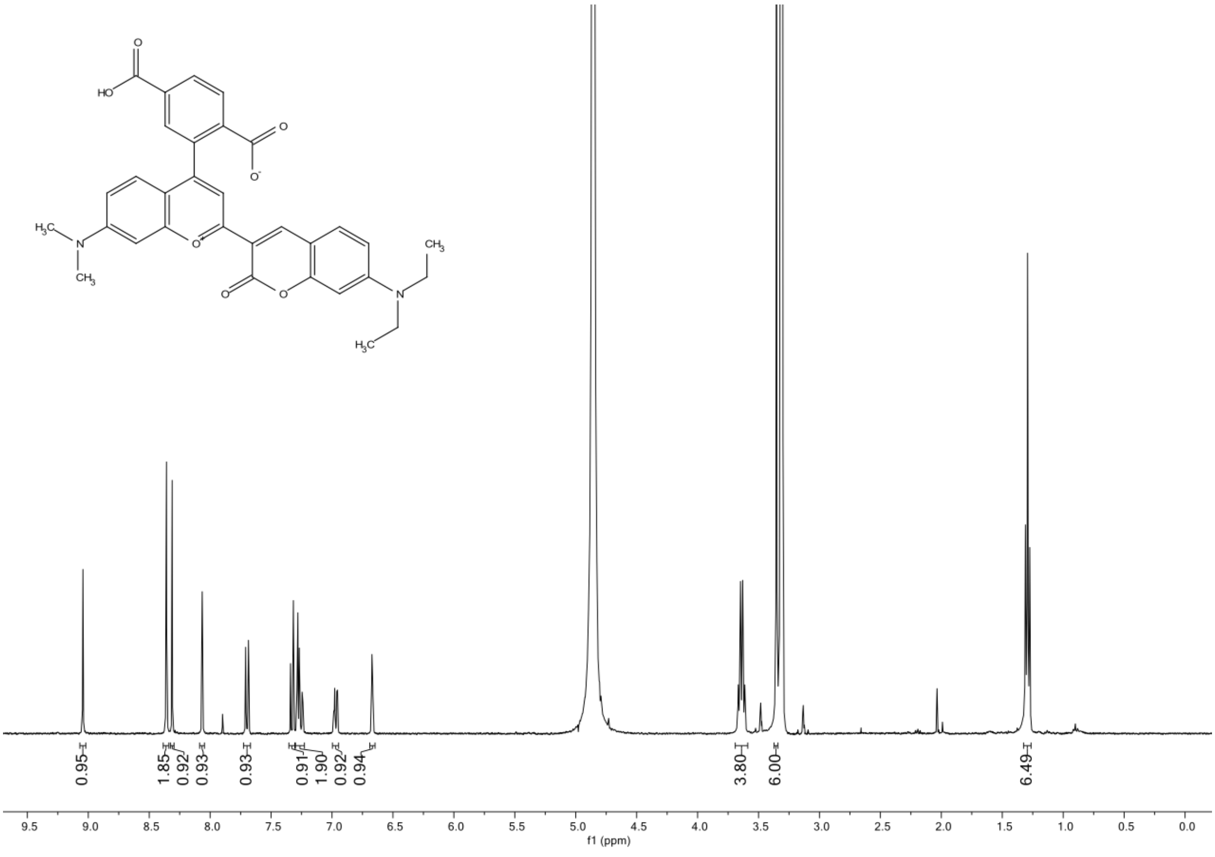

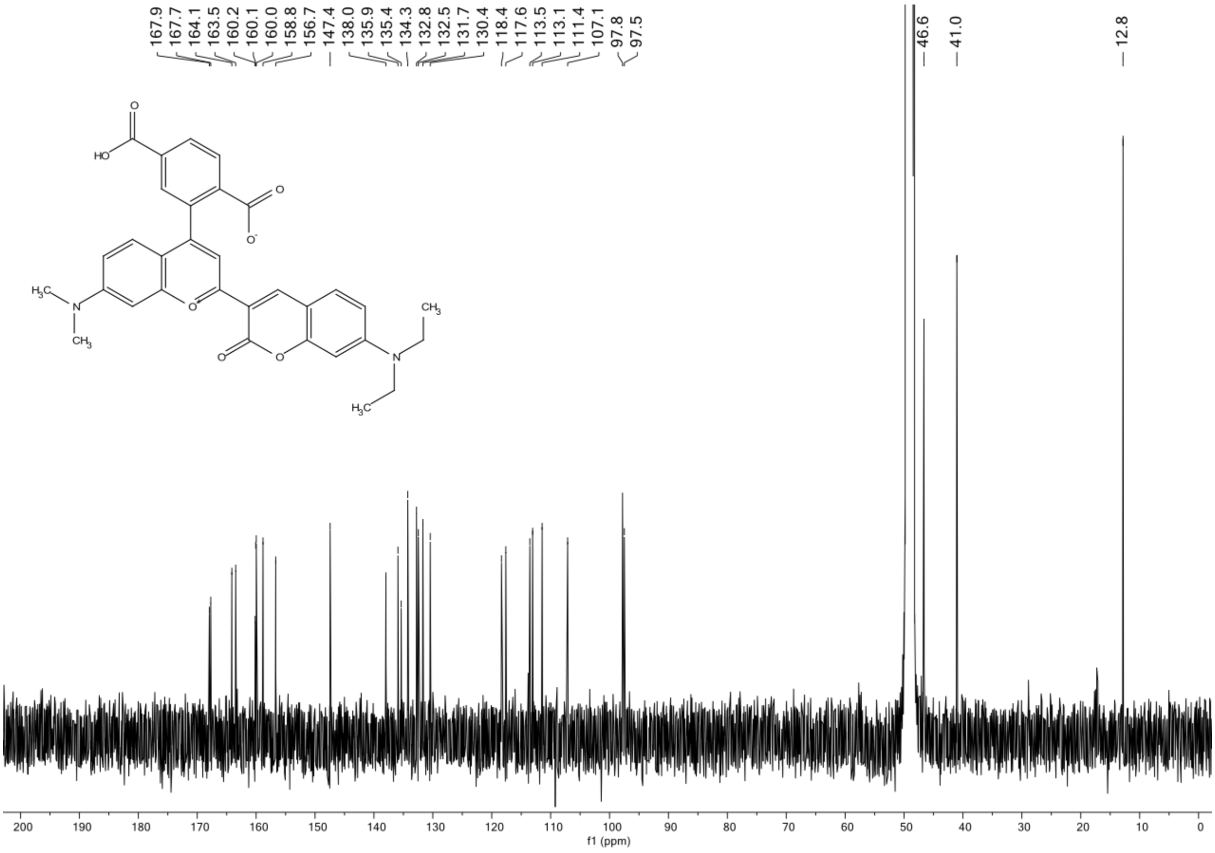
167.9
167.7
164.1
163.5
160.2
160.1
160.0
158.8
156.7
147.4
138.0
135.9
135.4
134.3
132.8
132.5
131.7
130.4
118.4
117.6
113.5
113.1
111.4
107.1
97.8
97.5
46.6
41.0
12.8
f1 (ppm)

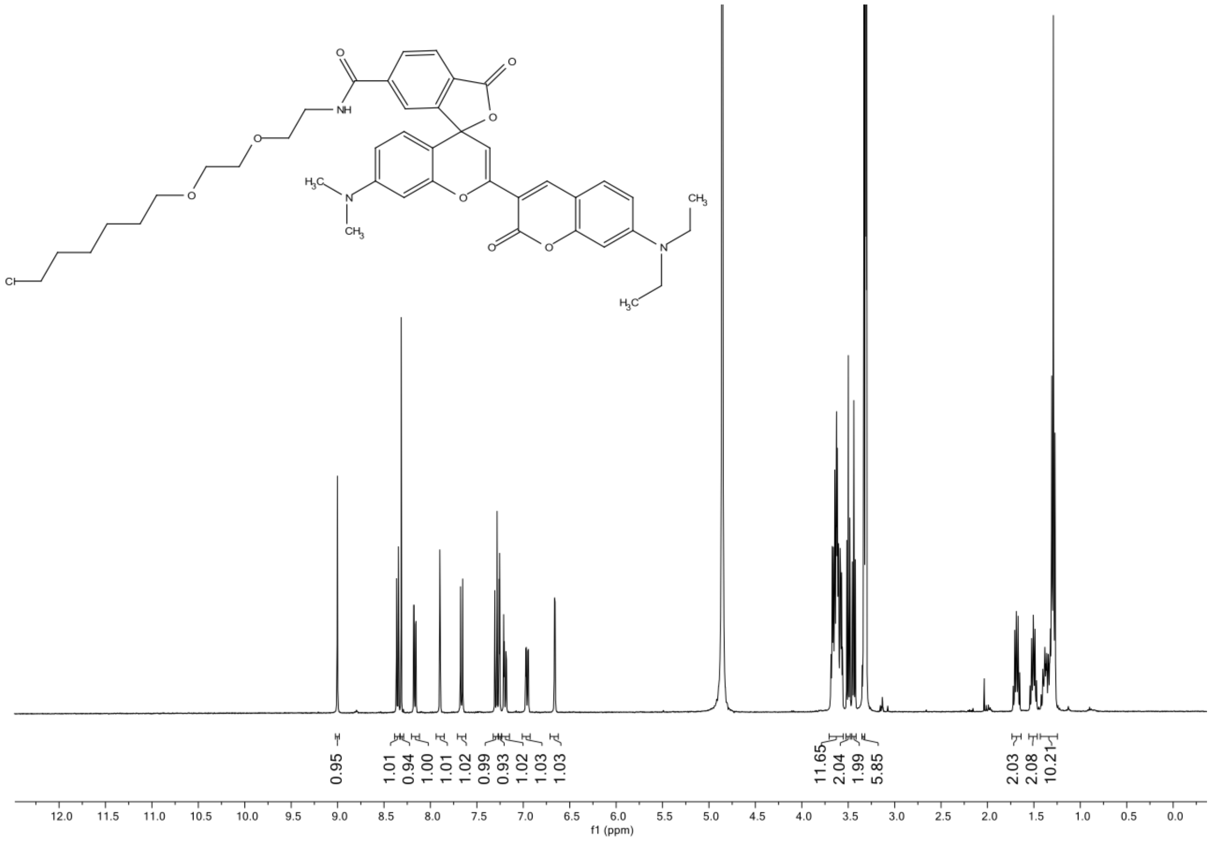
0.95
1.01
0.94
1.00
1.01
1.02
0.99
0.93
1.02
1.03
1.03
11.65
2.04
1.99
5.85
2.03
2.08
10.21
f1 (ppm)

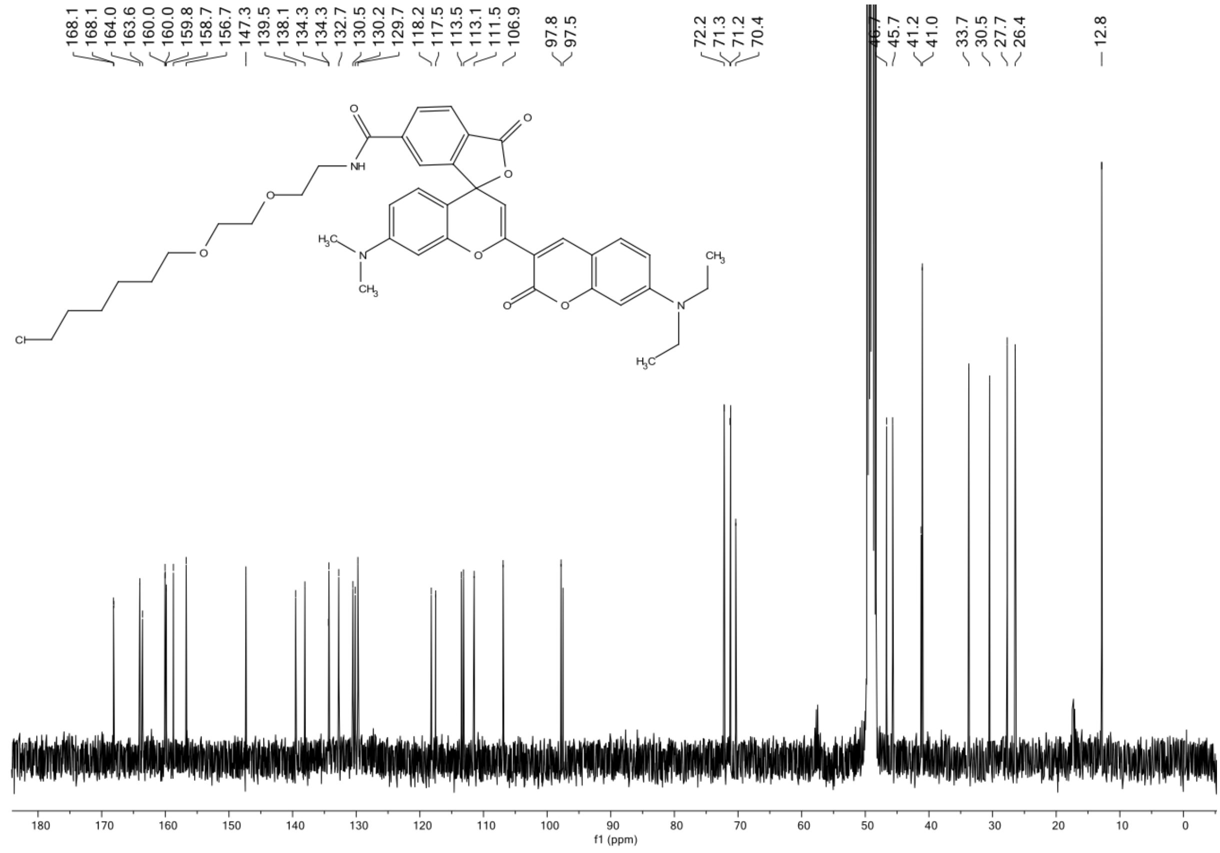
168.1
168.1
164.0
163.6
160.0
160.0
159.8
158.7
156.7
147.3
139.5
138.1
134.3
134.3
132.7
130.5
130.2
129.7
118.2
117.5
113.5
113.1
111.5
106.9
97.8
97.5
72.2
71.3
71.2
70.4
45.7
41.2
41.0
33.7
30.5
27.7
26.4
12.8
f1 (ppm)

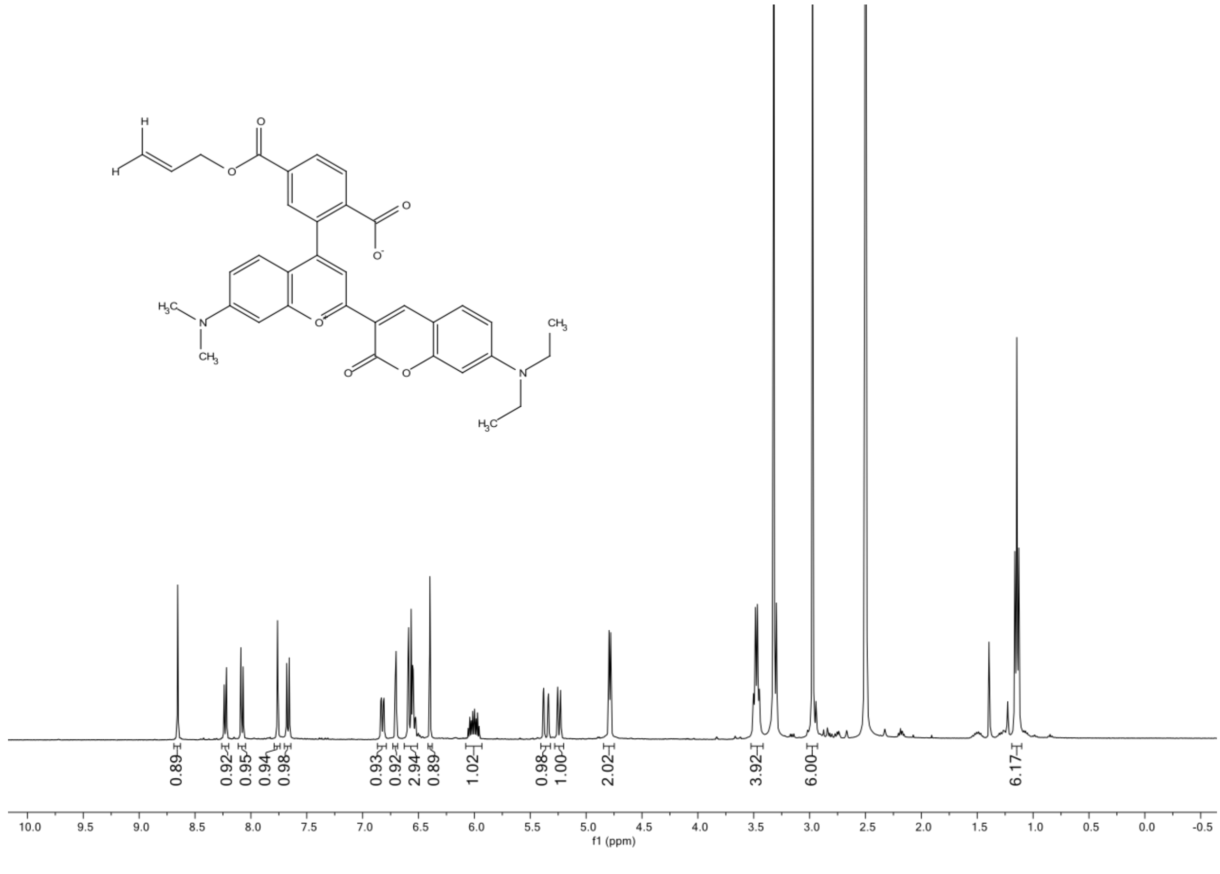
0.89
0.92
0.95
0.94
0.98
0.93
0.92
2.94
0.89
1.02
0.98
1.00
2.02
3.92
6.00
6.17
f1 (ppm)

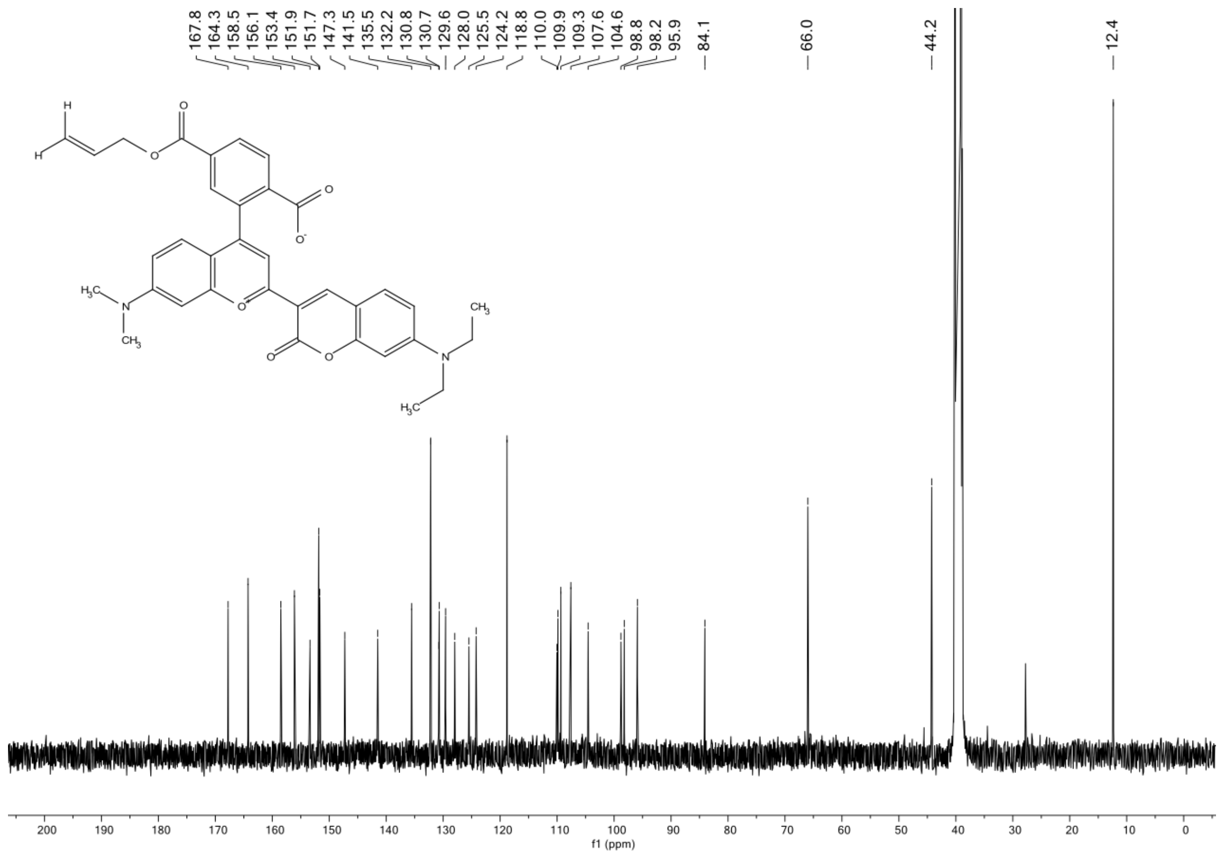
167.8
164.3
158.5
156.1
153.4
151.9
151.7
147.3
141.5
135.5
132.2
130.8
130.7
129.6
128.0
125.5
124.2
118.8
110.0
109.9
109.3
107.6
104.6
98.8
98.2
95.9
84.1
66.0
44.2
12.4
f1 (ppm)

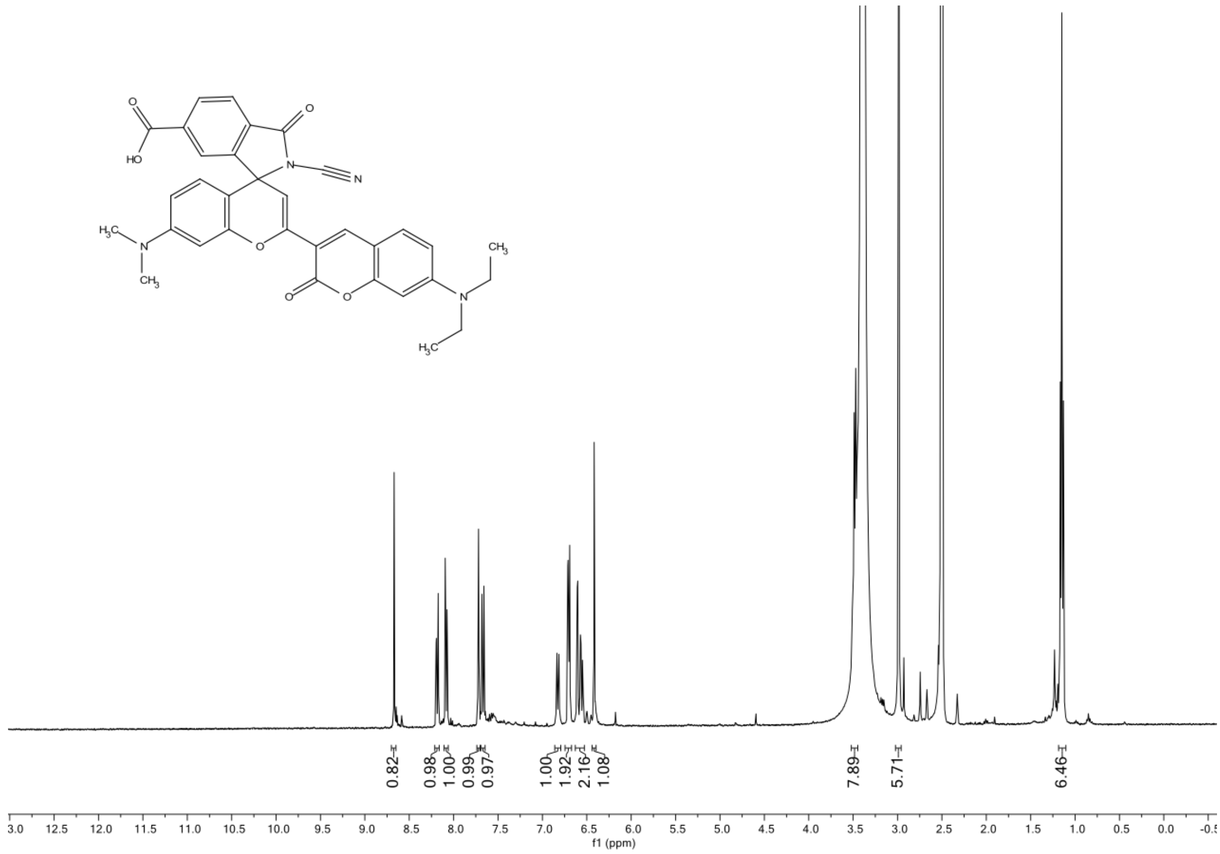
0.82
0.98
1.00
0.99
0.97
1.00
1.92
2.16
1.08
7.89
5.71
6.46
f1 (ppm)

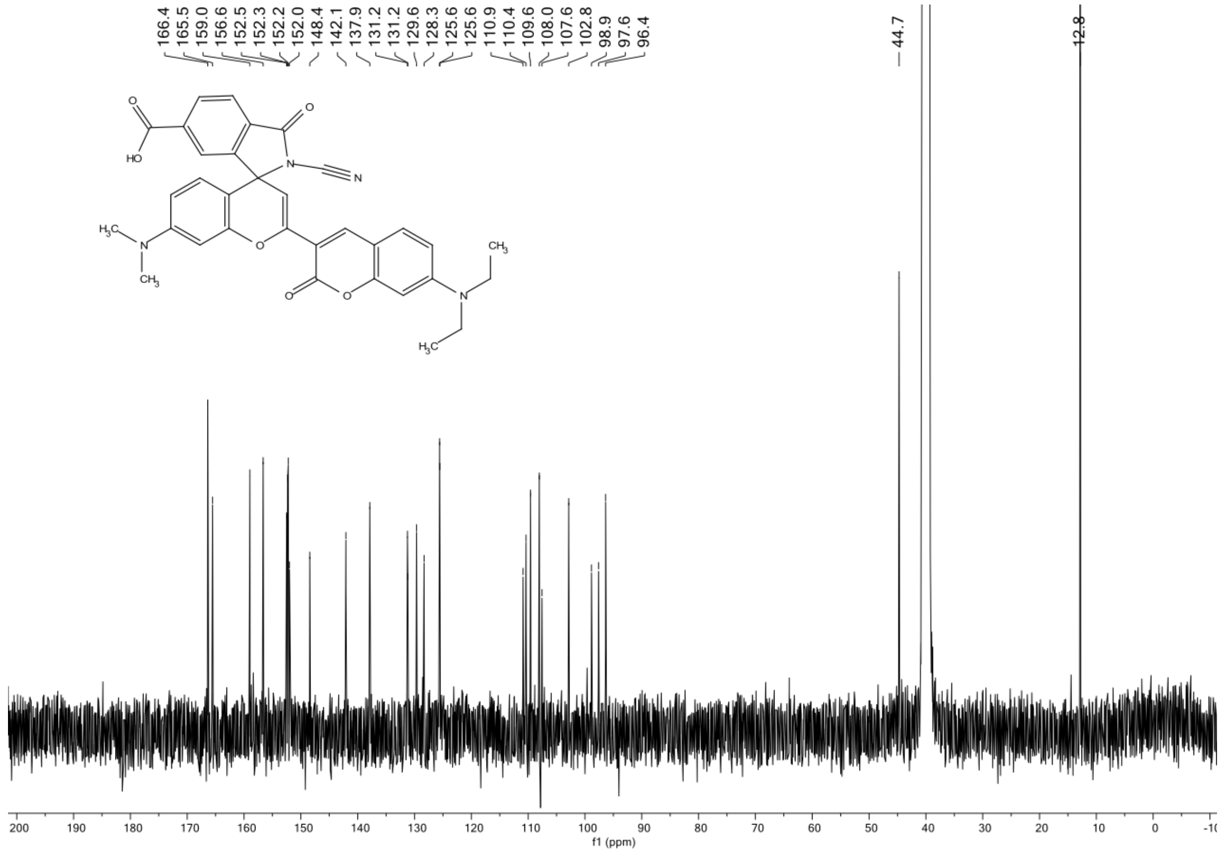
166.4
165.5
159.0
156.6
152.5
152.3
152.2
152.0
148.4
142.1
137.9
131.2
131.2
129.6
128.3
125.6
125.6
110.9
110.4
109.6
108.0
107.6
102.8
98.9
97.6
96.4
44.7
12.8
f1 (ppm)

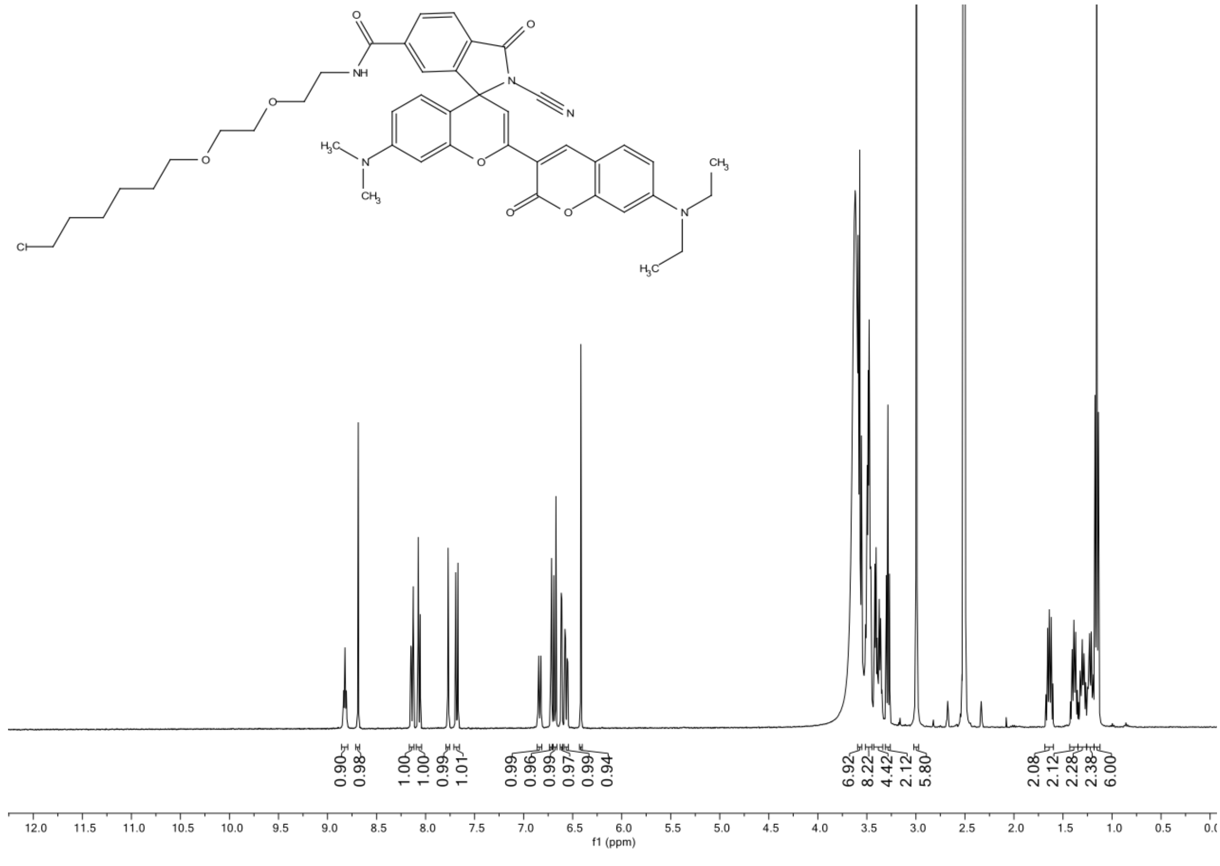
0.90
0.98
1.00
1.00
0.99
1.01
0.99
0.96
0.99
0.97
0.99
0.94
6.92
8.22
4.42
2.12
5.80
2.08
2.12
2.28
2.38
6.00
f1 (ppm)

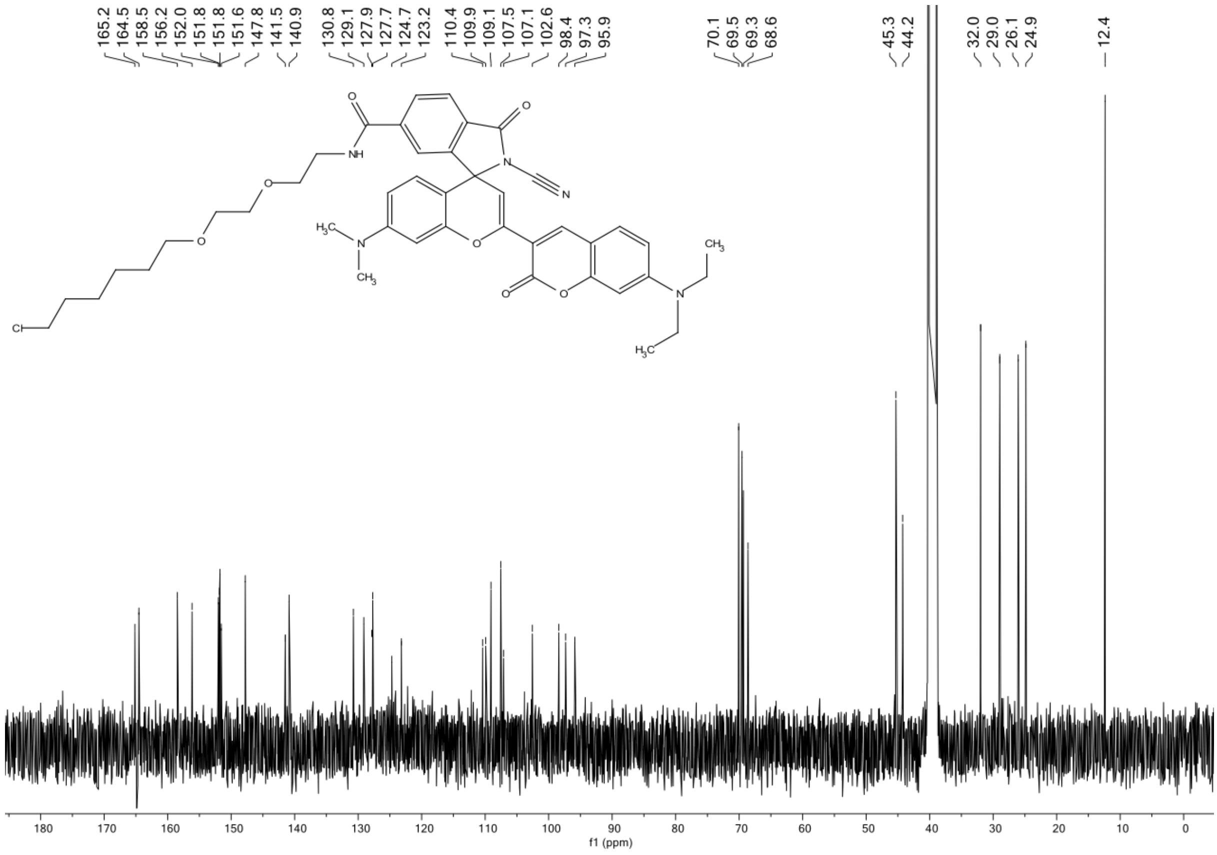
165.2
164.5
158.5
156.2
152.0
151.8
151.8
151.6
147.8
141.5
140.9
130.8
129.1
127.9
127.7
124.7
123.2
110.4
109.9
109.1
107.5
107.1
102.6
98.4
97.3
95.9
70.1
69.5
69.3
68.6
45.3
44.2
32.0
29.0
26.1
24.9
12.4
Cl
O
NH
N
N
H_3C
CH_3
CH_3
H_3C
180 170 160 150 140 130 120 110 100 90 80 70 60 50 40 30 20 10 0
f1 (ppm)

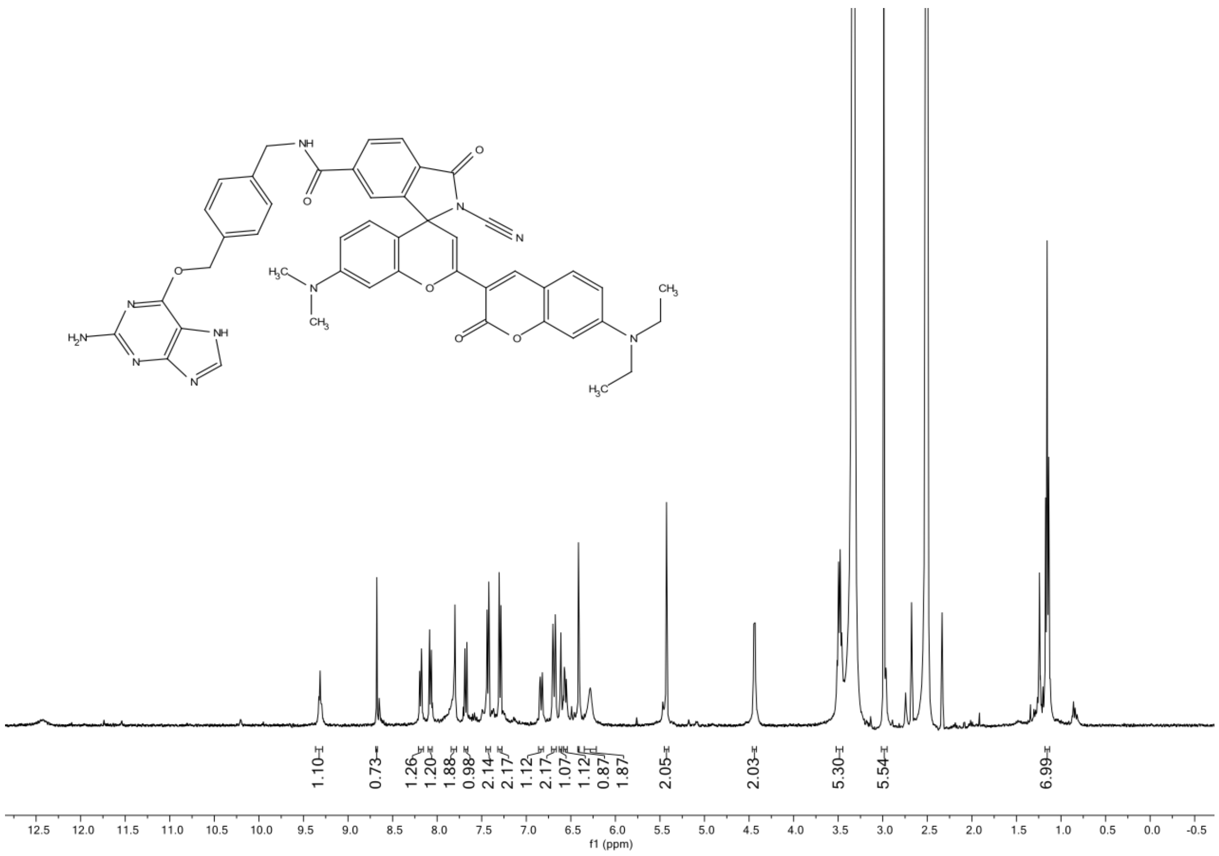
f1 (ppm)

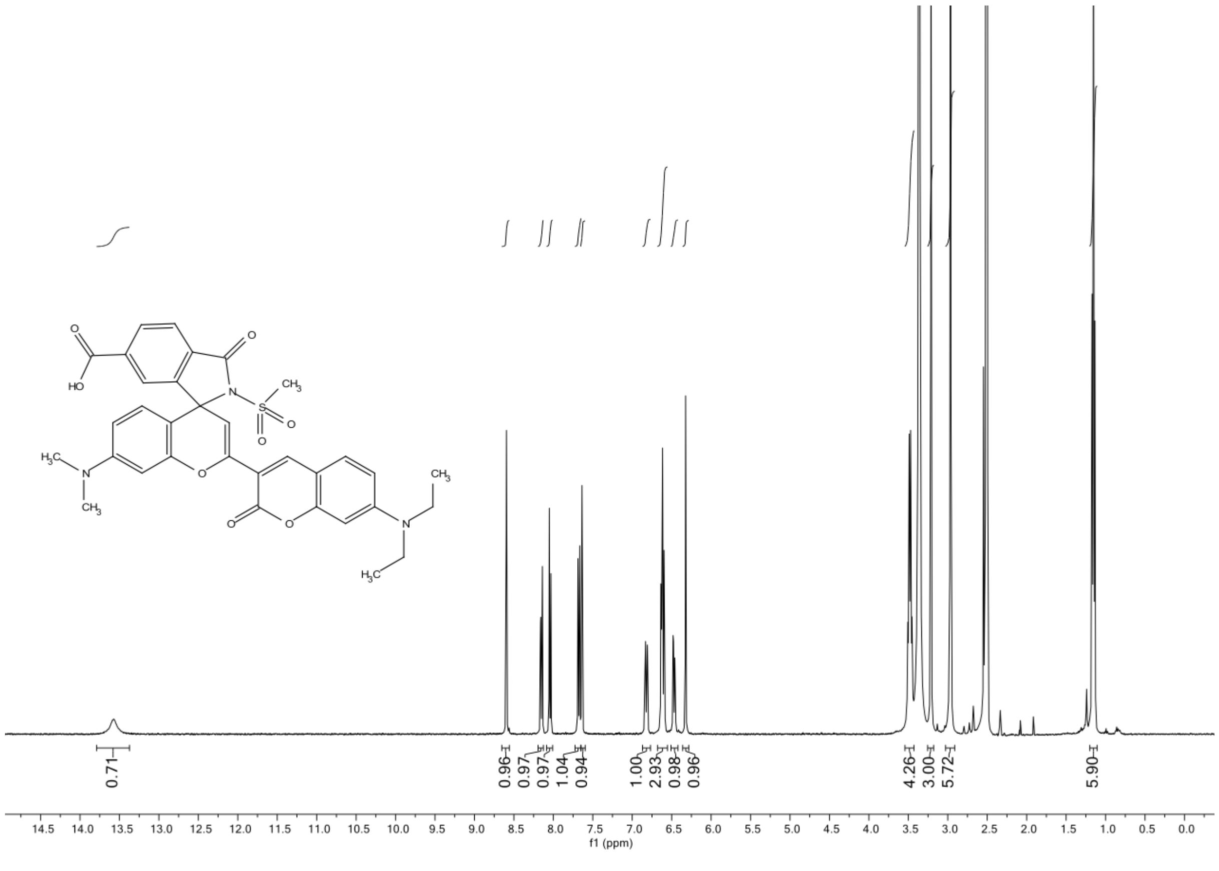
f1 (ppm)

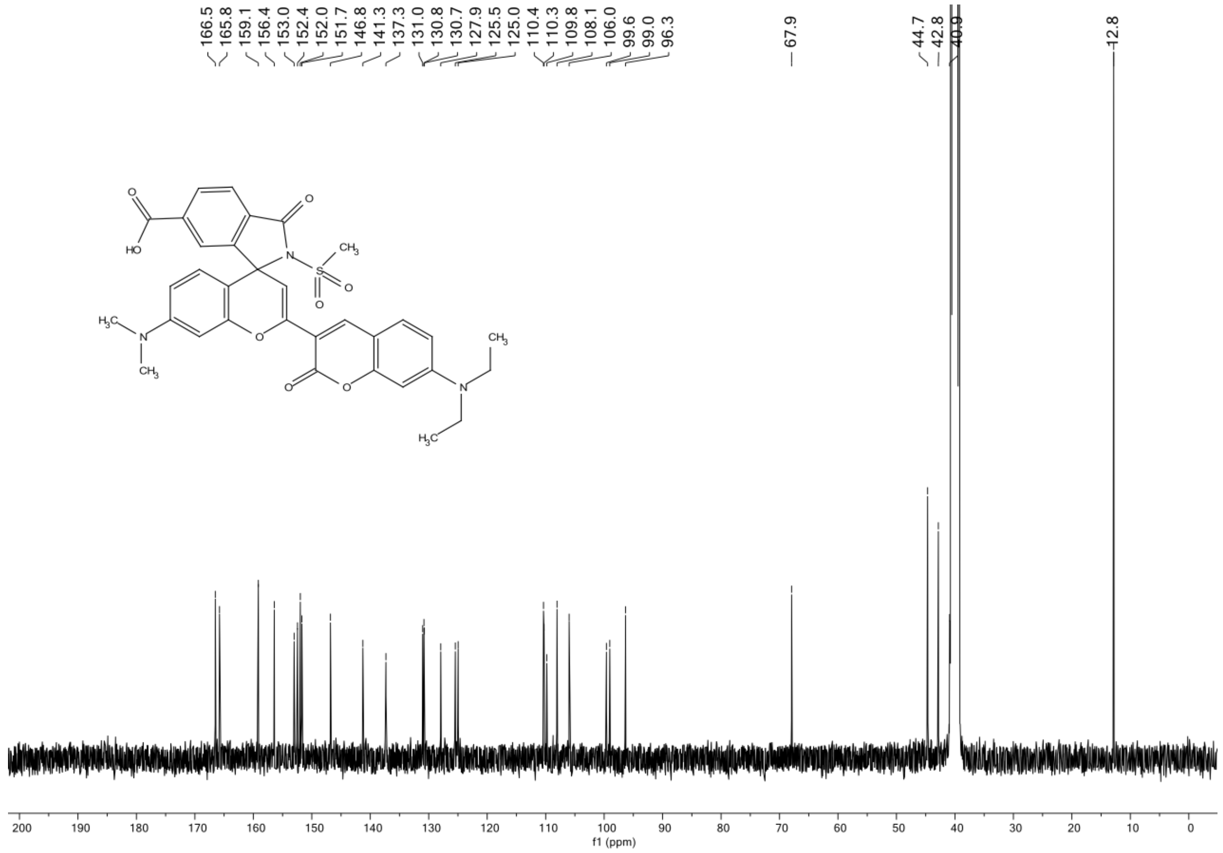

166.5
165.8
159.1
156.4
153.0
152.4
152.0
151.7
146.8
141.3
137.3
131.0
130.8
130.7
127.9
125.5
125.0
110.4
110.3
109.8
108.1
106.0
99.6
99.0
96.3
67.9
44.7
42.8
40.9
12.8
f1 (ppm)

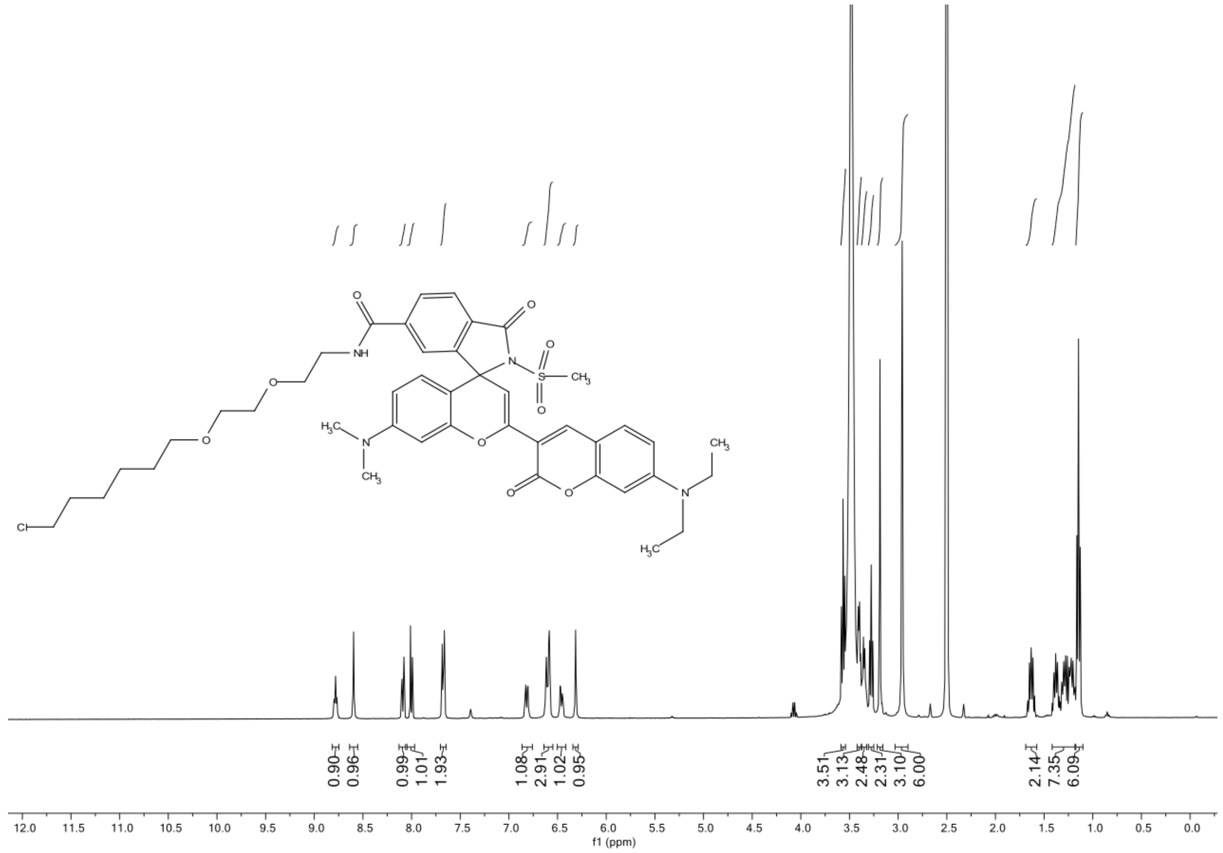

0.90
0.96
0.99
1.01
1.93
1.08
2.91
1.02
0.95
3.51
3.13
2.48
2.31
3.10
6.00
2.14
7.35
6.09
f1 (ppm)

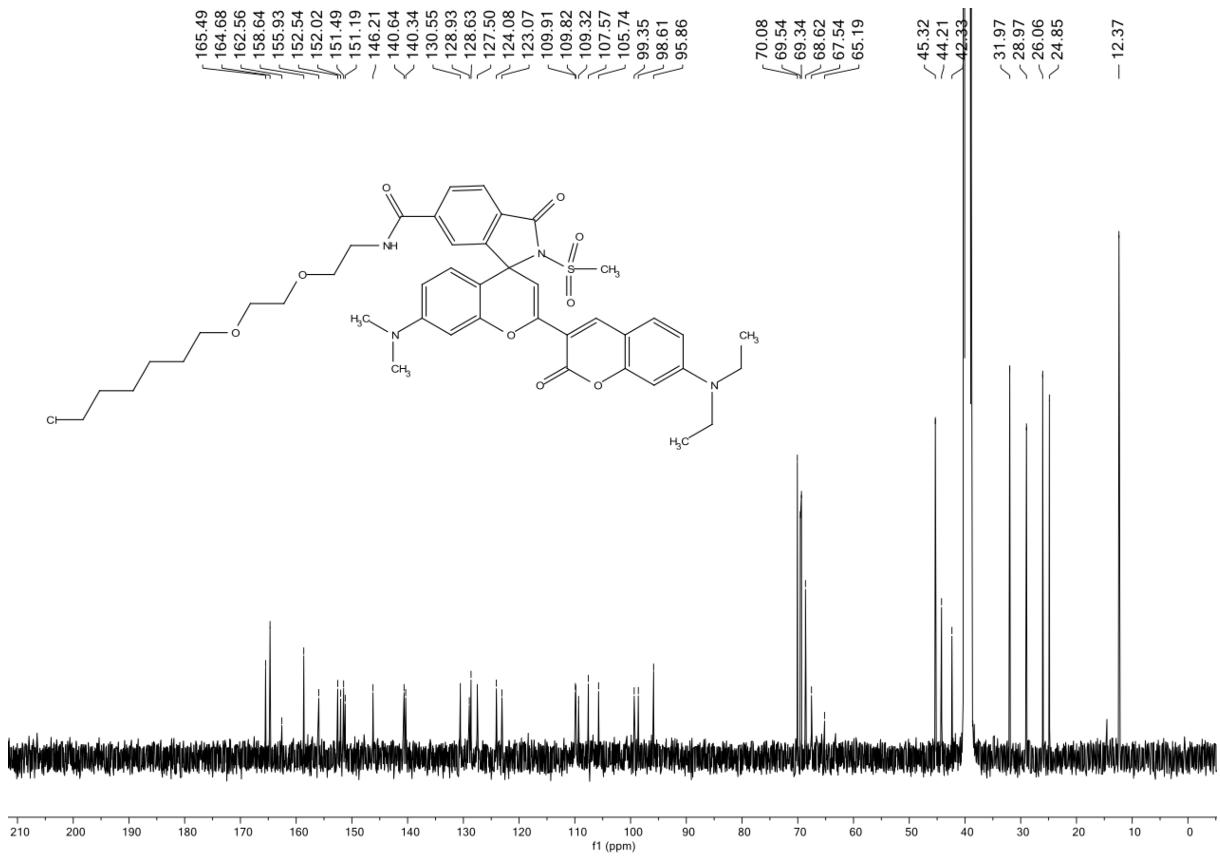
165.49
164.68
162.56
158.64
155.93
152.54
152.02
151.49
151.19
146.21
140.64
140.34
130.55
128.93
128.63
127.50
124.08
123.07
109.91
109.82
109.32
107.57
105.74
99.35
98.61
95.86
70.08
69.54
69.34
68.62
67.54
65.19
45.32
44.21
42.33
31.97
28.97
26.06
24.85
12.37
f1 (ppm)

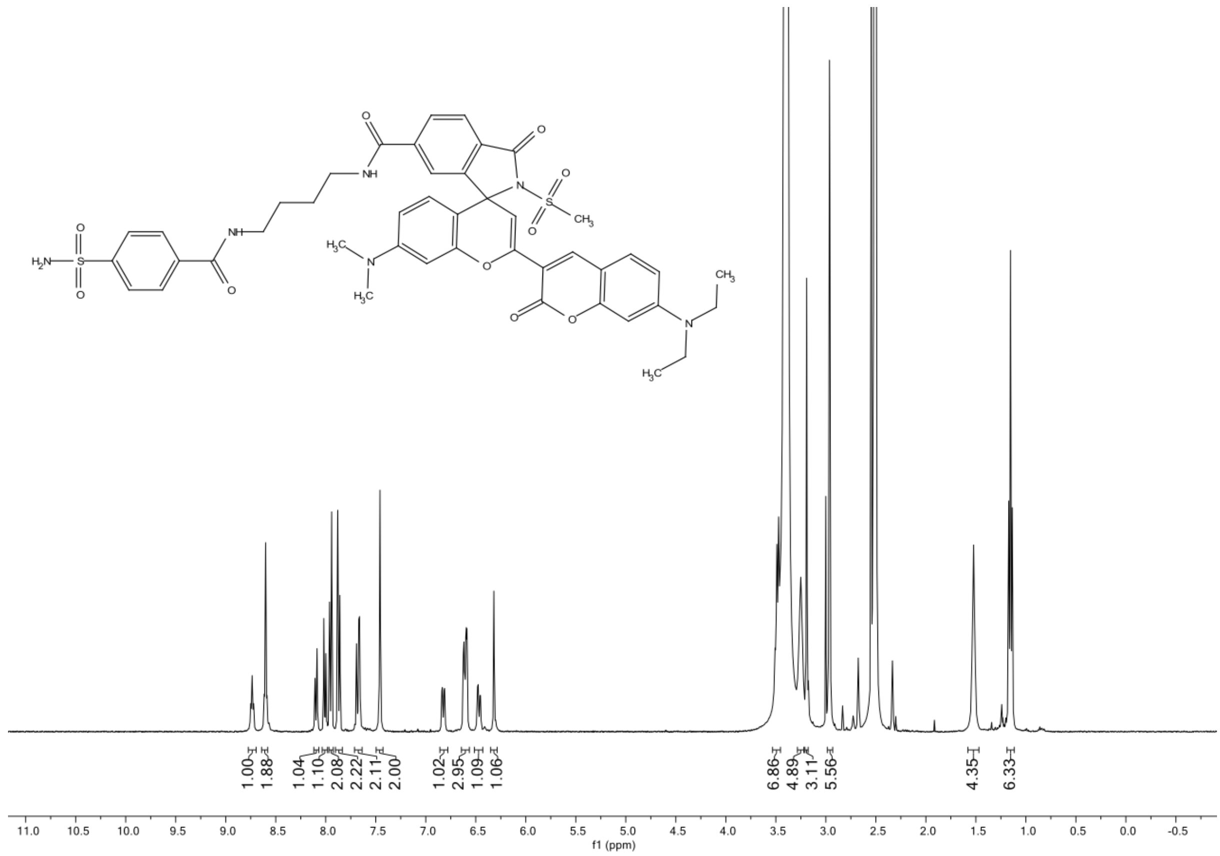
1.00
1.88
1.04
1.10
2.08
2.22
2.11
2.00
1.02
2.95
1.09
1.06
6.86
4.89
3.11
5.56
4.35
6.33
f1 (ppm)

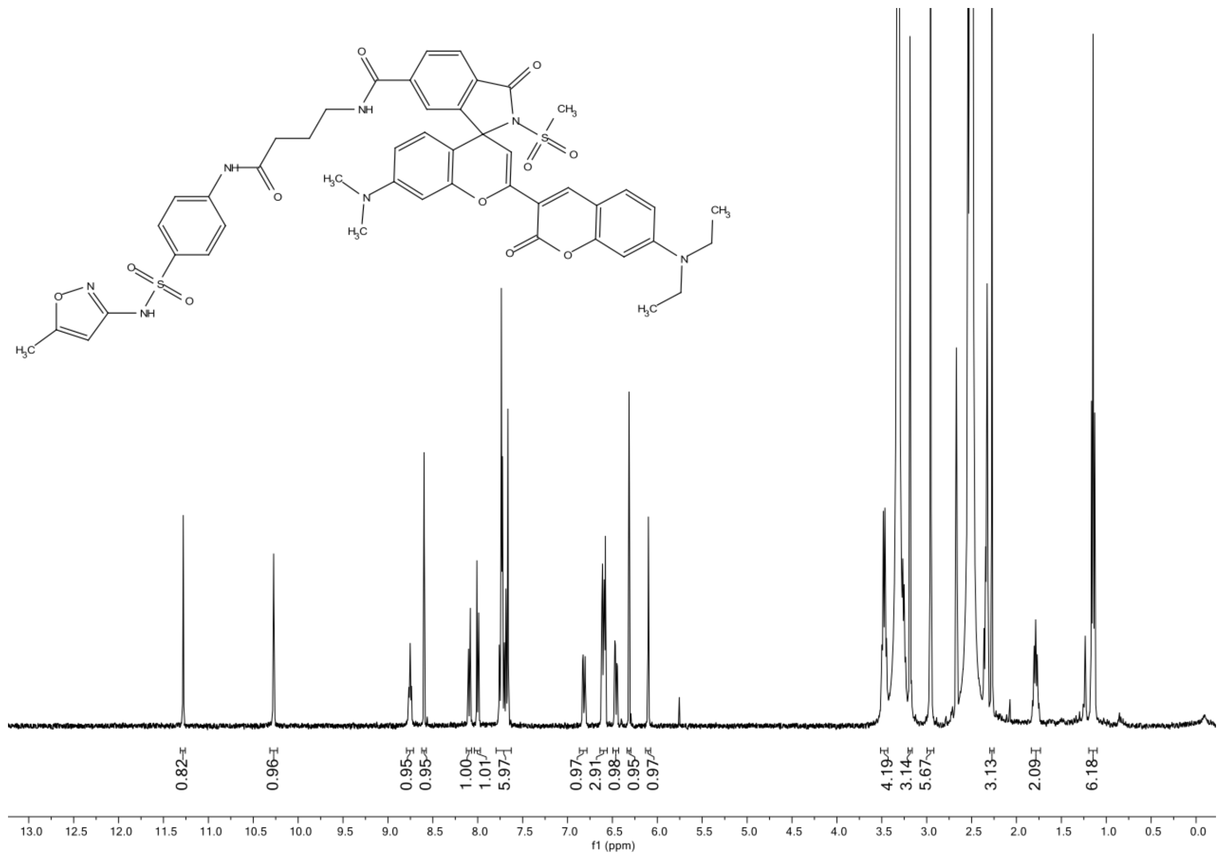
0.82
0.96
0.95
0.95
1.00
1.01
5.97
0.97
2.91
0.98
0.95
0.97
4.19
3.14
5.67
3.13
2.09
6.18
f1 (ppm)

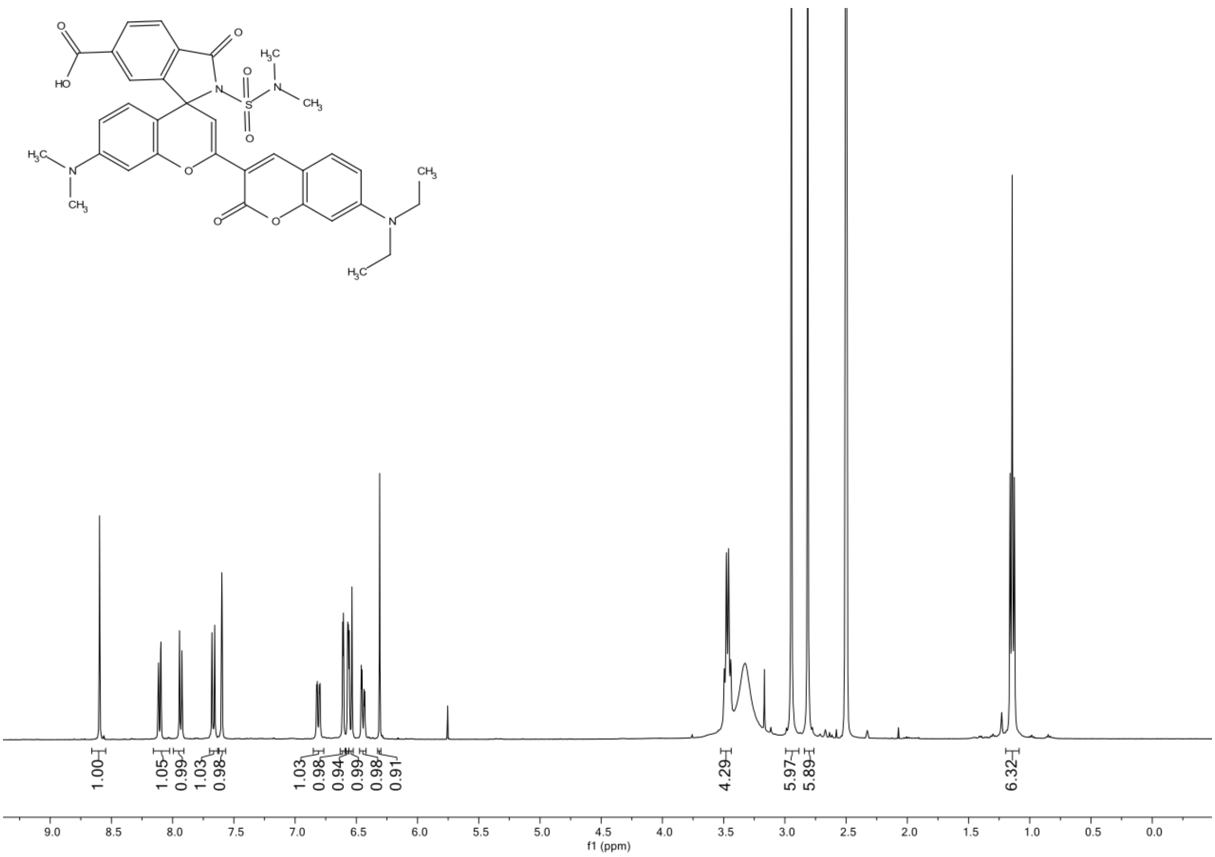

1.00
1.05
0.99
1.03
0.98
1.03
0.98
0.94
0.99
0.98
0.91
4.29
5.97
5.89
6.32
9.0 8.5 8.0 7.5 7.0 6.5 6.0 5.5 5.0 4.5 4.0 3.5 3.0 2.5 2.0 1.5 1.0 0.5 0.0
f1 (ppm)

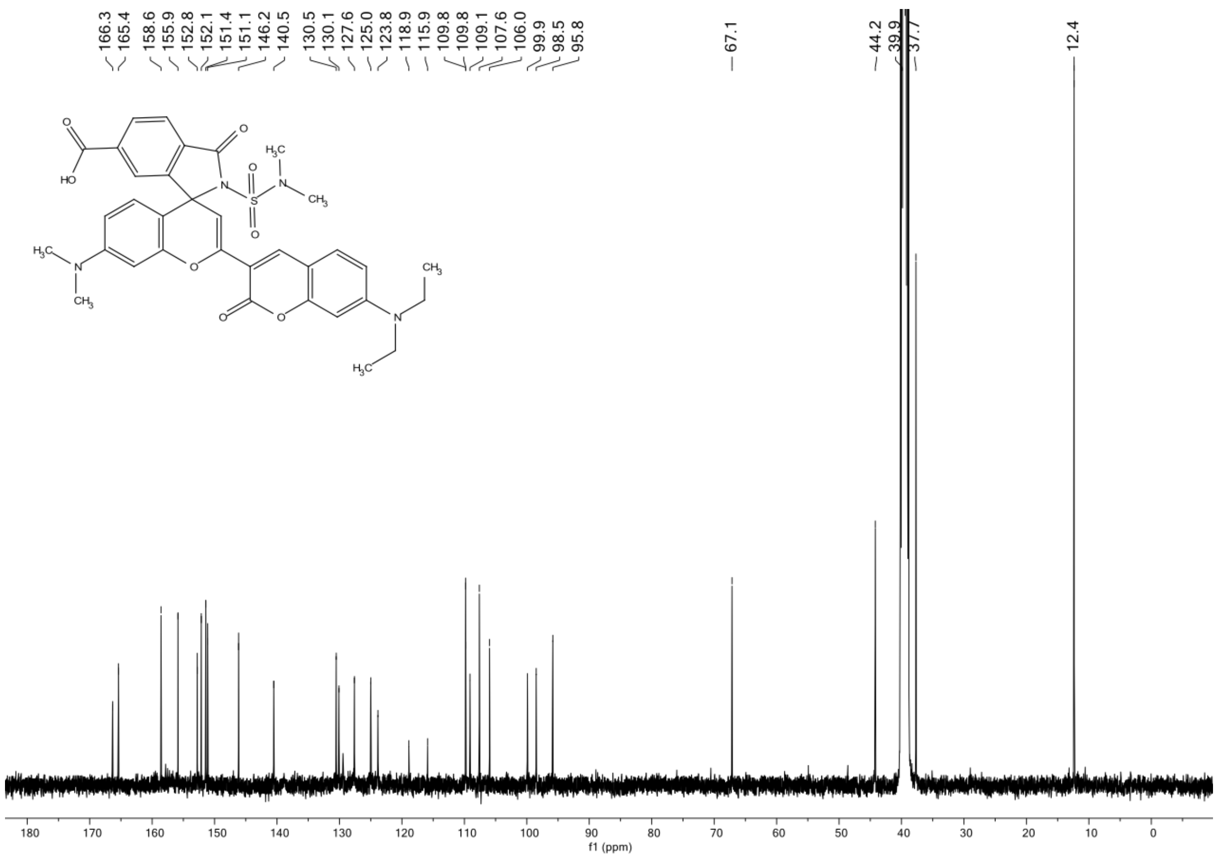

166.3
165.4
158.6
155.9
152.8
152.1
151.4
151.1
146.2
140.5
130.5
130.1
127.6
125.0
123.8
118.9
115.9
109.8
109.8
109.1
107.6
106.0
99.9
98.5
95.8
67.1
44.2
39.9
37.7
12.4
180 170 160 150 140 130 120 110 100 90 80 70 60 50 40 30 20 10 0
f1 (ppm)

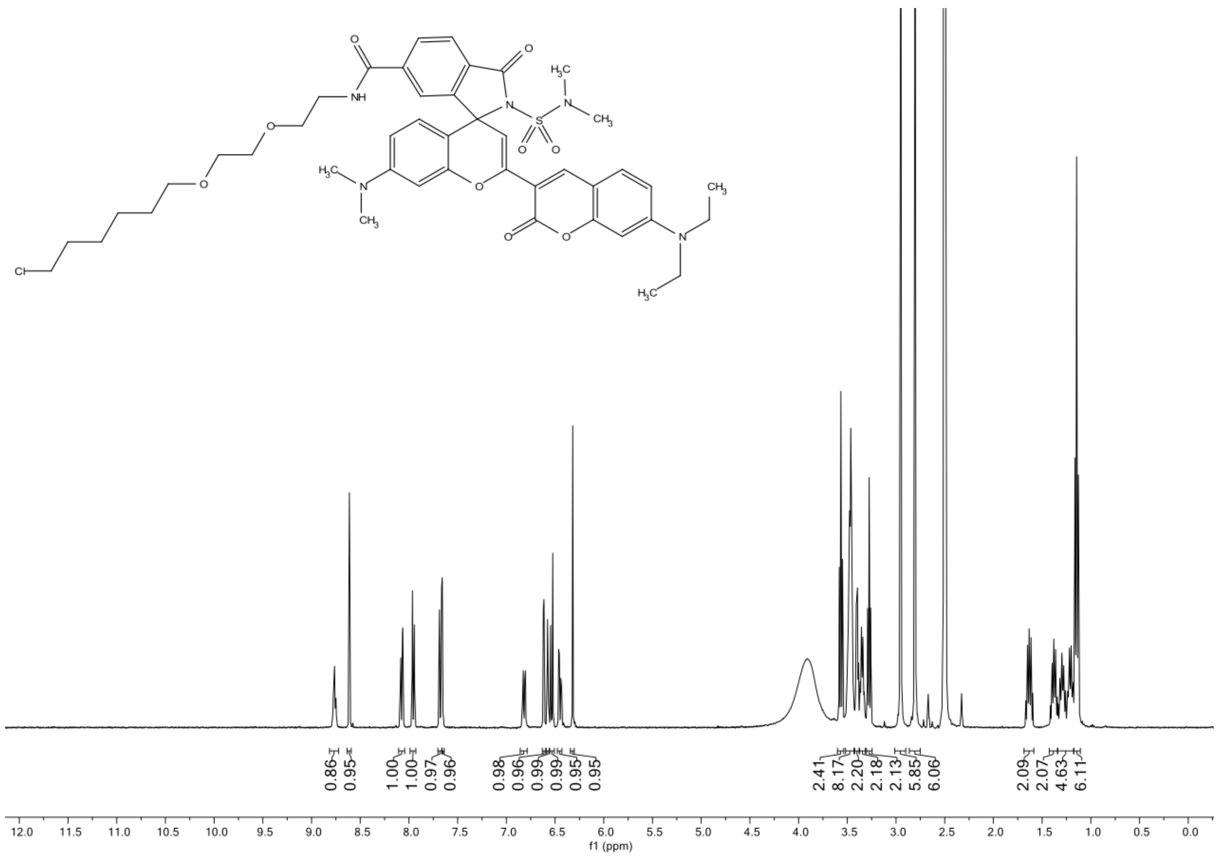

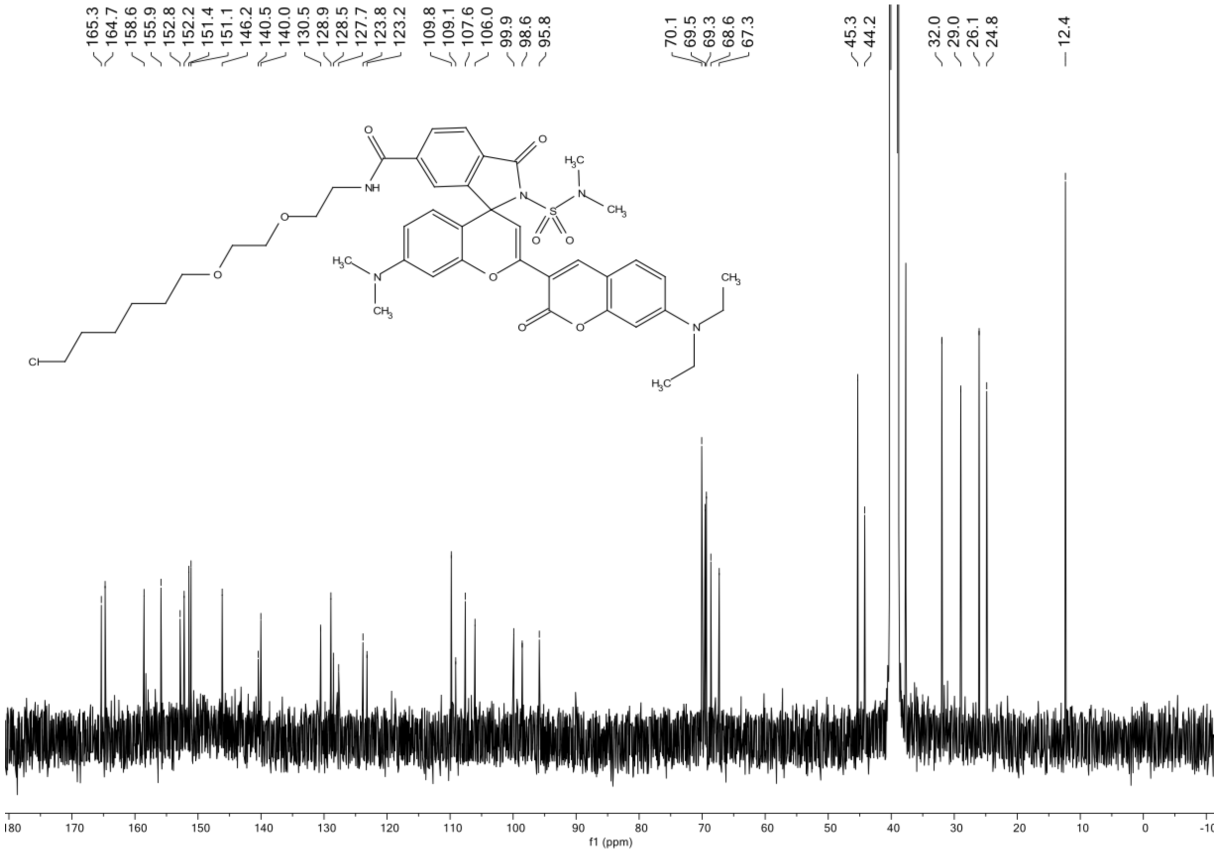

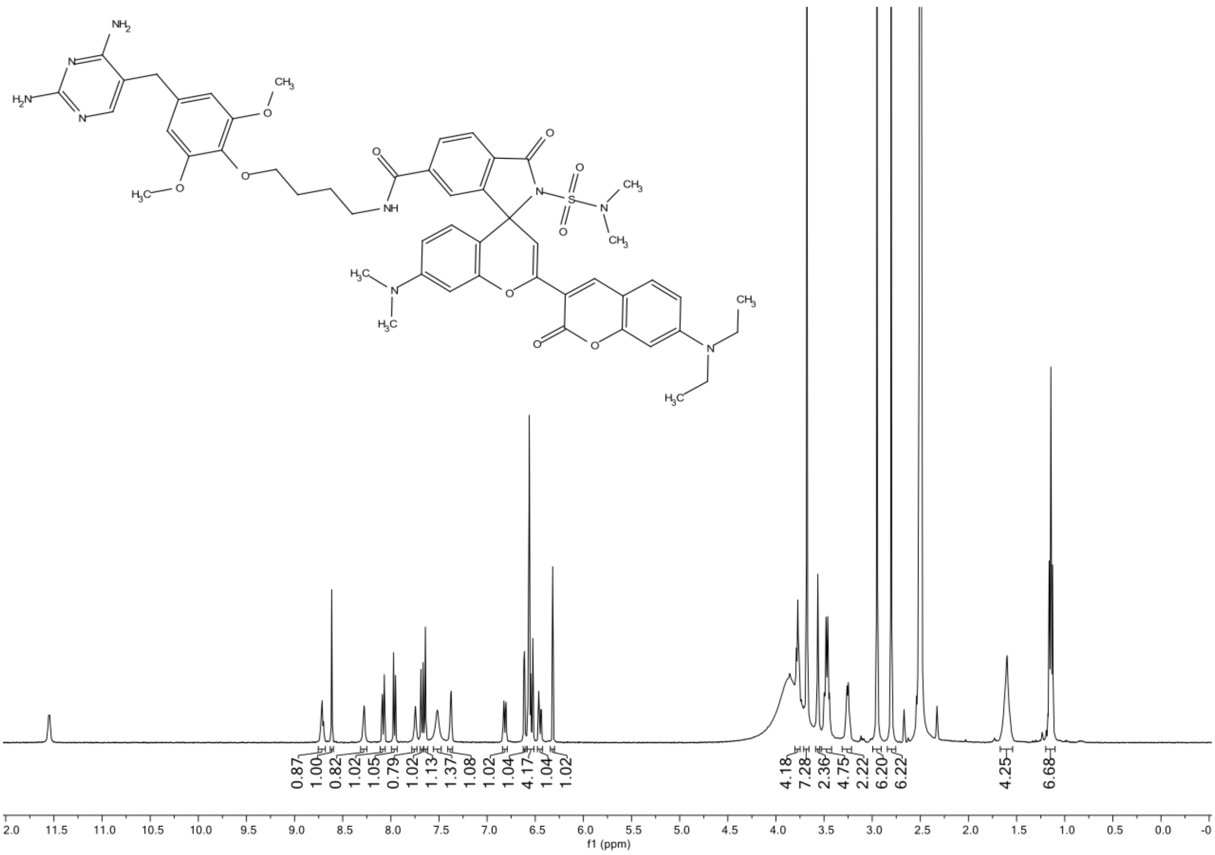

Supplementary HRMS spectra

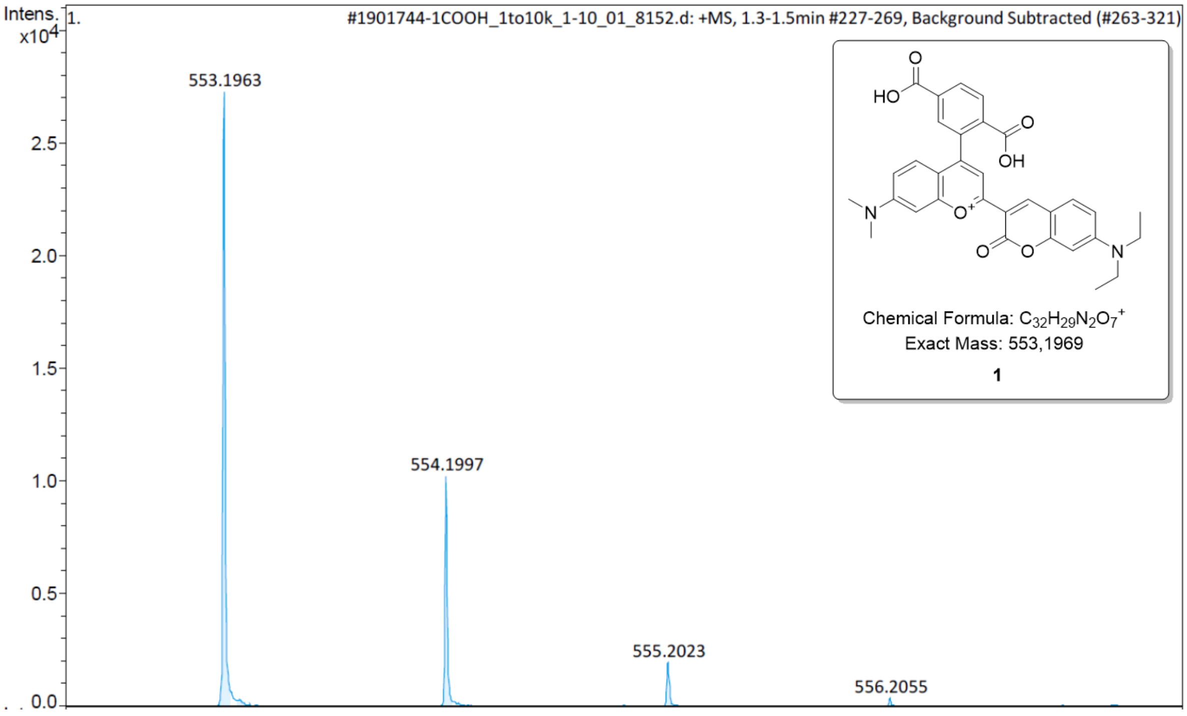

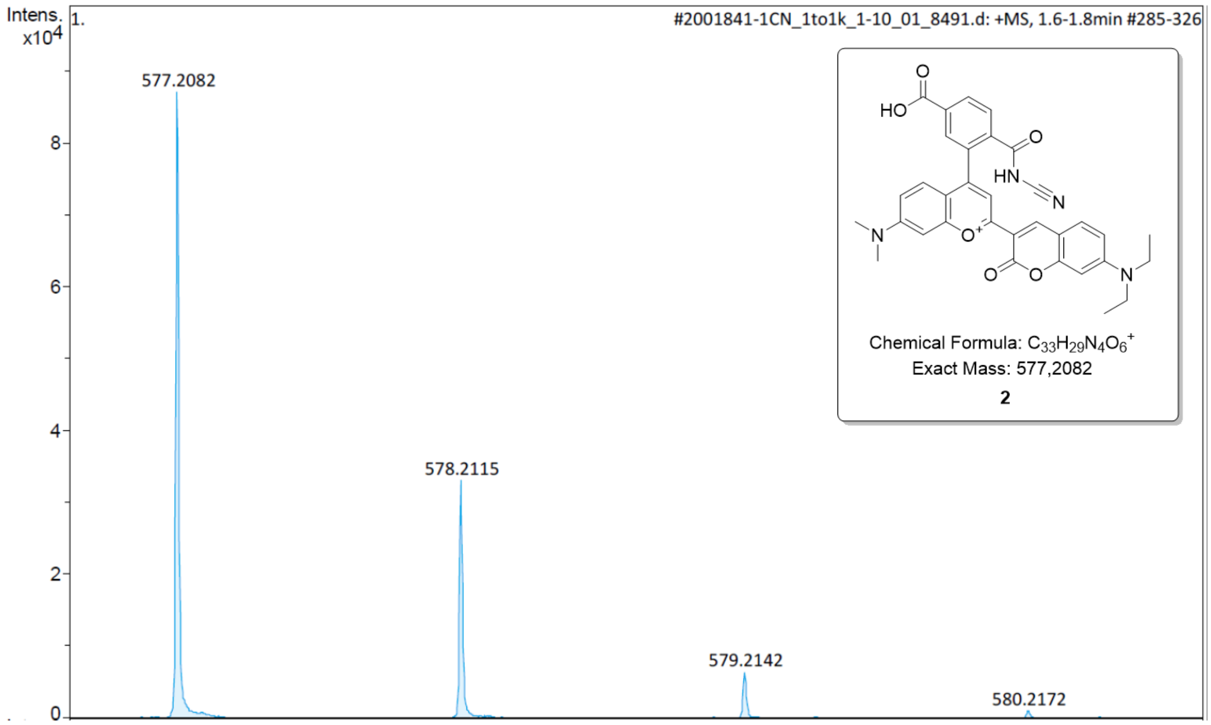
Intens.
x10^4
1.
#2001841-1CN_1to1k_1-10_01_8491.d: +MS, 1.6-1.8min #285-326
577.2082
578.2115
579.2142
580.2172
8
6
4
2
0
HO
O
HN
N
N
O+
O
O
N
Chemical Formula: C33H29N4O6+
Exact Mass: 577,2082
2

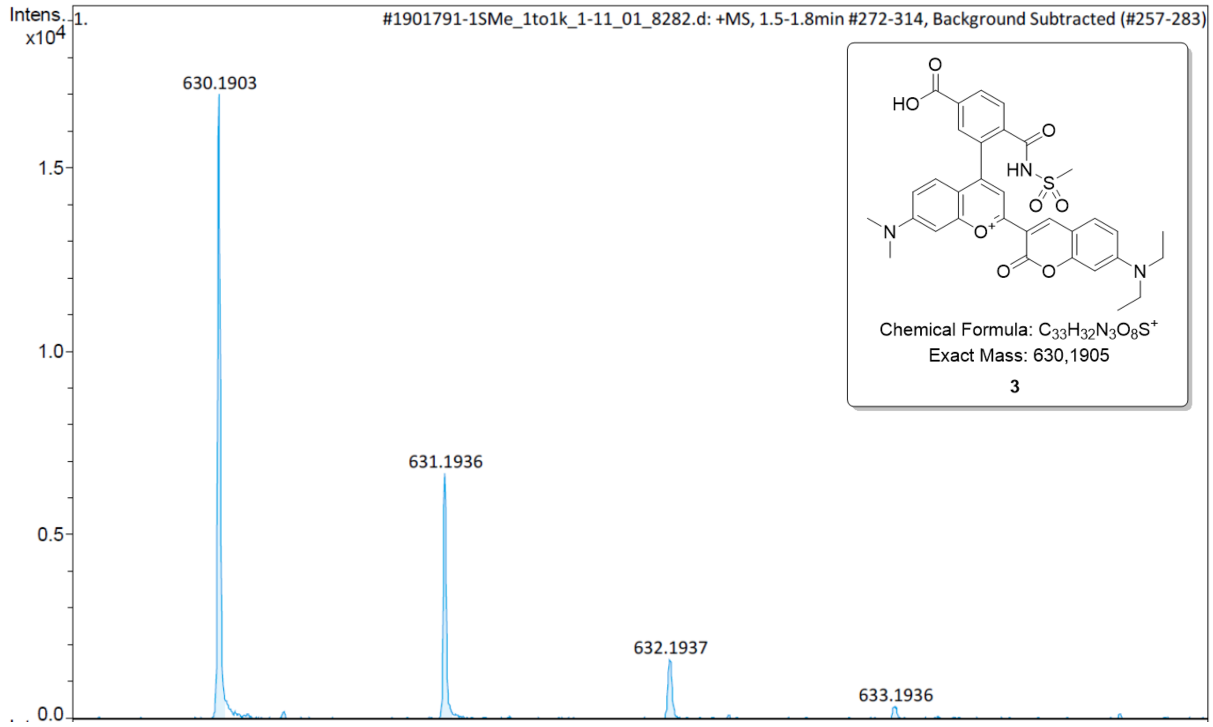
Intens.
x10^4
1.
#1901791-1SMe_1to1k_1-11_01_8282.d: +MS, 1.5-1.8min #272-314, Background Subtracted (#257-283)
630.1903
631.1936
632.1937
633.1936
1.5
1.0
0.5
0.0
HO
O
HN
S
O
O
N
O+
O
O
N
Chemical Formula: C33H32N3O8S+
Exact Mass: 630,1905
3

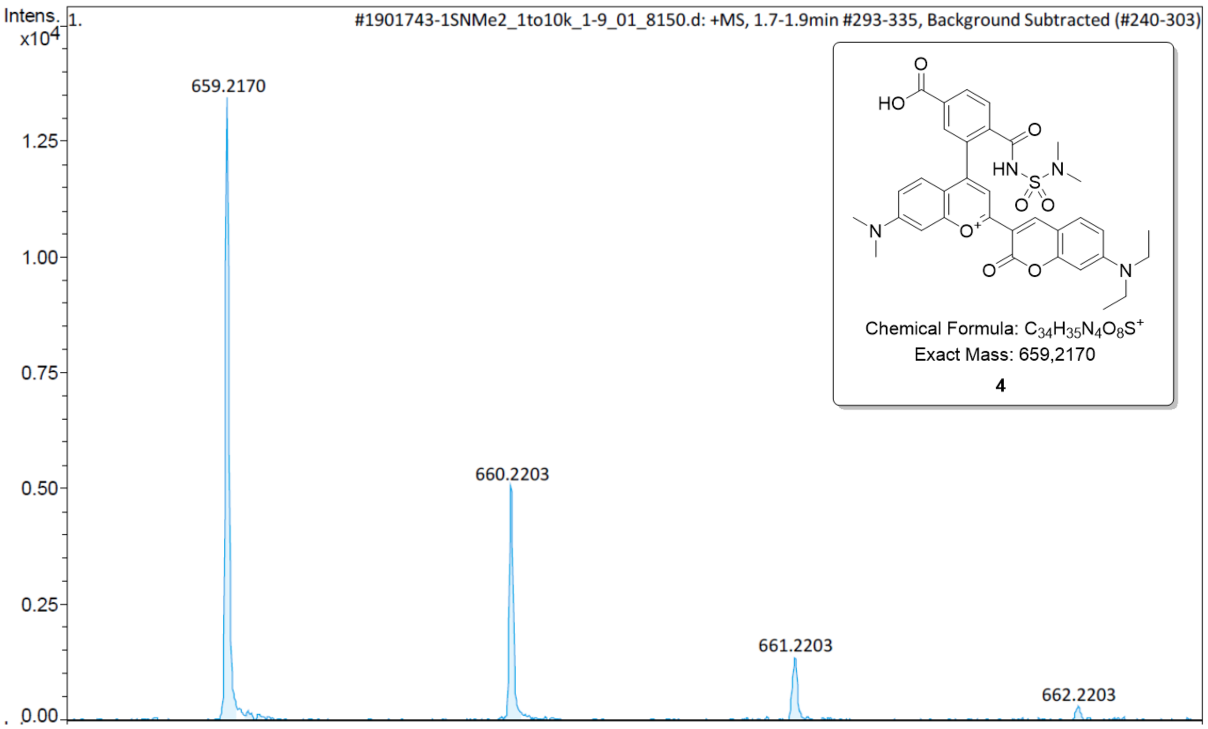
Intens.
x10^4
1.
#1901743-1SNMe2_1to10k_1-9_01_8150.d: +MS, 1.7-1.9min #293-335, Background Subtracted (#240-303)
1.25
1.00
0.75
0.50
0.25
0.00
659.2170
660.2203
661.2203
662.2203
HO
O
HN
S
N
O
N
O+
Chemical Formula: C34H35N4O8S+
Exact Mass: 659,2170
4

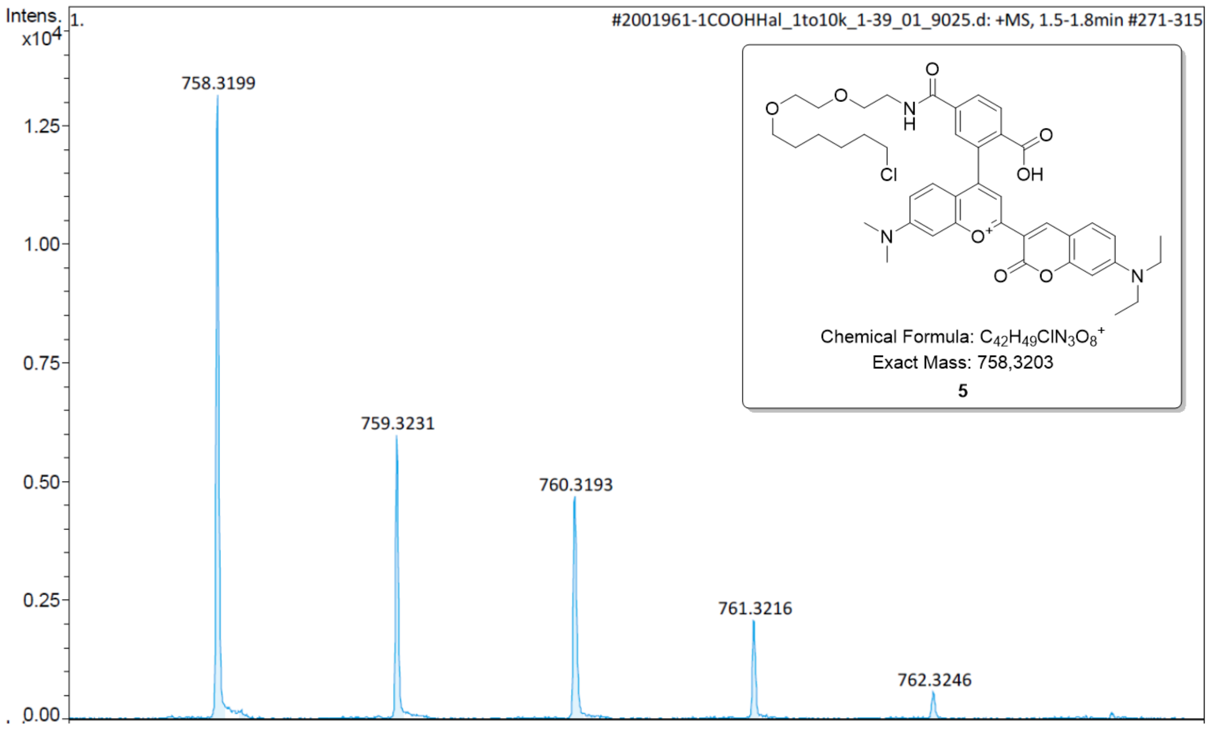
Intens.
x10^4
1.
#2001961-1COOHHal_1to10k_1-39_01_9025.d: +MS, 1.5-1.8min #271-315
1.25
1.00
0.75
0.50
0.25
0.00
758.3199
759.3231
760.3193
761.3216
762.3246
O
N
H
Cl
OH
N
O+
Chemical Formula: C42H49ClN3O8+
Exact Mass: 758,3203
5

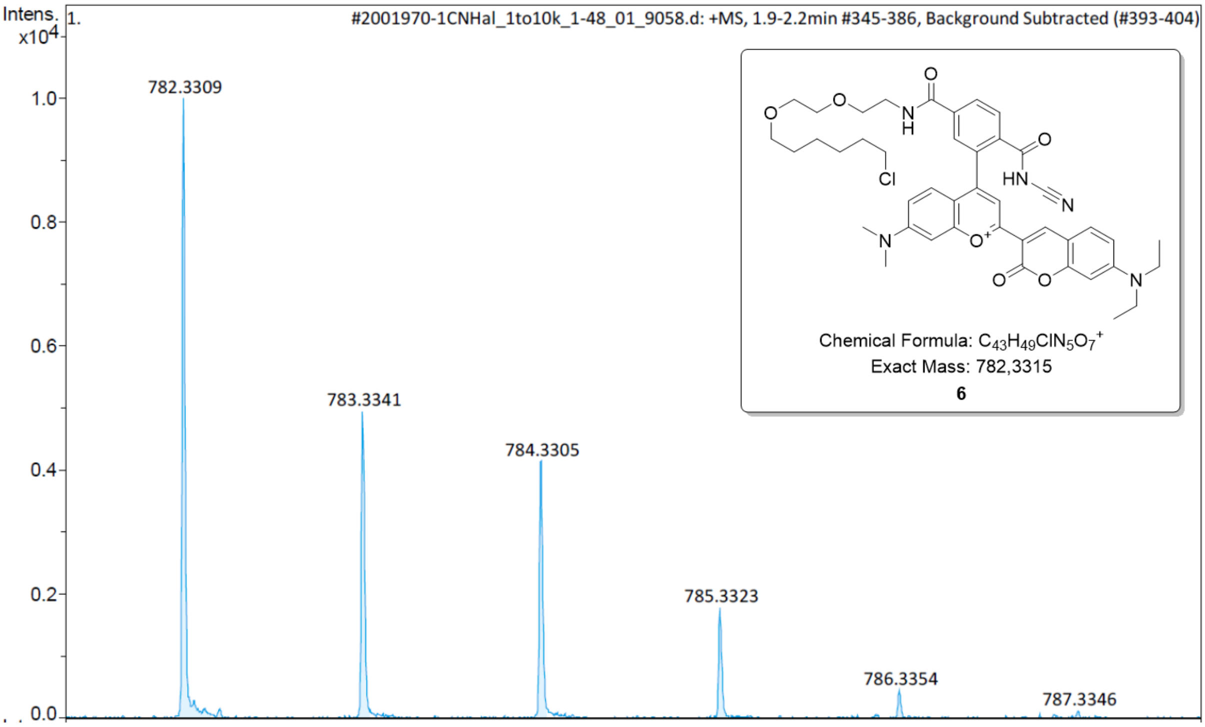
Intens.
x10^4
1.
#2001970-1CNHal_1to10k_1-48_01_9058.d: +MS, 1.9-2.2min #345-386, Background Subtracted (#393-404)
1.0
0.8
0.6
0.4
0.2
0.0
782.3309
783.3341
784.3305
785.3323
786.3354
787.3346
Cl
HN
N
O
Chemical Formula: C43H49ClN5O7+
Exact Mass: 782,3315
6

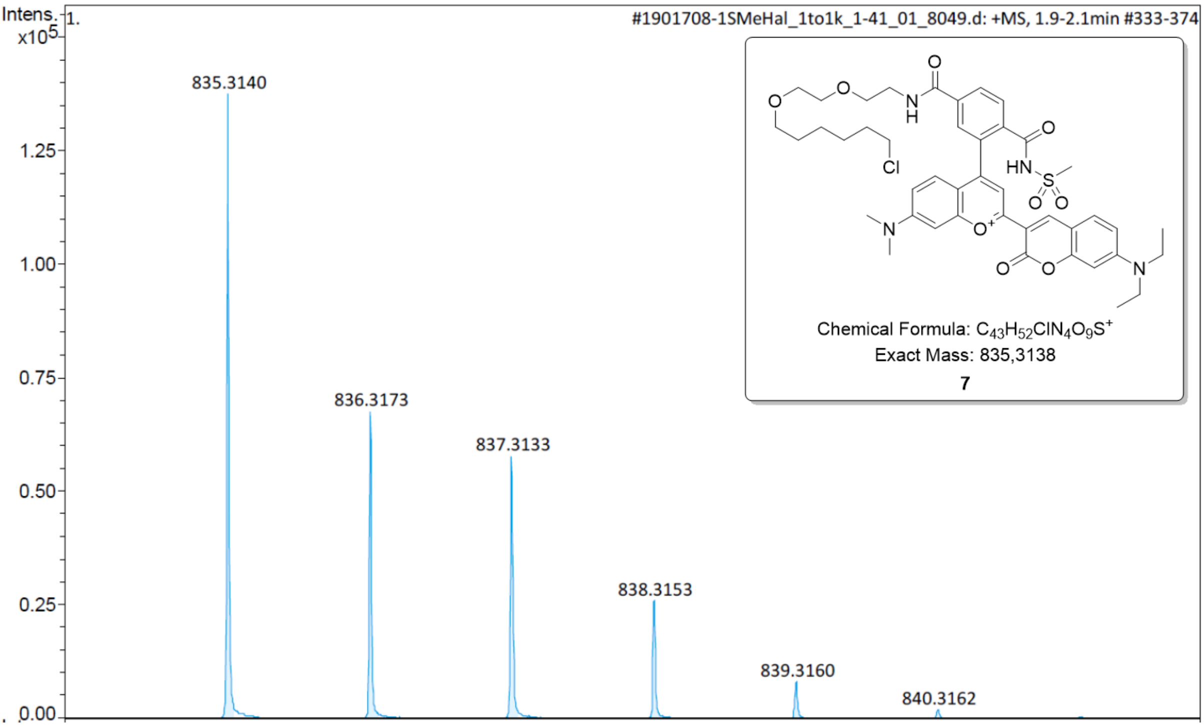
Intens.
x10^5
1.
#1901708-1SMeHal_1to1k_1-41_01_8049.d: +MS, 1.9-2.1min #333-374
1.25
1.00
0.75
0.50
0.25
0.00
835.3140
836.3173
837.3133
838.3153
839.3160
840.3162
Cl
HN
S
O
Chemical Formula: C43H52ClN4O9S+
Exact Mass: 835,3138
7

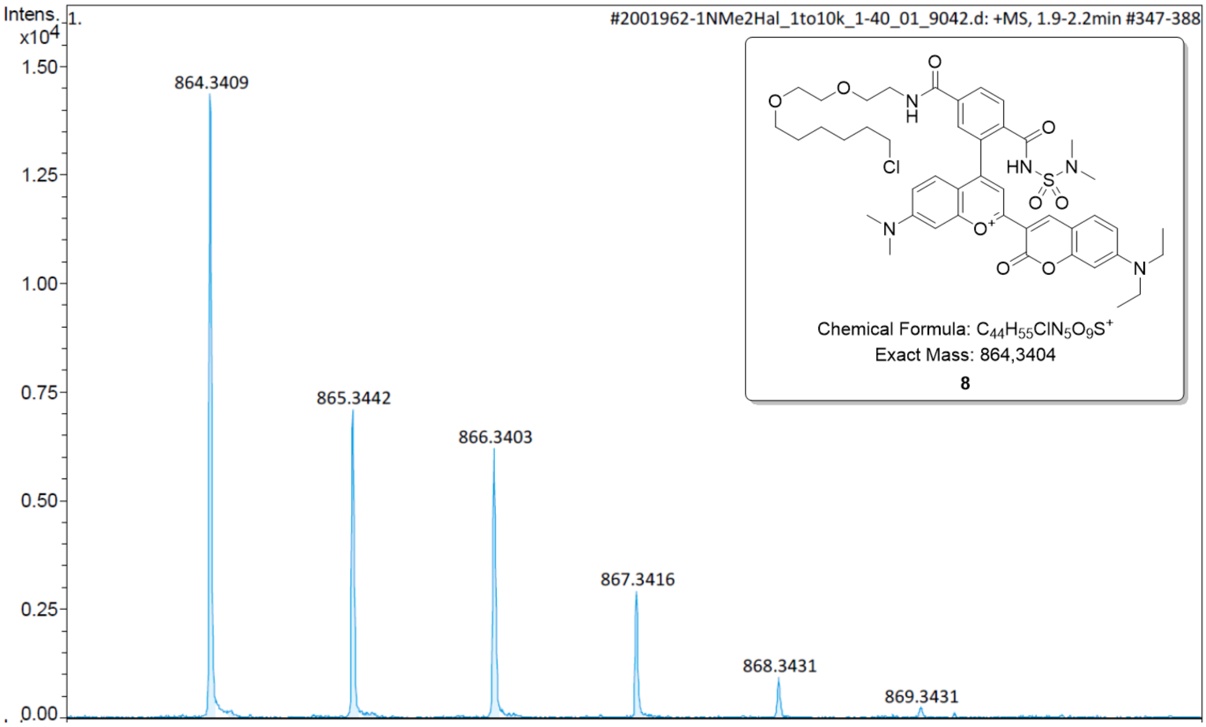

Intens.
x10^4
1.
#2001962-1NMe2Hal_1to10k_1-40_01_9042.d: +MS, 1.9-2.2min #347-388
1.50
1.25
1.00
0.75
0.50
0.25
0.00
864.3409
865.3442
866.3403
867.3416
868.3431
869.3431
Chemical Formula: $C_{44}H_{55}ClN_5O_9S^+$
Exact Mass: 864,3404
8

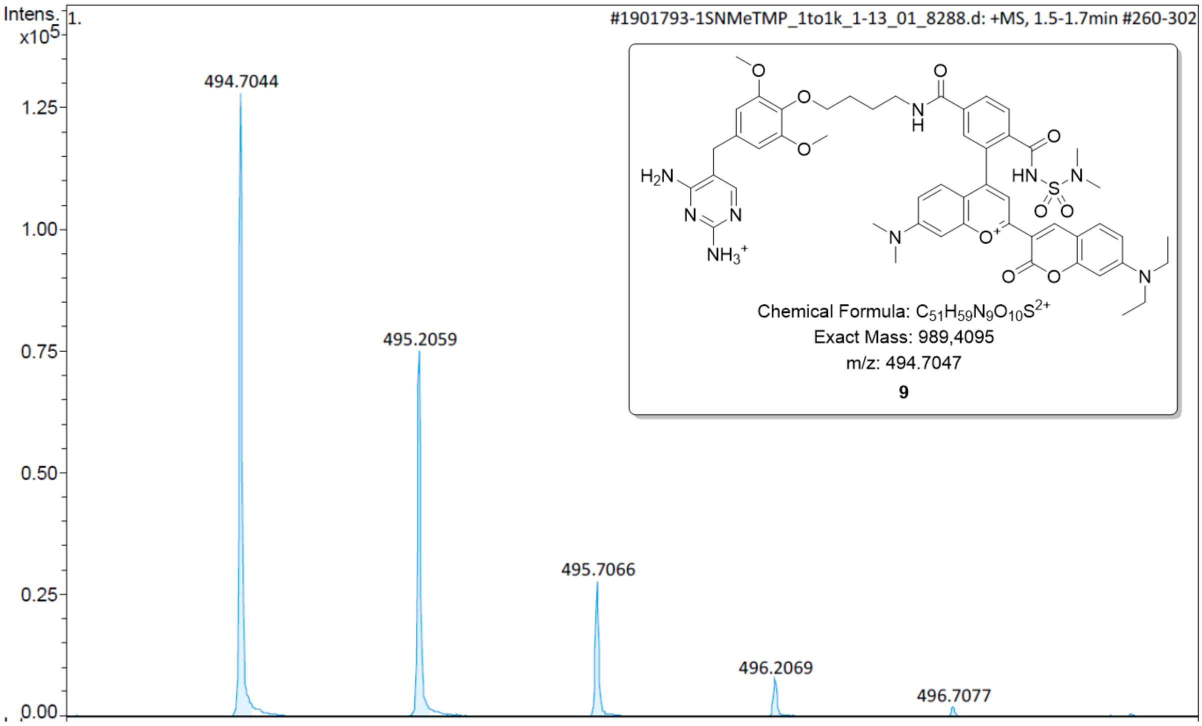

Intens.
x10^5
1.
#1901793-1SNMeTMP_1to1k_1-13_01_8288.d: +MS, 1.5-1.7min #260-302
1.25
1.00
0.75
0.50
0.25
0.00
494.7044
495.2059
495.7066
496.2069
496.7077
Chemical Formula: $C_{51}H_{59}N_9O_{10}S^{2+}$
Exact Mass: 989,4095
m/z: 494.7047
9

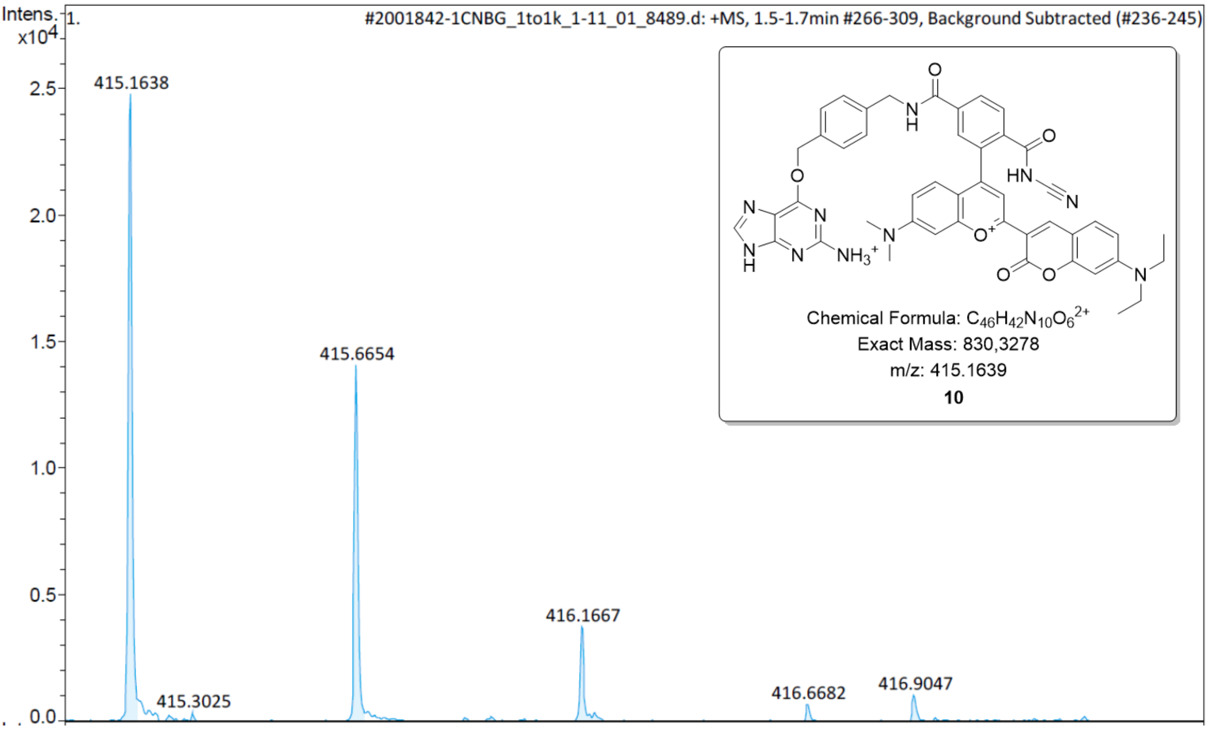
Intens.
x10^4
#2001842-1CNBG_1to1k_1-11_01_8489.d: +MS, 1.5-1.7min #266-309, Background Subtracted (#236-245)
415.1638
415.3025
415.6654
416.1667
416.6682
416.9047
Chemical Formula: C46H42N10O6 2+
Exact Mass: 830,3278
m/z: 415.1639
10

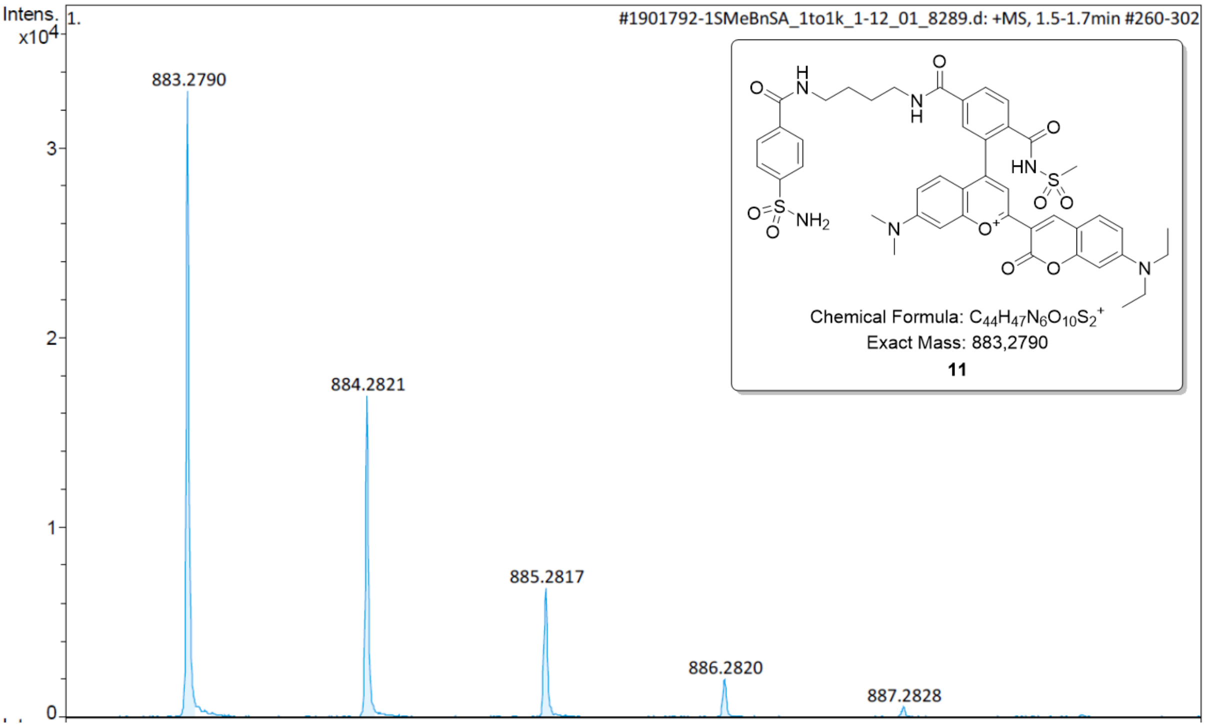
Intens.
x10^4
#1901792-1SMeBnSA_1to1k_1-12_01_8289.d: +MS, 1.5-1.7min #260-302
883.2790
884.2821
885.2817
886.2820
887.2828
Chemical Formula: C44H47N6O10S2 +
Exact Mass: 883,2790
11

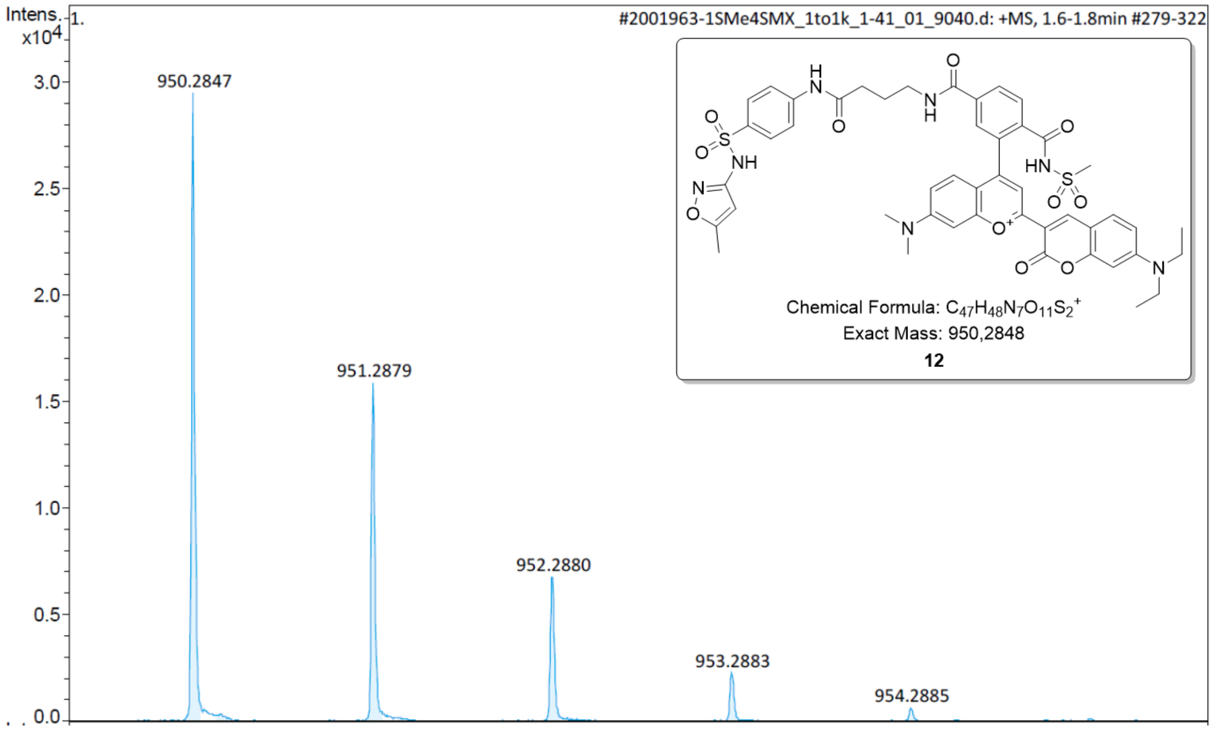
Intens.
x10^4
3.0
2.5
2.0
1.5
1.0
0.5
0.0
1.
#2001963-1SMe4SMX_1to1k_1-41_01_9040.d: +MS, 1.6-1.8min #279-322
950.2847
951.2879
952.2880
953.2883
954.2885
Chemical Formula: C47H48N7O11S2+
Exact Mass: 950,2848
12
